# Synthesis and Biological Activity of Azolo[*a*]quinoxalines

**DOI:** 10.3390/molecules31152592

**Published:** 2026-07-24

**Authors:** Emiliya V. Nosova, Galina N. Lipunova, Valery N. Charushin

**Affiliations:** 1 Postovsky Institute of Organic Synthesis, Ural Branch of the Russian Academy of Sciences, 22 S. Kovalevskaya St./20 Akademicheskaya St., Ekaterinburg 620137, Russia; lipunova@ios.uran.ru (G.N.L.); charushin@ios.uran.ru (V.N.C.); 2 Department of Organic and Biomolecular Chemistry, Ural Federal University, 19 Mira St., Ekaterinburg 620002, Russia

**Keywords:** azolo[*a*]quinoxalines, heterocyclization, anticancer activity, enzyme and receptor inhibitors, structure-activity relationships, in silico drug design, multicomponent reactions

## Abstract

This review covers published data (mostly from 2019 to 2025) on the synthesis and biological activity of azolo[*a*]quinoxalines, including pyrazolo-, imidazo- and triazolo-annelated systems. We highlight that most research efforts are directed toward the design of anticancer agents, with additional applications as Toll-like receptor antagonists, monoamine oxidase inhibitors, opioid receptor modulators, PI3Kα inhibitors, tubulin polymerization inhibitors, GABAᴀ receptor modulators, VEGFR-2 kinase inhibitors, BRD9 binders, and anti-inflammatory, antimicrobial, and antifungal agents. Recent synthetic strategies include Cu-catalyzed oxidative annulations, I_2_-mediated C–H functionalization, metal-free cascade cyclization, and multicomponent reactions, often employing eco-friendly catalysts and reductants. A growing number of studies integrate virtual screening, molecular docking, and pharmacophore-based in silico approaches to guide lead discovery and optimization. Innovative drug delivery systems, such as nanogels and hybrid molecules combining azoloquinoxalines with pharmacophores like thalidomide, have also been explored. This review emphasizes both the medicinal chemistry aspects of azolo[*a*]quinoxalines and the synthetic methodologies for their preparation from the perspective of drug development and discovery.

## 1. Introduction

Quinoxaline and its derivatives represent a significant class of nitrogen-containing heterocycles that are widely recognized for their diverse and potent biological activities [1,2,3]. The structural versatility of the quinoxaline core allows for the modulation of its pharmacological profile through various annelation strategies, making it an attractive scaffold in medicinal chemistry and drug discovery. Numerous natural products and synthetic compounds containing the quinoxaline moiety have been reported to exhibit anticancer, antimicrobial, anti-inflammatory, and neuroactive properties, among others [4]. For instance, naturally occurring quinoxaline antibiotics such as echinomycin and triostin A are well-known for their potent antitumor activity through DNA intercalation and inhibition of transcription [5,6,7]. These examples underscore the potential of the quinoxaline framework as a privileged structure for the development of novel therapeutic agents.

The annelation of an additional azole ring at the [*a*] bond of the quinoxaline nucleus represents a particularly important modification, as it can significantly influence the physicochemical properties and biological activity of the resulting heterocyclic system. Azolo[*a*]quinoxalines, which combine a quinoxaline core with a fused five-membered nitrogen-containing ring (pyrazole, imidazole, triazole), have emerged as promising candidates for drug development. Over the past decade, a substantial body of research has been devoted to the synthesis and biological evaluation of these fused systems, leading to the identification of compounds with activity against various therapeutic targets, including kinases [8], Toll-like receptors (TLRs) [9], monoamine oxidases (MAOs) [10], opioid receptors [11], and GABA_A_ receptors [12].

Several recent reviews have addressed the anticancer potential of quinoxaline derivatives. For instance, a comprehensive overview covering the 2015–2024 period summarized the efficacy of diverse quinoxaline-based compounds as inhibitors of cancer-related pathways and enzymes [1]. Another work by Nafie et al. [13] focused on the anticancer activity of quinoxaline and other benzodiazines, with an emphasis on kinase inhibition. Montero and co-workers [14] systematically examined the anticancer mechanisms of quinoxaline derivatives, including their effects on the cytoskeleton (tubulin polymerization inhibition), DNA impairment, regulation of cell metabolism (folate antagonism, ROS modulation, PPAR-γ targeting), and inhibition of proliferation pathways.

**Figure 1:**
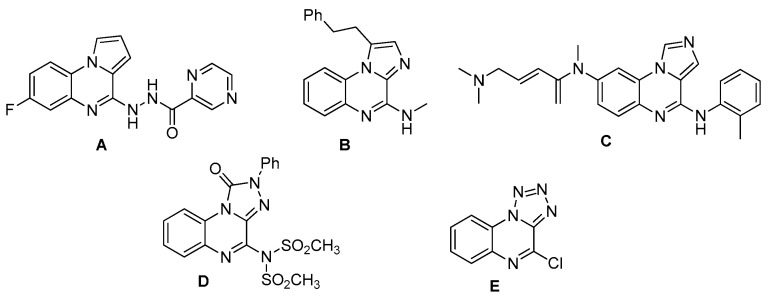
Representative examples of biologically active [*a*]-annelated quinoxalines from the literature [15]. (**A**) potent antiproliferative agent against multiple cancer cell lines; (**B**) tubulin polymerization inhibitor with anti-melanoma activity; (**C**) irreversible BTK inhibitor for rheumatoid arthritis treatment; (**D**) selective human A3 adenosine receptor antagonist; (**E**) Syk kinase inhibitor with anti-allergic properties. These compounds are research tools and are not clinically approved drugs; they are shown to illustrate the structural diversity and biological potential of the azolo[*a*]quinoxaline scaffold.

Patinote et al. [15] in 2021 systematically summarized the data on the biological activity of fused azolo-quinoxalines containing one to four nitrogen atoms in the fused five-membered ring. The authors [15] analyzed around 150 derivatives and general structures with promising results; the most frequently identified types of biological activity for these compounds were antitumor, neuroactive, and antimicrobial effects. As noted in the review, the pyrrolo[1,2-*a*]quinoxaline and [1,2,4]triazolo[4,3-*a*]quinoxaline scaffolds were described far more extensively than the pyrazolo[1,5-*a*]quinoxaline and [1,2,4]triazolo[1,5-*a*]quinoxaline cores. This difference directly reflects the chemical accessibility of the target heterocyclic systems and, consequently, the diversity of substituted derivatives prepared and the range of research themes explored (Figure 1).

**Figure 2:**
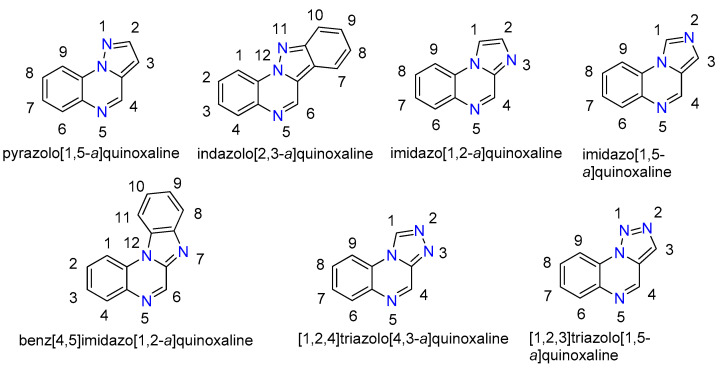
Types of [*a*]-annelated quinoxalines considered in the current review. Systematic atomic numbering follows IUPAC rules for angular fused heterocycles: molecules are oriented with the maximum number of rings horizontally; bridgehead nitrogen atoms receive locants, while bridgehead carbon atoms are denoted by letters; numbering proceeds clockwise from the atom adjacent to the fusion point. This figure serves as a structural reference for all subclasses discussed in Section 2, Section 3, Section 4 and Section 5.

The current manuscript contains data on the synthesis and biological activity of azolo[*a*]quinoxalines, which were not included in the review [15] or appeared later. In this review, we focus on the medicinal chemistry aspects of various azolo[*a*]-annelated quinoxalines (Figure 2), encompassing both synthetic pathways and biological activities. We compile relevant literature reports within the context of drug development and discovery efforts.

## 2. Pyrazolo[1,5-a]quinoxalines

Pyrazoles represent an important class of heterocyclic compounds exhibiting a wide range of biological properties. They have quickly attracted the attention of researchers, and several reviews have been devoted to the synthesis of pyrazole derivatives and their bioactivity (for example, [16,17]). It has been noted that the condensation of a pyrazole ring with another heterocycle generally leads to increased biological activity. The review article [18] reported progress in the synthesis of compounds in which pyrazole is attached to five-, six- or seven-membered heterocycles. The reviews [19,20] discussed publications concerning such biological properties of pyrazole derivatives as analgesic and anti-inflammatory, antibacterial, antitumoral, antiviral and others. A review article on annulated azolo-quinoxalines as promising compounds for medicinal chemistry [15] also contains a section on pyrazolo[1,5-*a*]quinoxalines. The general structure of pyrazolo[1,5-*a*]quinoxalines is shown in Figure 2. This section covers studies on the synthesis and properties of pyrazoloquinoxalines that were not included in review [15] or have been published thereafter.

**Scheme 1:**
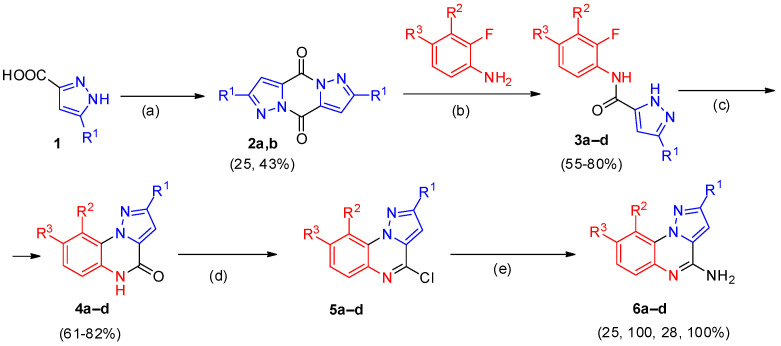
Synthesis of pyrazolo[1,5-*a*]quinoxalines **6a–d**: (**a**) SOCl_2_, reflux, 18 h; (**b**) NaHMDS, THF, 5 h; (**c**) NaH, DMA, reflux, 48 h; (**d**) POCl_3_, DEA, MW, 130 °C, 15 min; (**e**) NH_4_OH (30%), CAN, MW, 140 °C, 2 h. **6a**: R^1^ = butyl, R^2^ = R^3^ = H; **6b**: R^1^ = isobutyl, R^2^ = R^3^ = H; **6c**: R^1^ = butyl, R^2^ = Me, R^3^ = H; **6d**: R^1^ = butyl, R^2^ = H, R^3^ = Me. Color coding: blue—pyrazole fragment; red—quinoxaline core.

Toll-like receptors (TLRs) constitute a class of single-transmembrane cellular receptors that recognize conserved microbial structures and trigger innate immune responses. Among these, TLR7 and TLR8 play important roles in immune system activation. Bou Karroum et al. [21] described novel and selective TLR7 antagonists within the series of pyrazolo[1,5-*a*]quinoxalines **6**. The synthesis of pyrazolo[1,5-a]quinoxaline analogs of **6** was carried out according to Scheme 1. The reaction of commercially available 5-butyl and 5-isobutyl-1H-pyrazole-3-carboxylic acids **1** using thionyl chloride led to condensed dimer intermediates **2a,b**; treatment of **2a,b** with substituted *o*-fluoroanilines in THF in the presence of sodium bis(trimethylsilyl)amide afforded pyrazoles **3**. Subsequent intramolecular cyclization of amides **3** under strongly basic conditions produced pyrazoloquinoxalinones **4**. Compounds **4a–d** were then converted into chlorinated intermediates **5a–d** upon reaction with phosphorus oxychloride and N,N-diethylaniline. Finally, nucleophilic substitution of chlorine with aqueous ammonia gave the target pyrazolo[1,5-*a*]quinoxalin-4-amines **6a–d**.

The inhibitory potential of compounds **6a–d** toward TLR7 and TLR8 was evaluated in vitro using HEK-Blue™-hTLR7 and HEK-Blue™-hTLR8 cells [21]. At concentrations up to 100 μM, all tested compounds exhibited sufficiently low cytotoxicity as determined by the MTT (3-(4,5-dimethylthiazol-2-yl)-2,5-diphenyltetrazolium bromide) assay. None of the derivatives showed any agonistic activity on either TLR7 or TLR8. However, compounds **6a–d** demonstrated selective TLR7 antagonistic effects without affecting TLR8 across the concentration range studied. The half-maximal inhibitory concentrations (IC_50_ values) for TLR7 are summarized in Table 1. Derivatives **6a** and **6b**, which bear a butyl and an isobutyl chain at position 2, respectively, and have hydrogen atoms at positions 8 and 9, showed TLR7 antagonistic activity with low micromolar IC_50_ values. The introduction of a methyl group at position 8 (compound **6d**) resulted in decreased activity, while the presence of a methyl group at position 9 (compound **6c**) led to an even more pronounced reduction in potency.

**Figure 3:**
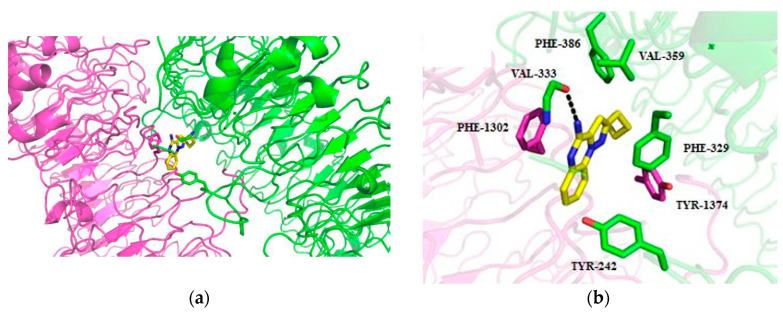
(**a**) Overall structure of TLR7 with compound **6a** bound to the antagonist site; (**b**) Structure of human TLR7 ectodomain in complex with compound **6a.** Color coding: magenta and green for TLR7 monomers, nitrogen atoms blue, oxygen atoms red, and carbon atoms yellow for compound **6a**. Reproduced with permission of the American Chemical Society [21].

The selectivity was further corroborated by a comparative ligand-docking study into the TLR7 antagonist binding pocket using compound **6a** as an example. The modeling investigation performed on TLR7 revealed the presence of a previously unrecognized antagonist binding site, analogous to that earlier described for TLR8 [22]. This site is located at the dimerization interface and accommodates antagonists via multiple chemical interactions (Figure 3a). Additionally, an extra van der Waals contact was observed between the benzene ring and residue TYR-242 (Figure 3b). In their concluding remarks, the authors [21] noted that compounds **6a** and **6b**, acting as potent and selective TLR7 antagonists, hold promise for the development of new immunomodulatory agents.

**Scheme 2:**
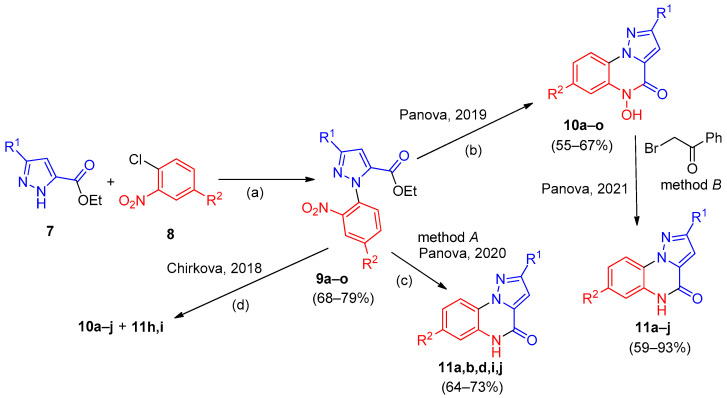
Synthesis of pyrazolo[1,5-*a*]quinoxalin-4-ones **10a–o, 11a–j**: (**a**) K_2_CO_3_, DMF, 50–80 °C, 3–6 h; (**b**) SnCl_2_, HCl, EtOH, 50 °C, 3–4 h; (**c**) Pd/C, H_2_; (**d**) Zn/NH_4_Cl. **7**, **9**, **10**: R^1^ = Ph, 4-ClC_6_H_4_, 4-MeC_6_H_4_, 4-MeOC_6_H_4_; **8**, **9**, **10**: R^2^ = CF_3_, CN, NO_2_, COOEt (for **9** and **10a–k**), NH_2_ (for **10L–o**); **11**: R^1^ = Ph, 4-MeC_6_H_4_, 4-MeOC_6_H_4_, 4-ClC_6_H_4_; R^2^ = COOEt, CN, NH_2_. For specific substituent combinations, see Table 2 and the corresponding text discussion. Color coding: blue—pyrazole fragment; red—quinoxaline core [23,24,25,26].

Panova and co-workers [23,24,25] developed synthetic approaches to substituted pyrazolo[1,5-*a*]quinoxalin-4-ones **10** and **11** using different strategies. A two-step synthesis of 5-hydroxypyrazolo[1,5-*a*]quinoxalin-4-ones **10a–o** was described [23,24]. Arylation of pyrazoles **7** with nitrochloroarenes **8** in DMF in the presence of K_2_CO_3_ under heating afforded a broad range of N-aryl-substituted pyrazoles **9a–o** (Scheme 2). Reduction of the nitro group in intermediates **9** with tin(II) chloride in an acidic ethanolic medium, followed by spontaneous intramolecular cyclization, gave the desired substituted pyrazoloquinoxalinones **10a–o** containing an N-OH moiety [23].

In a subsequent report [24], the synthesis of pyrazolo[1,5-*a*]quinoxalin-4-ones **11** was achieved by reduction of intermediates **9** using 10% palladium on carbon (method A). High yields were obtained when the reaction was run in methanol as a solvent at 71–73 °C under a hydrogen pressure of 4–5 atm. However, isolation of the target products from the reaction mixture proved difficult owing to their very low solubility in organic solvents, except for DMF and DMSO. Only compounds **11a,b,d,i,j** were obtained in good yield and fully characterized.

Reduction of compounds **9** with zinc in an aqueous tetrahydrofuran solution, following the conditions described [26], was also attempted to prepare analogs **10a–j** (Scheme 2). In the case of dinitro compounds **9**, carrying out the reaction at 50–60 °C resulted in an inseparable 1:1 mixture of products **10** and **11** as judged by NMR spectroscopy. Overall, the zinc reduction gave lower yields of compounds **10** due to the formation of byproducts compared to the method employing SnCl_2_.

An alternative route to pyrazolo[1,5-*a*]quinoxalin-4-ones **11a–j** (method B) was proposed via N-dehydroxylation of pyrazoloquinoxalines **10** [24,25] (Scheme 2). Treatment of compounds **10** with phenacyl bromide in methanol in the presence of triethylamine at 40–60 °C for 2–8 h furnished the desired products **11a–j** in up to 93% yield. The operational simplicity and catalyst-free conditions of this approach make it highly attractive for the preparation of diverse pyrazolo[1,5-*a*]quinoxalines with potential biological relevance.

**Scheme 3:**
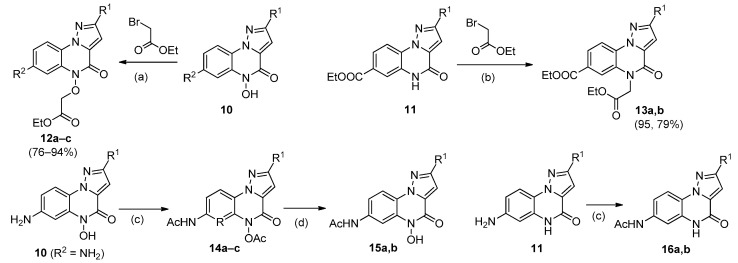
Synthetic approaches to pyrazolo[1,5-*a*]quinoxalin-4-ones **12**–**16**: (**a**) Et_3_N, MeOH; (**b**) K_2_CO_3_, DMF; (**c**) Ac_2_O/Py, 100 °C; (**d**) piperidine, EtOH, then AcOH. **12**: R^1^ = 4-MeC_6_H_4_, 4-MeOC_6_H_4_; R^2^ = COOEt, CN; **13**–**16**: R^1^ = Ph, 4-MeOC_6_H_4_, 4-ClC_6_H_4_. For specific substituent combinations, see Table 2 and the corresponding text discussion. No color coding is used as this scheme illustrates the functionalization of the preformed polycyclic core.

A series of functionalized analogs have been synthesized starting from pyrazolo[1,5-*a*]quinoxalin-4-ones **10** and **11**. The authors [24] noted that replacing phenacyl bromide with ethyl bromoacetate under the same conditions allowed the isolation of alkylation products **12a,b** from compounds **10** in high yield (Scheme 3). In contrast, derivatives **11**, unlike their hydroxy-containing counterparts **10**, did not react with ethyl bromoacetate under these conditions. For compounds **11**, the reaction was feasible in DMF using K_2_CO_3_ as a deprotonating agent, giving rise to alkyl derivatives **13a,b**.

Acylation with acetic anhydride was also performed on the amino-substituted pyrazoloquinoxalinones **10** and **11** [25]. In the case of compounds **10**, diacylated products **14** were isolated. These were subsequently hydrolyzed in ethanol in the presence of piperidine to afford derivatives **15**. Under analogous conditions, compounds **11** yielded products **16** acylated exclusively at the amino group. The structures of all synthesized compounds were confirmed by a combination of spectroscopic techniques, including single-crystal X-ray diffraction analysis performed on compound **10d** [25].

In the study [25], a large set of pyrazolo[1,5-*a*]quinoxalin-4-ones (22 compounds), shown in Scheme 2 and Scheme 3, were evaluated as inhibitors of monoamine oxidase (MAO) following the described protocol [27]. MAOs constitute a family of enzymes that catalyze the oxidation of bioactive amines, a process associated with the development of neuropsychiatric and neurodegenerative disorders. Inhibitors can block the action of these enzymes. The investigations employed two recombinant human MAO isoforms (MAO-A and MAO-B), with kynuramine (diaminopropiophenone) serving as a substrate for both isoforms. MAO catalytic activity was determined by measuring the amount of 4-hydroxyquinoline formed upon MAO-catalyzed oxidation of kynuramine using fluorescence spectrophotometry (λ_em_ = 400 nm).

The obtained IC_50_ values for MAO-A and MAO-B inhibition showed that the tested pyrazolo[1,5-*a*]quinoxalin-4-ones inhibited both isoforms with varying degrees of selectivity. Only eight compounds inhibited MAO-A with IC_50_ values below 1 μM, whereas three compounds displayed MAO-B inhibition with IC_50_ < 1 μM (Table 2). It was noted that all acylated pyrazolo[1,5-*a*]quinoxalin-4-ones (**14**–**16**) exhibited MAO-A inhibition with submicromolar IC_50_ values. Within this series, product **14c** showed the highest potency, with an IC_50_ value of 0.028 μM. Among the non-acylated amino compounds (**10L–o**), only one compound (**10L**, with R^2^ = 4-ClC_6_H_4_) had an IC_50_ below 1 μM, whereas in the acylated series, submicromolar activity was found for compounds with various R^2^ substituents [25]. The best MAO-B inhibition was demonstrated by pyrazolo[1,5-*a*]quinoxalin-4-one **11f**, with an IC_50_ value of 0.617 μM. Taken together, the findings revealed high potency and selectivity for MAO-A inhibition among the pyrazolo[1,5-*a*]quinoxalin-4-ones.

To gain insight into the binding modes and interactions of the studied compounds with the active sites of MAO, molecular docking experiments were performed [25] on three selected compounds (**10L**, **11d**, and **14c**). Using molecules **10L** and **14c** as examples, the authors [25] discussed their comparative binding to MAO-A in relation to structural features. They noted that the most potent MAO-A inhibitor **14c** does not fit into the MAO-A active site in the manner suggested by the molecular docking experiments. In their concluding remarks, the authors [25] noted that this is the first report on MAO inhibition by pyrazoloquinoxalinone analogs and that these compounds appear promising for the development of MAO inhibitors, particularly those targeting the MAO-A isoform.

Yadav and co-workers [11] reported the synthesis of a broad range of pyrazolo[1,5-*a*]quinoxalin-4-ones **19** as opioid receptor modulators. Opioid receptors are a class of nervous system receptors whose primary physiological role is the regulation of pain perception. Dysregulation of these receptors can lead to drug abuse and other psychiatric disorders. Three main classes of opioid receptors are known: KOR (κ-opioid receptor), MOR (μ-opioid receptor), and DOR (δ-opioid receptor), each of which functions differently in the body.

**Scheme 4:**
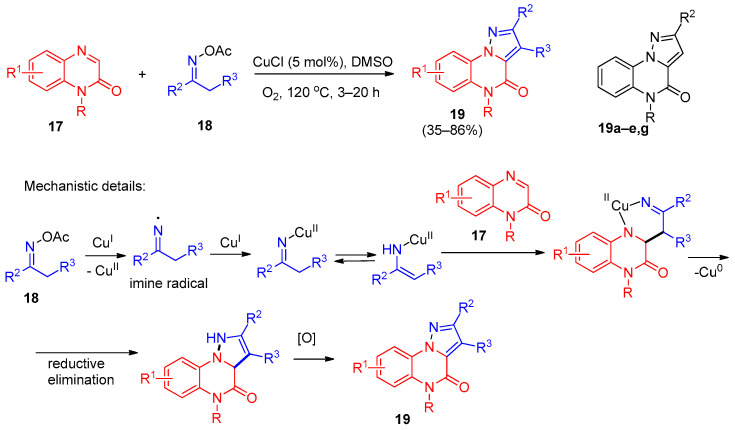
Synthesis of pyrazolo[1,5-*a*]quinoxalin-4-ones **19**. R^2^ = Ph, R = Me (**19a**), Et (**19b**), *^n^*Pr (**19c**), *^n^*Bu (**19d**), allyl (**19e**); R = CH_2_COOEt, R^2^ = 4-MeOC_6_H_4_ (**19g**). Color coding: blue—pyrazole fragment; red—quinoxaline core.

The synthesis of target products **19** was accomplished via a copper-catalyzed oxidative [3+2] annulation using readily available quinoxalin-2-ones **17** and oxime acetates **18** (Scheme 4). Using compound **19a** (R = Me; R^1^, R^2^, R^3^ = H) as a model substrate, the optimal reaction conditions for achieving high yields of the target products were established. The structures of compounds **19** were confirmed by a combination of spectroscopic methods, including single-crystal X-ray diffraction analysis for **19a**. Based on literature precedents and control experiments, the authors [11] proposed a plausible reaction mechanism.

Using a copper-catalyzed oxidative [3+2] annulation of quinoxalin-2(1*H*)-ones with oxime-*O*-acetates, the authors prepared a library of 45 functionalized pyrazolo[1,5-*a*]quinoxalin-4(5*H*)-ones **19** [11]. The method has several practical perks: the starting materials are cheap and easy to make; regioselectivity is excellent (only one regioisomer); the scope is broad (*N*-alkyl, allyl, propargyl, benzyl, and aryl groups all work, along with halogenated, electron-rich, and electron-poor quinoxalinones, plus cyclic and heteroaromatic oxime acetates); and the reaction works on a gram scale. Best of all, it avoids hydrazine—a carcinogen—which gives it a clear edge over classical methods that rely on hydrazine and are limited in substrate diversity.

**Figure 4:**
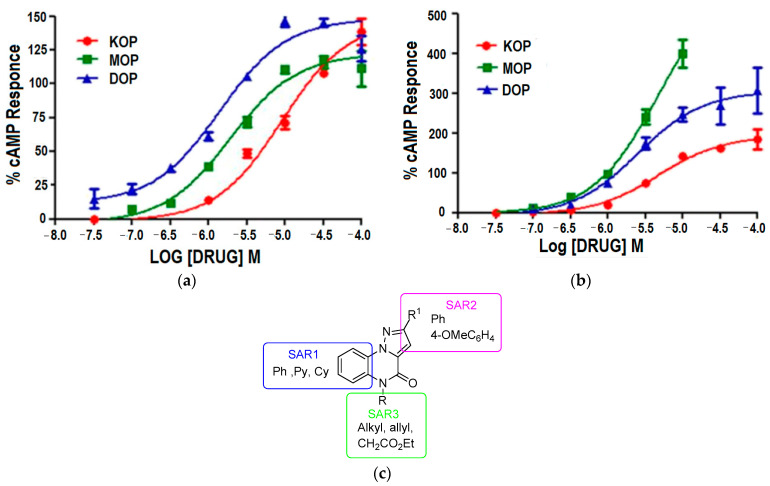
Opioid receptor antagonist activity of active compounds (**a**) **19c**; (**b**) **19e**; sites for structure diversification of compounds **19** under SAR1, SAR2, and SAR3 (**c**). Reproduced with permission of the American Chemical Society [11].

All synthesized compounds **19** were evaluated for antagonistic and agonistic activities at KOR, MOR, and DOR opioid receptors by measuring their effect on cAMP accumulation in HEK293T cells expressing KOR, MOR, or DOR. The studies revealed that eight compounds from series **19** exhibited antagonistic effects on two or three opioid receptors with varying EC_50_ values (Table 3). As can be seen from the data, among these eight, only two derivatives (**19c** and **19e**) were effective at all three receptors, with both being more potent at MOR and DOR than at KOR. The highest potency was observed for compound **19c** at DOR (1.47 μM). A comparison of the data obtained for compound **19e** with those of reference antagonists (naloxone for MOP (μ-opioid protein), naltrexone for DOP (δ-opioid protein), and GNTI for KOP (κ-opioid protein)) showed that the E_m_ₐ_x_ values for **19e** were two to three times higher than those of the reference antagonists, indicating significant antagonistic properties, although the EC_50_ values for all three receptors were rather modest. The dependence of the cAMP response (%) on the concentration of compounds **19c** or **19e** is illustrated in Figure 4. Derivatives **19d** and **19g** displayed selectivity for DOP, albeit with only moderate EC_50_ values. Within the series of compounds **19a–e**, a clear effect of the substituent R on potency was observed. The highest activity was achieved with *n*-propyl (**19c**) and allyl (**19e**) substituents, both of which contain a three-carbon chain. Increasing the chain length to butyl (**19d**) resulted in a significant reduction in activity (EC_50_ > 10 μM at KOR and MOR, and 5.60 μM at DOR), while shortening the chain to ethyl (**19b**) or methyl (**19a**) also led to decreased potency across all three receptors. These findings suggest that a three-carbon linker (either saturated *n*-propyl or unsaturated allyl) is optimal for opioid receptor antagonism, with the allyl group being particularly effective at MOR (EC_50_ = 1.82 μM, E_m_ₐ_x_ = 390%) and DOR (EC_50_ = 2.49 μM, E_m_ₐ_x_ = 312%).

A comparison of compounds **19a** and **19f** showed that introduction of a nitrogen atom into the benzene ring led to a significant reduction in activity while maintaining the receptor preference profile [11].

Based on the results presented in Table 3, a structure–activity relationship (SAR) analysis was performed (Figure 4c). The influence of substituents in various structural fragments of compounds **19a–h** on antagonistic activity is indicated as SAR1, SAR2, and SAR3. It was found that Ph, Py, and Cy cycles are preferred for activity (SAR1); Ph and 4-MeOC_6_H_4_ in SAR2; and alkyl, allyl, and CH_2_COOEt in SAR3 are also more effective substituents compared to others.

For the KOR-active compounds **19c** and **19e**, the authors ran docking studies with the hKOR protein to see how they bind [11]. No hydrogen bonds turned up in the active site pocket itself, but they did spot one between the quinoxalinone oxygen and the T111 residue.

So, a series of pyrazolo[1,5-*a*]quinoxalin-4-ones **19** displayed excellent antagonistic activity toward the human KOR, MOR, and DOR opioid receptors [11].

**Figure 5:**
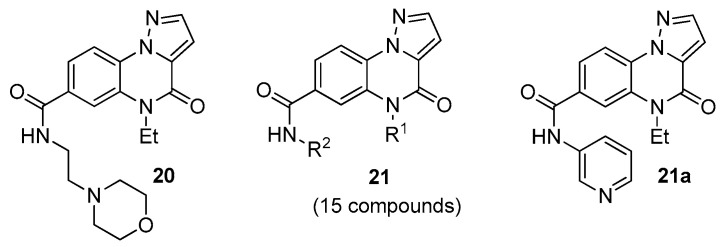
Structure of compounds **20**, **21**; **21**: R^1^ = Et, Me, Bz, 4-F-benzyl; R^2^ = morpholine, piperidine, piperazine, dimethylamino, cyclohexane, ether, benzene, pyridine.

Gu and co-workers reported the discovery of new PI3Kα inhibitors through virtual screening of commercial and in-house chemical libraries [28,29,30]. Phosphoinositide 3-kinase (PI3K) plays a key role in regulating numerous cellular processes. One of its isoforms, PI3Kα, has attracted particular attention because mutations in the PIK3CA gene, which encodes PI3Kα, have been found to underlie up to 30% of human cancers. Consequently, the search for new PI3Kα inhibitors remains a pressing challenge. As a result of the virtual screening campaign, pyrazolo[1,5-*a*]quinoxalin-4-one **20** was identified as a PI3Kα inhibitor with an IC_50_ value of 10.92 μM (Figure 5). Fifteen commercially available compounds **21** (analogs of **20**) were selected from a commercial library and acquired for biological evaluation.

The in vitro PI3Kα inhibitory activities obtained were used to perform a SAR analysis of compounds **20** and **21**. It was shown that compounds bearing a piperazine group at R^2^ displayed no activity. The remaining analogs inhibited PI3Kα with IC_50_ values ranging from 3.52 to 12.25 μM. The highest activity was demonstrated by compound **21a**, with an IC_50_ of 3.52 μM, which was selected for further modification based on docking results; several modification strategies were proposed, including variation in the R^1^ and R^2^ substituents in **21**, replacement of the pyrazole ring with other heterocycles, and introduction of an amino substituent into the quinoxalinone benzene ring to provide additional binding interactions with PI3Kα.

**Scheme 5:**
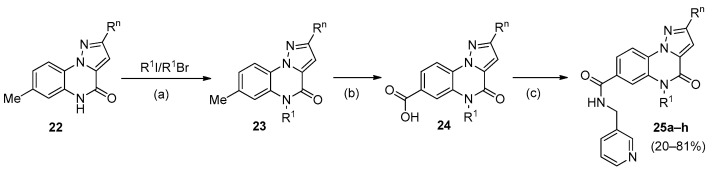
Synthesis of pyrazolo[1,5-*a*]quinoxalin-4-ones **25a–h**: (**a**) NaH, DMF, room t.; (**b**) KMnO_4_, *t*BuOH/H_2_O, 100 °C; (**c**) HOBT, EDCl, TEA, DCN, room t. **25**: R^n^ = H, R^1^ = *n*-Bu (**a**), 4-cyclopropylmethyl (**b**), 4-trifluoromethoxybenzyl (**c**), 4-trifluoromethoxybenzyl (**d**), 4-*t*-butylbenzyl (**e**), 4-trifluoromethylbenzyl (**f**); R^n^ = Me, R^1^ = Et (**g**); R^n^ = *i*Pr, R^1^ = Et (**h**). No color coding is used as this scheme illustrates functionalization of the preformed polycyclic core.

Following a procedure analogous to that shown in Scheme 1 but using substituted pyrazole-3-carboxylic acids and 2-fluoro-5-methylaniline as starting materials, pyrazolo[1,5-*a*]quinoxalin-4-ones **22** were prepared and subsequently converted into derivatives **25a–h** (Scheme 5). These compounds were evaluated for PI3Kα inhibitory activity. Only compounds **25a** and **25b** showed activity, with low IC_50_ values of 2.00 and 2.65 μM, respectively. Introduction of 4-substituted benzyl group led to a complete loss of activity (**23c–23f**, IC_50_ > 20 μM). Substitution on the pyrazole ring also reduced activity (**23g**, IC_50_ = 11.36 μM; **23h**, IC_50_ > 20 μM). For further optimization, the ethyl group was retained at the R^1^ position.

**Figure 6:**
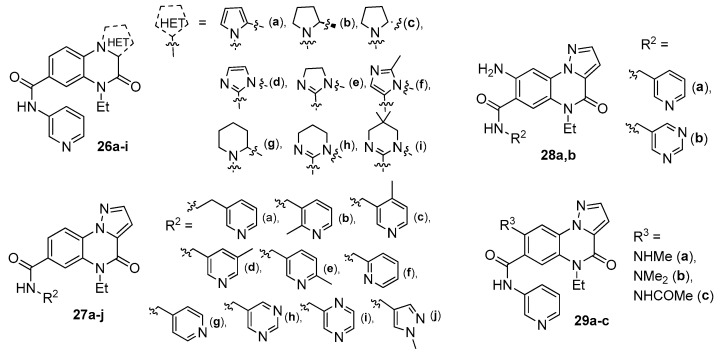
Structure of compounds **26a–i**, **27a–j**, **28a,b**, **29a–c**.

To examine the influence of the heterocyclic core on activity, a series of hetarylquinoxalinones **26a–i** was synthesized (Figure 6). All compounds were tested for PI3Kα inhibitory activity. Only the analog containing a dihydroimidazole fragment (**26e**) showed activity, with an IC_50_ value of 3.63 μM, which was close to that of compound **21a** (IC_50_ = 3.52 μM). The use of other heterocycles resulted in loss of activity (IC_50_ > 20 μM). Comparison of the IC_50_ data for molecules **21a**, **26a** and **26e** led to the conclusion that the position of the second nitrogen atom in the pyrazole or imidazole ring plays a decisive role in PI3Kα binding.

The effect of the R^2^ substituent on PI3Kα inhibitory activity was studied using a series of pyrazolo[1,5-*a*]quinoxalin-4-ones **27a–j** (Figure 6). Analysis of the obtained data showed that all derivatives exhibited activity with IC_50_ values ranging from 1.59 μM to 13.91 μM. Increasing the carbon chain length at R^2^ led to a significant decrease in activity (**27a**, IC_50_ = 13.91 μM); thus, a methylene linker proved optimal. Changing the position of the methyl group or the nitrogen atom in the pyridine ring had little effect on activity (**27c–g**). Switching from pyridine to 5-methylpyrimidine resulted in a twofold increase in activity (**27h**, IC_50_ = 1.59 μM), whereas the transition to pyrazine was accompanied by a slight decrease.

The effect of an amino substituent introduced into the quinoxalinone core was studied using compounds **28** and **29** (Figure 6), whose PI3Kα inhibitory activities are presented in Table 4. As can be seen from these data, the activity of compounds **28a** and **28b** was approximately five and fifteen times higher, respectively, than that of **21a** (IC_50_ = 3.52 μM), the compound from which they were derived through structural modification. Both compounds displayed PI3K inhibitory activity against all four isoforms. Notably, pyrazoloquinoxaline **28b** showed isoform selectivity over the other three Class I PI3K subtypes (Table 4) and exhibited improved activity compared to lead compound **21a**. A docking study of compound **28a** with PI3Kα revealed that the introduced amino group formed a hydrogen bond with the carbonyl group of Val851.

Compound **28b** was further evaluated in T47D tumor cells to assess its ability to inhibit PI3K signaling. It demonstrated moderate concentration-dependent inhibitory activity toward Akt phosphorylation. In experiments using Kasumi-1 and T47D cells, compound **28b** showed activity with IC_50_ values of 1.64 and 1.82 μM, respectively.

**Scheme 6:**
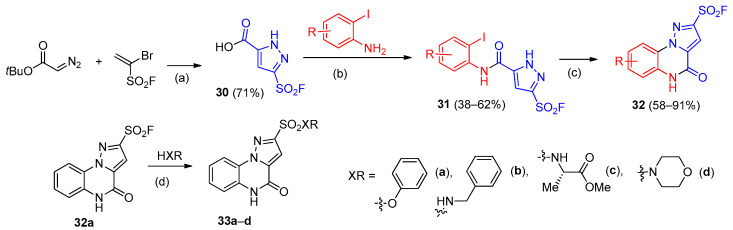
Synthesis of pyrazolo[1,5-*a*]quinoxalin-4-ones **32**, **33a–d**: (**a**) MeCN, 1 h, then TEA, DCM, 12 h; (**b**) SOCl_2_, 80 °C, then pyridine, DMAP; (**c**) Cu_2_O, K_2_CO_3_, 1,10-phenanthroline, toluene, 150 °C, 17 h; (**d**) DABCO, Ca(NTf_2_)_2_, MeCN. **32**: R = H, Me, F_3_C, Cl, OMe. Color coding: blue—pyrazole fragment; red—quinoxaline core.

The attractiveness of pyrazolo[1,5-*a*]quinoxalin-4-ones for medicinal chemistry continues to drive the search for new synthetic routes to this class of compounds. Khanal et al. [31] developed an approach to pyrazolo[1,5-*a*]quinoxalin-4-ones bearing a sulfonyl fluoride group. The presence of this functionality allows reactions to be carried out under mild conditions with a wide range of nucleophilic groups, thereby expanding the diversity of accessible compounds. In the first step, 3-(fluorosulfonyl)-1*H*-pyrazole-5-carboxylic acid **30** was obtained via a 1,3-dipolar cycloaddition of 1-bromoethene-1-sulfonyl fluoride with a diazoacetate (Scheme 6). Treatment of acid **30** with thionyl chloride followed by reaction with a substituted *o*-iodoaniline afforded 5-carbamoyl-1*H*-pyrazole-3-sulfonyl fluorides **31**. Cyclization of these intermediates under optimized conditions gave 4-oxo-4,5-dihydropyrazolo[1,5-*a*]quinoxaline-2-sulfonyl fluorides **32** in yields ranging from 58 to 91%. Using molecule **32a** as an example, the ability of the sulfonyl fluoride group to undergo exchange with O- and N-nucleophiles to furnish compounds **33a–d** was demonstrated (Scheme 6).

**Scheme 7:**
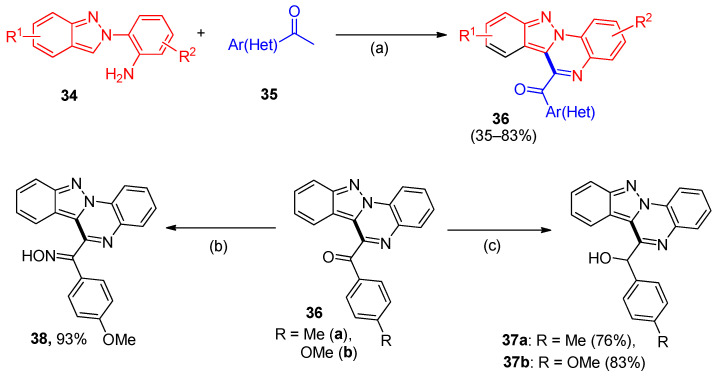
Synthesis of indazolo[2,3-*a*]quinoxalin-6-yl(aryl)methanones **36**–**38**: (**a**) I_2_, TFA, DMSO, 110 °C; (**b**) NH_2_OH, HCl, pyridine, 60 °C, 7 h; (**c**) NaBH_4_, EtOH, room t., 4 h. R^1^ = H, Br; R^2^ = H, 2,3-diMe, 2,3-diCl; Ar(Het) = Ph, 2-R^3^C_6_H_4_, 3-R^3^C_6_H_4_, 4-R^3^C_6_H_4_ (R^3^ = H, Me, OMe, F, Br, CN, NO_2_, OBn, SMe), thiophene-2-yl, benzo[*b*]thiophene-2-yl, indol-3-yl. Color coding: blue—acetophenone or acetylheterocycle fragment; red—2-(2H-indazol-2-yl)aniline fragment.

Indazolo[2,3-*a*]quinoxalines, which possess a planar polyaromatic structure, have attracted attention as important pharmacophores. They exhibit antitumor effects due to their ability to intercalate. However, synthetic approaches to these compounds are rather limited and often rely on transition metal catalysts. Arockiaraj et al. [32] described a metal-free, iodine-mediated method for the synthesis of indazoloquinoxalines. Using 2-(2*H*-indazol-2-yl)anilines **34** and aryl methyl ketones **35** as starting substrates (Scheme 7), the optimal conditions were found to give the highest yields of indazolo[2,3-*a*]quinoxalin-6-yl(aryl)methanones **36** when using a **34:35**:I_2_ molar ratio of 1:1:1 with 3 equivalents of TFA in 2 mL of DMSO at 110 °C. In addition to aryl ketones, naphthyl- and heteroaryl methyl ketones were also successfully employed in the reaction. The method allows access to a broad range of target products, is straightforward to perform, and shows good scalability. The authors [32] carried out control experiments and discussed a plausible reaction mechanism.

Further transformations were performed using products **36** (R = Me, OMe) as examples. Reduction of the keto group afforded alcohols **37a,b**, while reaction with hydroxylamine gave the oxime-containing derivative **38** (Scheme 7). All synthesized indazoloquinoxalines were characterized by ^1^H and ^13^C NMR spectroscopy, and the structure was confirmed by single-crystal X-ray diffraction analysis for the derivative of **36** bearing a naphthyl fragment.

**Scheme 8:**
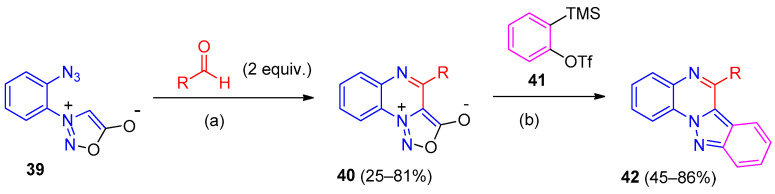
Synthesis of indazolo[2,3-*a*]quinoxalines **42**: (**a**) PPhMe_2_ (1.1 equiv.), CuOTf_2_ (2 equiv.), MeCN, room t.; (**b**) TBAF, MeCN, room t. **40**, **42**: R = *i*Pr, 4-CNC_6_H_4_, 4-CNC_6_H_4_, 4-MeCOOC_6_H_4_, 3-PhC_6_H_4_. Color coding: blue—azido-sydnone fragment; red—aldehyde fragment; marengo—aryne fragment.

A new approach to the synthesis of indazoloquinoxalines, based on a [3+2] cycloaddition of tricyclic sydnones containing a quinoxaline moiety with arynes, was proposed [33]. Quinoxaline-sydnones **40** were obtained from azido-sydnone **39** by reaction with aldehydes in yields up to 81%. Optimization of the conditions revealed that an excess of aldehyde and the use of a nucleophilic phosphine such as PPhMe_2_ were necessary for the successful outcome of the process (Scheme 8). Subsequently, the reaction of sydnones **40** as dipoles with an aryne derivative generated from arene **41** in the presence of TBAF afforded the target indazolo[2,3-*a*]quinoxalines **42** under mild conditions and in high yields. In conclusion, Louis et al. [33] noted that the developed method may be useful for constructing small libraries of indazoloquinoxalines with interesting biological properties.

**Scheme 9:**
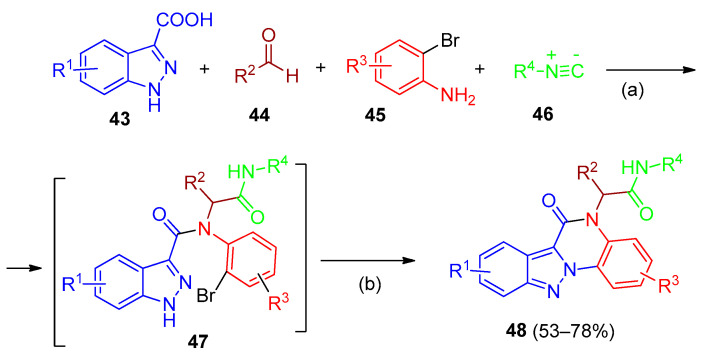
Synthesis of indazolo[2,3-*a*]quinoxalines **48**: (**a**) MeOH, room temperature, overnight; (**b**) K_2_CO_3_ (2 equiv.), CuI (10%), TMEDA (20%), DMF, MW, 150 °C, 30 min. **48**: R^1^ = H, Cl, Me; R^2^ = Ph, 4-ClC_6_H_4_, 4-MeC_6_H_4_, 4-BrC_6_H_4_, 4-MeOC_6_H_4_, 3-BrC_6_H_4_, H, Bz, thiophen-2-yl; R^3^ = H, Me, Cl, Br; R^4^ = *t*Bu, *n*Bu, 2,6-diMeC_6_H_3_, Bz, cyclohexyl. Color coding: blue—indazole fragment; red—aniline fragment; brown—aldehyde fragment; green—isocyanide fragment.

A straightforward method based on a multicomponent reaction (MCR) for the construction of indazolo[2,3-*a*]quinoxalines was developed [34]. Continuing their previous studies, Li et al. [34] proposed employing the Ugi reaction, a typical MCR, to generate an intermediate capable of undergoing intramolecular cyclization. As starting materials, they used 3-carboxyindazole **43a**, 4-chlorobenzaldehyde **44a**, 2-bromoaniline **45a**, and tert-butyl isocyanide **46a**. The mixture was kept in methanol at room temperature under air overnight (Scheme 9). Using the isolated product **47a** as a model, conditions for the subsequent Ullmann reaction were optimized. Under the established conditions—using K_2_CO_3_ as a base in the presence of CuI and TMEDA in DMF under microwave irradiation at 150 °C for 30 min—a series of indazolo[2,3-*a*]quinoxaline derivatives **48** was obtained via a one-pot procedure in yields up to 78%.

**Figure 7:**
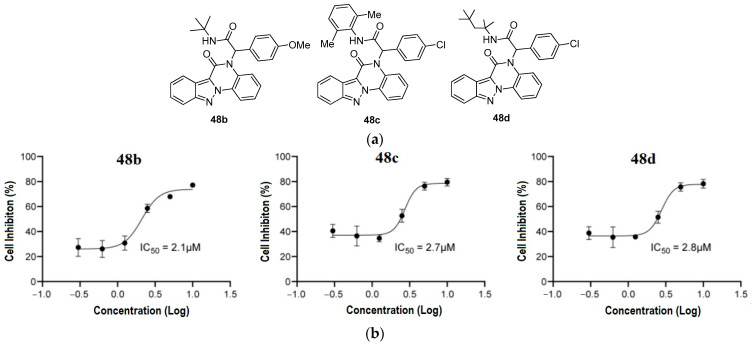
(**a**) Structure of compounds **48b–d**; (**b**) The half-maximal inhibitory concentration (IC_50_) of compounds **48b–d** in the HCT116 cell line. Reprinted from [34] under a CC BY license.

Some biological properties of products **48** were investigated. The antiproliferative effect of these compounds was studied on three human cancer cell lines: HCT116, MDA-MB-453, and Hep3B. Higher activity was observed for several compounds against colon cancer cells (HCT116). Three indazolo[2,3-*a*]quinoxaline analogs—**48b, 48c**, and **48d**—exhibited good anticancer activities, with IC_50_ values of 2.1 μM, 2.7 μM, and 2.8 μM, respectively (Figure 7); compound **48b** was the most potent among them.

Pyrazolo[1,5-*a*]quinoxalines have proven to be a flexible scaffold that hits a range of targets—TLR7, MAO, opioid receptors, and PI3Kα, to name a few. The standouts include **6a** and **6b** (TLR7 antagonists, IC_50_ ~8–10 μM) [21], **14c** (MAO-A inhibitor, IC_50_ = 0.028 μM) [25], **19c** and **19e** (opioid modulators with EC_50_ down to 1.47 μM for DOR) [11], and **28b** (PI3Kα inhibitor, IC_50_ = 0.24 μM, with fair isoform selectivity) [29]. The SAR picture is clear: *N*-acyl groups are best for MAO-A inhibition, while *N*-alkyl and *N*-allyl substituents work well for opioid antagonism. Adding amino groups to the quinoxalinone core improves PI3Kα binding via a hydrogen bond to Val851. That said, there are still some real gaps: most data come from in vitro work, with little in vivo PK or toxicity information; selectivity over related targets is often so-so; and the synthetic routes, though varied, have not been properly compared for scale-up. Going forward, the field needs more in vivo efficacy studies, better selectivity profiling against related kinases and receptors, and scalable, metal-free synthetic methods that can support real drug development.

## 3. Imidazo[1,2-a]quinoxalines

The general structure of imidazo[1,2-*a*]quinoxalines is shown in Figure 2. The review article [35] discussed the main achievements in the synthesis of imidazoquinoxalines. It was shown that approaches to imidazo[1,2-*a*]quinoxalines are based on either quinoxaline or imidazo derivatives. The methods for constructing the imidazoquinoxaline system are examined in detail according to the type of bond formed. Some biological properties of imidazo[1,2-*a*]quinoxalines are also presented.

**Scheme 10:**
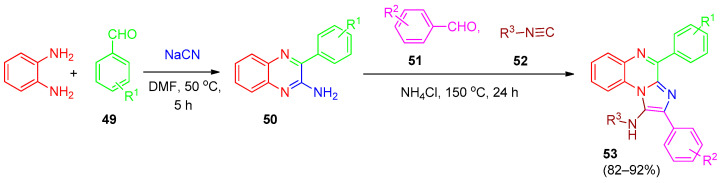
The synthetic approach to N-alkyl-2,4-diarylimidazo[1,2-*a*]quinoxalin-1-amine derivatives **53**. **53:** R^1^ = H, Cl, 3,4,5-(OMe)_3_; R^2^ = H, Cl, F, OMe, 3,4-(OMe)_2_, 3,4,5-(OMe)_3_; R^3^ = cyclohexyl, *t*-Bu. Color coding: red—ortho-phenylendiamine fragment, blue—cyanide fragment, green—aldehyde fragment in quinoxaline core, brown—isocyanide fragment; magenta—aldehyde fragment in imidazole core.

A simple and efficient route to new N-alkyl-2,4-diarylimidazo[1,2-*a*]quinoxalin-1-amine products **53** was proposed [36]. The synthesis was carried out in two steps. The reaction of *o*-phenylenediamine with aromatic aldehydes **49** in the presence of NaCN afforded 3-arylquinoxalin-2-amines **50**, which were then reacted with various substituted aldehydes **51** and isocyanides **52** to give the target products **53** (Scheme 10). Using the preparation of compound **50** from *o*-phenylenediamine and benzaldehyde with NaCN as a model, the conditions for the first step were optimized; the best results were obtained in DMF at 50 °C for 5 h, giving compound **50** in up to 85% yield. Optimization of the second step showed that using a **50**:**51**:**52**:NH_4_Cl molar ratio of 2:2:2:0.25 under solvent-free conditions at 150 °C for 24 h was optimal. Compounds **53** were characterized by a combination of spectroscopic techniques.

**Figure 8:**
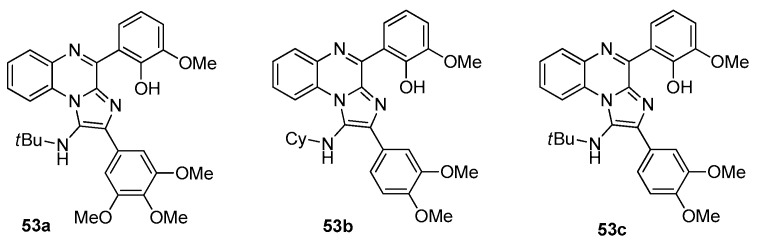
Structure of compounds **53a–c**.

The obtained imidazoquinoxalinones **53** were evaluated for their biological properties, including kinase inhibition and cytotoxic activity against several cancer cell lines. The cytotoxic effect (% viability) was studied on three cell lines—HEK-293, MB-468, and CCRF—after 24 and 72 h of treatment at a concentration of 50 μM. The results showed that analogs **53a–c** (Figure 8) were active against all three cancer cell lines. For these three compounds, cytotoxicity assays were further performed on human B-cell leukemia lines (BV-173, K-562), as well as on MCF 10A and MDA-MB-468 cells. They demonstrated anti-proliferative activities with IC_50_ values of 9.77 μM (**53a**), 12.02 μM (**53b**), and 15.84 μM (**53c**) against K-562 cells. Against the other cell lines, IC_50_ values exceeded 25 μM [36].

Compounds **53a–c** were examined as kinase inhibitors against three kinases: ABL1, c-Src, and LCK. Compound **53c** showed inhibitory activity against ABL1 and c-Src, with IC_50_ values of approximately 5.25 and 3.93 μM, respectively. Taken together, the results of the cell culture and enzyme assay studies identified compound **53c** as the most active compound in this series [36].

**Scheme 11:**
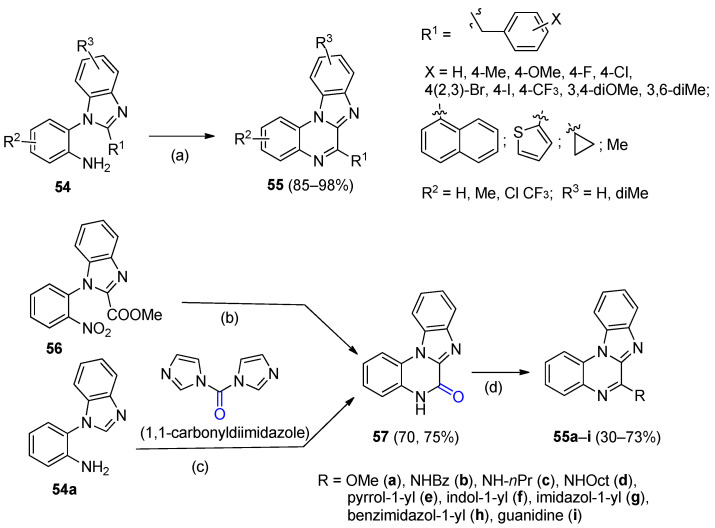
Synthesis of benz[4,5]imidazo[1,2-*a*]quinoxalines **55.** Reagents and conditions: (**a**) I_2_, NaOAc, toluene, reflux; (**b**) NH_4_Cl, Zn, 0 °C, 30 min; (**c**) *o*-chlorobenzene, 140 °C, 4 h; (**d**) POCl_3_, then NuH. No color coding is used as one reagent contains all atoms of the polycyclic core.

An approach to the synthesis of new benz[4,5]imidazo[1,2-*a*]quinoxalines **55** (Scheme 11), based on an I_2_-mediated direct sp^3^ C–H amination, was described [37]. The starting materials were 2-(benzimidazol-1-yl)anilines **54**, which are readily prepared by the reaction of fluoronitrobenzenes with benzimidazoles [37]. The reaction conditions were optimized, and it was found that carrying out the process with a **54**:I_2_:NaOAc molar ratio of 0.5:1.1:2.5 in refluxing toluene afforded the target products **55** in high yields. However, when R^1^ was the ethyl or (cyclopropyl)methyl group, the yields dropped to 44% and 24%, respectively. Target products **55** were characterized by various spectroscopic methods, including single-crystal X-ray diffraction analysis for **55** (R^1^ = 4-CF_3_C_6_H_4_, R^2^, R^3^ = H). Chen et al. [37] discuss a plausible reaction mechanism. The developed method is straightforward to perform, does not require transition metals, is scalable, and provides heterocyclic compounds useful for medicinal chemistry.

A facile synthesis of C6-substituted benz[4,5]imidazo[1,2-*a*]quinoxaline analogs **55** (Scheme 11) and an investigation of their anticancer properties were presented [38]. Benzimidazoquinoxalinones **57** were obtained via intramolecular cyclization of *o*-nitro(amino)phenylbenzimidazoles **56**. Treatment of scaffolds **57** with a mixture of POCl_3_ and DMF (Scheme 11) at 110 °C, followed by reaction of the resulting 6-chlorobenzimidazoquinoxaline with various nucleophiles, afforded desired analogues **55** in yields up to 73%.

Compounds **55** were evaluated for anticancer activity against the NCI-60 cancer cell lines. The two-stage screening process began at a single dose of 10.0 μM, with cisplatin used as a positive control. It was found that the substituent R in compounds **55** had a significant influence on activity. Higher activity was observed for derivatives bearing a heterocyclic R group against the MDA-MB-231, MDA-MB-468, and MCF7 cancer cell lines (Table 5). Compounds **55f–h** showed considerable activity against the triple-negative breast cancer cell line MDA-MB-468 (IC_50_ = 3.2–17.28 μM) as well as against another breast cancer cell line, MCF-7 (IC_50_ = 2.59–18.77 μM). Importantly, these compounds demonstrated significantly lower toxicity toward normal cell lines: the IC_50_ values against MCF12A (normal breast epithelial cells) were 32–44 μM, corresponding to 5- to 12-fold selectivity over cancer cells (Table 5). In contrast, compound **55i** (bearing a guanidine group) showed potent anticancer activity but was also toxic to normal cells (IC_50_ = 1.04 μM against MCF12A), indicating poor selectivity [38].

**Scheme 12:**
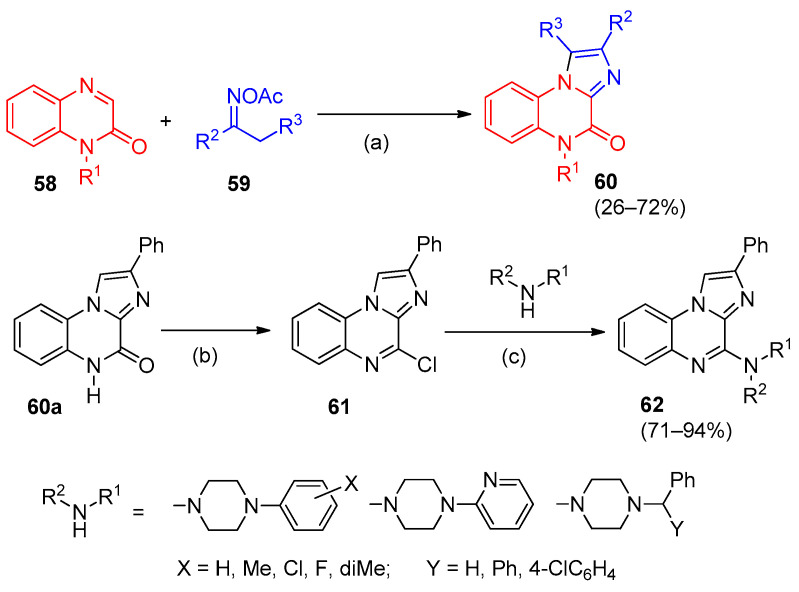
Synthesis of imidazo[1,2-*a*]quinoxaline-4-ones **60**, imidazo[1,2-*a*]quinoxalines **62**: (**a**) I_2_, DMSO, 100 °C, 4–6 h; (**b**) POCl_3_, 130 °C, 0.5–1 h; (**c**) K_3_PO_4_, H_2_O, 100 °C, 4 h. **60**: R^1^ = H, Me, Et, Bz, CH_2_COOEt; R^2^ = Ph, 4-MeC_6_H_4_, 4-MeOC_6_H_4_, 4-FC_6_H_4_, 2-ClC_6_H_4_, 4-CF_3_C_6_H_4_, naphthalen-1-yl, 2,3-dihydrobenzo[*b*][1,4]dioxin-6-yl; R^3^ = H, Ph. Color coding: blue—imidazole fragment; red—quinoxaline core.

A novel and efficient method for the synthesis of imidazo[1,2-*a*]quinoxalin-4-ones **60** via I_2_-mediated oxidative [3+2] annulation of quinoxalinones **58** with oxime esters **59** (Scheme 12) was reported [39]. The starting substrates are readily available, and the reaction is straightforward, yet it affords the target products **60** with high regioselectivity. Intermediate **60a** was functionalized to generate a series of amino-substituted imidazo[1,2-*a*]quinoxalines **62** for further biological studies. Compounds **60** and **62** were characterized by ^1^H and ^13^C NMR spectroscopy, and analog **60** (R^1^ = Et, R^2^ = Ph, R^3^ = H) was additionally confirmed by X-ray diffraction data.

A molybdenum-catalyzed one-pot synthesis of benzimidazo[1,2-*a*]quinoxalines **65** based on the reaction of nitroarenes with a glycol was developed [40]. In this process, the glycol serves as a reducing agent, and the transformation proceeds as a Mo-catalyzed domino reaction involving reduction of the nitro group of 1-(2-nitrophenyl)benzimidazole **63** to an amino group, oxidation of the glycol **64** to a carbonyl compound, formation of an imine intermediate, and its subsequent cyclization to the target product **65**. The method is highly efficient and atom-economical, allowing the amount of external reducing agent to be reduced. Gymez-Gil et al. [40] demonstrated its potential for the synthesis of other annulated heterocycles as well.

**Scheme 13:**
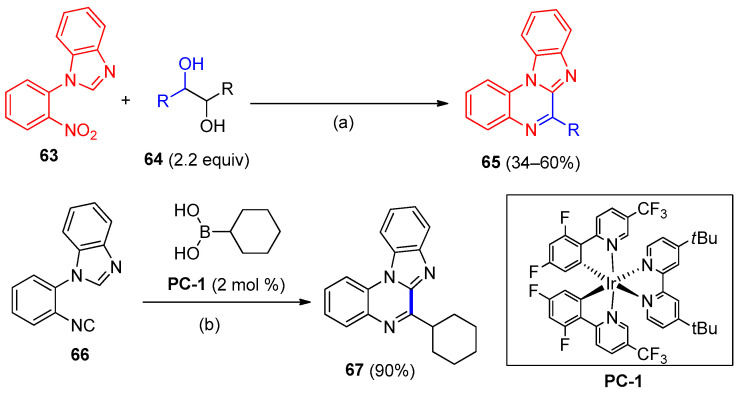
The synthetic approaches to benzimidazo[1,2-*a*]quinoxalines **65**, **67**: (**a**) MoO_2_Cl_2_(dmf)_2_ (5 mol%), *p*-TsOH (1.5 equiv.), DMA (0.5 M), MW, 200 °C, 30 min; (**b**) DBU, 1,4-dioxane, Blue LED (30 W), 9 h. **65**: R = Ph, 4-MeC_6_H_4_, 4-BrC_6_H_4_, 4-FC_6_H_4_, 2-ClC_6_H_4_. Color coding: blue—carbonyl fragment; red—1-(2-nitrophenyl)benzimidazole fragment.

The synthesis of 6-cyclohexylbenzimidazo[1,2-*a*]quinoxaline **67** was accomplished using a photoredox-catalyzed method (Scheme 13) developed in a recent study [41]. An isocyanide **66** and cyclohexylboronic acid were employed as starting reagents. The reaction proceeds under photocatalytic conditions upon irradiation and involves the generation of alkyl radicals followed by cyclization. This method is also applicable to the synthesis of a broad range of annulated heterocycles.

**Scheme 14:**
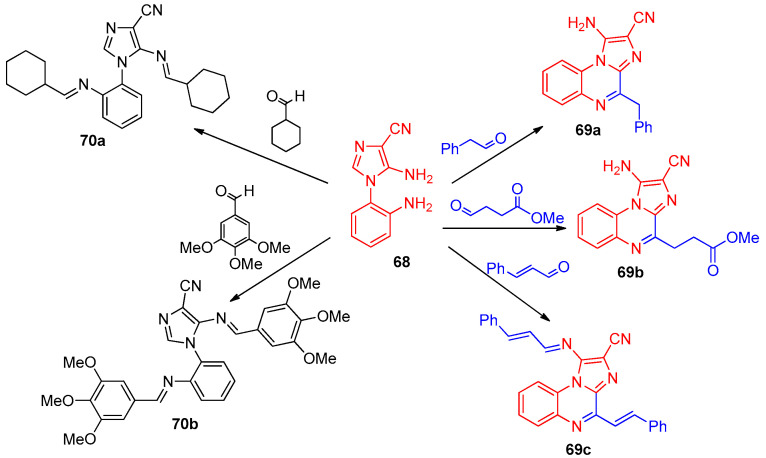
Synthesis of compounds **69a–c**, **70a,b**. Color coding: blue—carbonyl fragment; red—2-imidazolylaniline fragment.

Goel and co-workers investigated imidazo[1,2-*a*]quinoxaline analogs as antitubulin agents with anticancer activity using various approaches [42,43]. Imidazo[1,2-*a*]quinoxalines **69a–c** and acyclic compounds **70a,b** were synthesized by reacting 2-imidazolylaniline **68** with appropriate reactants in the presence of *p*-TsOH according to reported procedures (Scheme 14) [42]. The cytotoxicity of compounds **69a–c** was evaluated against four cancer cell lines (MCF-7, MDA-MB-231, A549, HCT-116). Compound **69a** showed the best activity against all four lines (Table 6). The acyclic analog **70b** also exhibited cytotoxicity against these cells comparable to that of colchicine, which was used as the standard drug for this series. Both derivatives also proved to be excellent antioxidants.

The SAR analysis across the imidazo[1,2-*a*]quinoxaline series **69**, **70** reveals several key trends: ring fusion (cyclization) consistently enhances potency compared to acyclic analogs; 3,4,5-trimethoxyphenyl and 3,4-dimethoxyphenyl substituents are optimal for tubulin binding, mimicking the colchicine pharmacophore; free amino groups at position 1 contribute to hydrogen bonding with Val238 and Lys352; and allyl or propargyl substituents on the imidazole nitrogen led to reduced cytotoxicity. These findings provide a clear rationale for the continued exploration of imidazoquinoxalines as tubulin-targeting anticancer agents.

Compounds **69a** and **70b**, being the most active ones, were selected for tubulin polymerization assays. They exhibited a profile similar to that of colchicine relative to the control and demonstrated the ability to inhibit tubulin polymerization in a cell-free system. Molecular docking of **69a** and **70b** against tubulin protein co-crystallized with colchicine revealed that both derivatives showed better binding than the standard tubulin-binding agent colchicine [42].

In a subsequent study by the same team [43], a library of 34 imidazo[1,2-*a*]quinoxalines was selected using computational methods (virtual screening, molecular docking, and molecular mechanics) and investigated for tubulin inhibition with antitumor activity.

**Figure 9:**
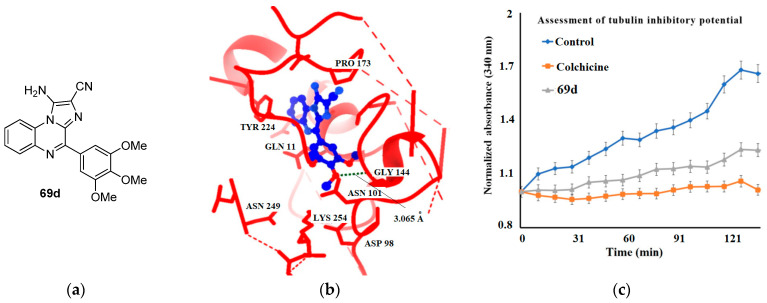
(**a**) Structure of compound **69d**; (**b**) Illustration portraying the interaction pattern of **69d** (3D surface model) within the colchicine-binding domain; (**c**) Graph suggesting the tubulin inhibitory potential of **69d** compound with colchicine. Reprinted from [43] under a CC BY license.

Importantly, the computational predictions were rigorously validated through experimental in vitro assays. The docking analysis (Figure 9b) identified key interactions between compound **69d** and the colchicine-binding pocket of tubulin, including hydrogen bonding with Lys352 and hydrophobic contacts with Cys241, Leu248, Leu255, Val318, Ala316, and Met259 (docking score: −11.45 kcal/mol, comparable to colchicine at −9.15 kcal/mol) [43]. These in silico findings were subsequently confirmed by tubulin polymerization inhibition assays, which demonstrated that **69d** effectively destabilizes microtubule formation in a cell-free system, albeit with slightly lower potency than colchicine. Furthermore, in vitro antiproliferative assays against MCF-7, MDA-MB-231, HCT-116, and A549 cell lines yielded IC_50_ values of 4.33–6.11 μM, which are comparable to those of colchicine (5.11–6.55 μM), thereby corroborating the docking predictions (Table 6). The consistency between the computational binding mode and the experimental biological data underscores the reliability of the in silico approach for identifying potential tubulin-targeting anticancer agents and validating compound **69d** as a promising lead for further optimization.

In their concluding remarks, Goel et al. [43] noted that the successful application of both in silico and in vitro approaches enables the identification of a potential lead compound within a series of heterocyclic derivatives.

**Figure 10:**
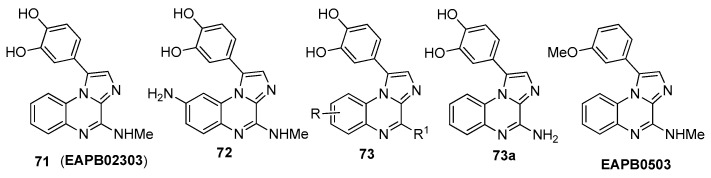
Structure of compounds **71**–**73**, **73a**, **EAPB0503**. **73**: R = H, 7-CF_3_, 7-OCF_3_, 8-CF_3_; R^1^ = NH_2_, OH, NHMe, NMe_2_, ethyl-1,2-diamino, propyl-1,3-diamino, hexyl-1,6-diamino, piperazine.

Patinote and colleagues, continuing their research on imidazo[1,2-*a*]quinoxaline analogs, reported the synthesis of new compounds as well as further studies on previously prepared ones [44,45]. Products **71** and **72** [44] (Figure 10) were obtained according to published procedures [46,47] and exhibited significant cytotoxic activity against the human melanoma cell line A375. Compound **71**, recognized as a second-generation lead in the imidazo[1,2-*a*]quinoxaline series, displayed an original mechanism of action distinct from that of other compounds. Owing to their planar, rigid π-conjugated systems, both derivatives behave as fluorescent chromophores, which allowed Patinote et al. [44] to investigate them using confocal microscopy to study cellular penetration and localization in melanoma A375 cells. These studies showed that imidazo[1,2-*a*]quinoxaline **72**, which possesses more favorable luminescent properties for determining subcellular distribution, was predominantly taken up into vesicles without specific accumulation in organelles or the nucleus.

In subsequent work, a new series of 1-(3,4-dihydroxyphenyl)imidazo[1,2-*a*] quinoxalines **73** (Figure 10) bearing variations in substituents R and R^1^ at the quinoxaline core was synthesized, and in vitro investigations were carried out [45]. The cellular activity of derivatives **73** (eleven compounds) was evaluated to study their efficacy on the proliferation of the A375 human melanoma cell line. Compounds **71** (**EAPB02303**) and **73a** were the most active in this series, showing IC_50_ values of 3 and 60 nM, respectively. For the best compound, **EAPB02303**, in vitro tubulin polymerization and activity against different melanoma cell lines (A375, ME WO, A2058, IPC 298) were studied in comparison with the first-generation imidazoquinoxaline lead (**EAPB0503**) and vemurafenib, which is used for BRAF mutations. It was found that, unlike **EAPB0503**, **EAPB02303** lacks the ability to inhibit tubulin polymerization [45].

The data obtained from experiments with other melanoma cell lines indicates that, whereas vemurafenib selectively inhibits BRAF V600E mutant melanoma cell lines, **EAPB02303** can suppress the growth of all melanoma cell lines tested (Table 7). Based on these findings, Patinote et al. [45] selected only the lead compound **EAPB02303** for further mechanistic studies.

To this aim, an experiment was conducted involving **EAPB02303**, **EAPB0503**, and twelve additional drugs representative of the diversity of anticancer mechanisms of action. A375 cells were treated for six hours with each compound at a concentration of 10 μM (DMSO served as the negative control). Comparison of the results showed that the tested agents could be classified into four groups according to their mechanism: tubulin inhibitors, topoisomerase inhibitors, alkylating agents, and antimetabolic agents. However, **EAPB02303** did not associate with any of these categories, indicating a distinct mechanism of action and showing pronounced in vivo antitumor activity, decreasing both tumor size and weight in a human melanoma xenograft model in a dose-dependent fashion, which was associated with a reduced mitotic index rather than necrosis [45].

**Figure 11:**
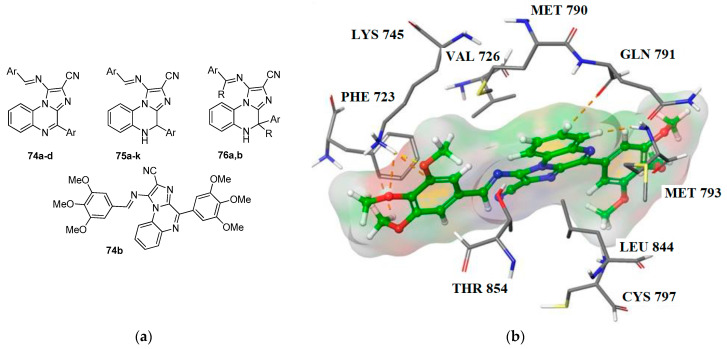
(**a**) Structure of compounds **74**–**76**; (**b**) 3D docking pose of **74b** at the active site of EGFR. Reprinted from [48] under a CC BY license. **74**: Ar = 2,5-dimethoxyphenyl (**a**), 3,4,5-trimethoxyphenyl (**b**), 3,4-dimethoxyphenyl (**c**), 4-chlorophenyl (**d**); **75**: Ar = 3,4-dimethoxyphenyl (**a**), 2-nitrophenyl (**b**), 3,4-dihydroxyphenyl (**c**), 2,5-dimethoxyphenyl (**d**), 2-bromophenyl (**e**), 2,4-dinitrophenyl (**g**), 2-fluorophenyl (**h**), 3,4,5-trimethoxyphenyl (**i**), 4-nitrophenyl (**j**), 4-cyanophenyl (**k**); **76**: 4-chlorophenyl (**a**), 3,4-dimethoxyphenyl (**b**).

Kumar and co-workers, in a series of studies [48,49,50], presented new non-covalent imidazo[1,2-*a*]quinoxalines **74**–**76** (Figure 11) as inhibitors of the Epidermal Growth Factor Receptor (EGFR) along with their anticancer evaluation. These compounds were prepared by reacting 2-imidazolylaniline **68** with the appropriate aldehydes or ketones in methanol at 80 °C in the presence of *p*-TsOH under microwave irradiation for 25–30 min, giving yields of up to 93% (analogous to Scheme 14). All derivatives were characterized by NMR, HRMS, and IR spectroscopy.

Compounds **74**–**76** were first evaluated in vitro for their ability to inhibit EGFRWT by blocking ATP-dependent phosphorylation of EGFR. Only selected compounds (**74b, 75h**, **75j**, **76a**, and **76b**) inhibited EGFRWT with IC_50_ values of 211.22, 222.21, 193.18, 223.32, and 221.53 nM, respectively, which were comparable to that of erlotinib (221.03 nM). The remaining derivatives showed very weak or no activity. The selected compounds were further analyzed for their antiproliferative potential against EGFRWT-expressing cancer cell lines (A549, HCT-116, and MDA-MB-231) using an MTT assay (Table 8). It was found that several of the tested compounds exhibited higher antiproliferative activity than erlotinib (positive control) against two of the cancer cell lines. Moreover, they were not cytotoxic toward normal cells (HBL-100 and HPBMCs) at the maximum tested concentration of 10 μM after 24 h of incubation [48].

Furthermore, these five substances were also evaluated against a gefitinib-resistant NSCLC cell line, H1975. Compounds **74b**, **75j**, and **76a** showed significant growth inhibitory activity, with IC_50_ values of 3.65, 8.53, and 5.0 μM, respectively, meaning they were far more potent against H1975 than gefitinib (IC_50_ > 20 μM). Notably, **74b** may represent a lead compound for the treatment of gefitinib-resistant EGFR-mutant NSCLC. For this compound, molecular docking was performed to assess possible interactions at the active site of EGFRL858R/T790M (Figure 11b). The results provided a rationale for the EGFR inhibitory activity of **74b**, as it was able to interact with both the wild-type residue Thr790 and the mutant Met790, as well as with Lys745 at a shorter distance in the mutant type [48].

In a subsequent study, Bhat et al. [49] investigated the antitumor activity of **74b** in vivo using a xenograft model of NSCLC induced by transplanting A549 cells into nude mice. This compound was found to reduce tumor volume, particularly at a high dose of 30 mg/kg, while the percentage change in body weight was minimal. Moreover, **74b** improved mouse survival. The compound also showed good stability toward human and mouse liver microsomes. Overall, the in vivo results corroborated the in vitro findings, leading the authors [49] to propose that compound **74b** holds potential antitumor activity against EGFR-dependent lung cancer.

**Figure 12:**
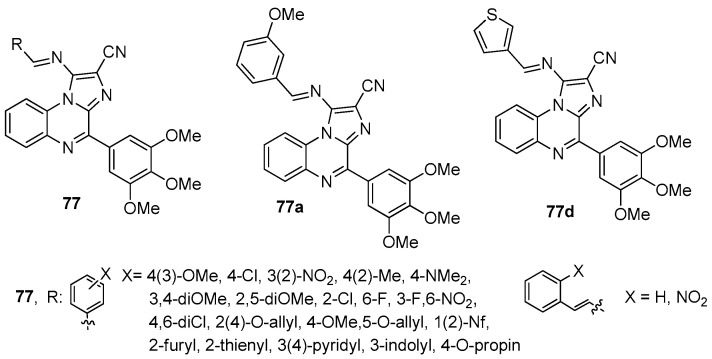
Structure of compounds **77**, **77a**, **77d**.

In a further study, Kumar et al. [50] optimized the lead compound **74b** with the aim of improving its antitumor activity based on the EGFR catalytic site. They synthesized 29 new analogs of **74b** differing in the substituent at the imino group (compounds **77**, Figure 12), which were characterized using a combination of spectroscopic methods.

All derivatives **77** were tested for their antiproliferative activity against lung cancer cells and breast cancer cells with low and high metastatic activity using the MTT assay, with erlotinib as a positive control. Most of them demonstrated pronounced antiproliferative properties. Structure–activity relationship analysis allowed the identification of the most important structural features governing anticancer activity. After evaluating the cytotoxic potential, the authors [50] selected a group of compounds **77a–e** (Table 9) that showed no more than 5% toxicity toward normal cells at the maximum concentration of 25 μM. Subsequently, compounds **77a–e** were used in an EGFR-mediated phosphorylation inhibition assay. It was shown that at a concentration of 200 nM, only **77a** and **77d** exhibited more than 50% inhibition compared to the positive control erlotinib, displaying IC_50_ values of 75.91 and 84.3 nM, respectively, versus 135.29 nM for erlotinib. Molecular modeling studies of compounds **77a** and **77d** further supported their EGFR inhibitory activity [50].

Further investigation of compounds **77a** and **77d** revealed that they reduced the mRNA expression levels of EGFR, KRAS, and MAP2K genes. Notably, product **77d** led to a more pronounced decrease in EGFR and KRAS gene expression compared to erlotinib. In addition, compounds **77a** and **77d** lowered oxidative stress and influenced mitochondrial activity at higher doses relative to A549 cells treated with erlotinib. Compounds **77a** and **77d** were then further assessed for their anticancer potential against lung cancer. The in vivo studies of **77a** and **77d** are underway, and results will be disclosed in due course [50].

**Figure 13:**
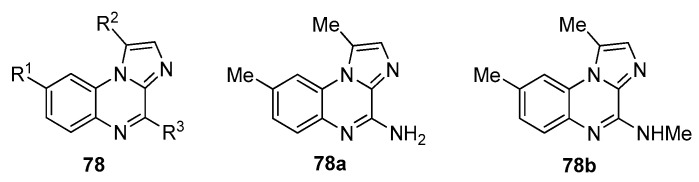
Structure of compounds **78**. R^1^ = H, Me; R^2^ = H, Me, Cl, Br; R^3^ = Cl, OR, NH_2_, NHR, NHSO_3_R, NHNH_2_, NHNHCOR.

In a separate study, Ma et al. [51] reported a possible application of imidazo[1,2-*a*]quinoxalines as novel fungicides in agriculture. They synthesized a broad series of compounds **78** (Figure 13) from readily available and inexpensive reagents and evaluated their antifungal activity against ten typical phytopathogenic fungi. The in vitro results showed that some compounds exhibited more potent broad-spectrum fungicidal activity than two commercially available fungicides, chlorothalonil and hymexazol. It was also found that *Valsa mali* and *Botrytis cinerea* strains were particularly sensitive to ten analogs of **78**, with IC_50_ values ranging from 1.4 to 27.0 μg/mL. The highest inhibitory effects were demonstrated by compound **78a** against *Valsa mali* (EC_50_ = 5.6 μg/mL) and compound **78b** against *Fusarium solani* (EC_50_ = 5.1 μg/mL).

Preliminary results regarding the mechanism of action suggested that the antifungal effect of compounds **78** is associated with the disruption of spore germination and germ tube growth. Importantly, cellular experiments showed that these compounds have good safety toward BV2 cells [51].

**Scheme 15:**
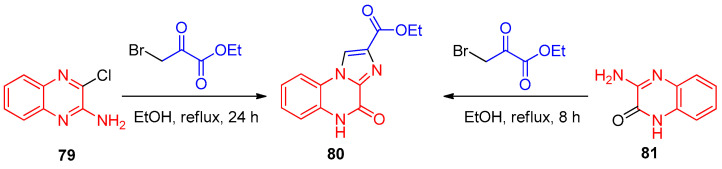
Synthesis of compound **80**. Color coding: blue—ketoester fragment; red—aminoquinoxaline core.

**Figure 14:**
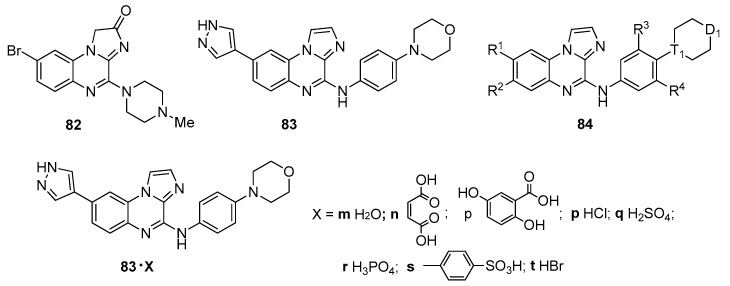
Structure of compounds **82**–**84**, **83‧X**. **84**: R^1^, R^2^ = H or pyrazolyl; R^3^, R^4^ = H, alkyl, alkoxyl, halo, amino, etc.; T = CH or N; D = O, C(R), N(R), etc.

Data on the synthesis and biological activity of imidazo[1,2-*a*]quinoxalines have also been reported in several patents. For instance, the preparation of ethyl 4-oxo-4,5-dihydroimidazo[1,2-*a*]quinoxaline-2-carboxylate **80** by reacting 3-chloroquinoxalin-2-amine **79** or 3-aminoquinoxalin-2-one **81** with ethyl 3-bromo-2-oxopropionate under reflux in ethanol is described in patent [52] (Scheme 15). The advantages of this method include high yield, high product purity, low cost, and simple operation. Compound **82** is reported in patent [53] as an H4 histamine receptor inhibitor (79.7% inhibition at 1 μM) (Figure 14).

Chen and co-workers, in several patents [54,55,56,57], describe imidazo[1,2-*a*]quinoxaline derivatives as spleen tyrosine kinase (Syk) and vascular endothelial growth factor receptor 2 (VEGFR2) dual-target inhibitors. For example, compound **83** (Figure 14) was obtained via a multi-step synthetic route starting from 1-bromo-3-fluoro-4-nitrobenzene and 1*H*-imidazole [54]. Target products **84**, synthesized by the reaction of 8-bromo-N-(4-morpholinophenyl)imidazo[1,2-*a*]quinoxalin-4-amine with 1*H*-pyrazole-4-boronic acid pinacol ester in the presence of a palladium catalyst system, represent an extended series of analogs of compound **83** [55,56]. These compounds are used in the preparation of medicaments for the prevention or treatment of Syk- and VEGFR2-mediated diseases, including xerophthalmia, cancer, adult respiratory distress syndrome, asthma, and others. Patent [57] presents salt and crystalline forms of compound **83** (**83 X**), their preparation methods, and their use in the manufacture of drugs related to dual Syk and VEGFR2 inhibitors.

Imidazo[1,2-*a*]quinoxalines are the most heavily studied subclass of azoloquinoxalines. They have shown activity as kinase inhibitors (EGFR, ABL1, c-Src), tubulin polymerization inhibitors, and antifungal agents. The most impressive examples include **74b** (EGFR inhibitor, IC_50_ = 211 nM; antiproliferative IC_50_ = 2.7 nM against A549 cells) [48], **69a** and **69d** (tubulin inhibitors with activity on par with colchicine, IC_50_ ~4–5 μM) [43], **EAPB02303** (compound **71**, melanoma cells, IC_50_ = 3 nM) [45], and **78a** (antifungal, EC_50_ = 5.6 μg/mL against *Valsa mali*) [51]. SAR work points to 3,4,5-trimethoxyphenyl and 3,4-dimethoxyphenyl as the best picks for EGFR inhibition, while 3,4-dihydroxyphenyl groups boost tubulin binding and anti-melanoma effects. On a promising note, **EAPB02303** has shown in vivo activity in a human melanoma xenograft model—a real step toward the clinic. Still, there are some clear shortcomings: selectivity over related kinase isoforms is only modest; data on metabolic stability and oral bioavailability are thin; many syntheses rely on microwave or metal-catalyzed steps that could complicate scale-up; and despite all the promising preclinical data, there is still no clinical-stage candidate. Moving forward, the priority should be better pharmacokinetics, broader in vivo testing across different tumor models, and greener, more scalable synthetic routes.

## 4. Imidazo[1,5-a]quinoxalines

The general structure of imidazo[1,5-*a*]quinoxalines is shown in Figure 2. The review article [35] discussed the main achievements in the synthesis of two types of imidazoquinoxalines, one of which is imidazo[1,5-*a*]quinoxalines. Approaches to the synthesis of this class based on imidazole and quinoxaline derivatives, as well as several other methods, were examined. The biological activity of imidazo[1,5-*a*]quinoxalines was also briefly covered. In subsequent years, articles have been published that complement the main approaches or propose new, original ones.

**Scheme 16:**
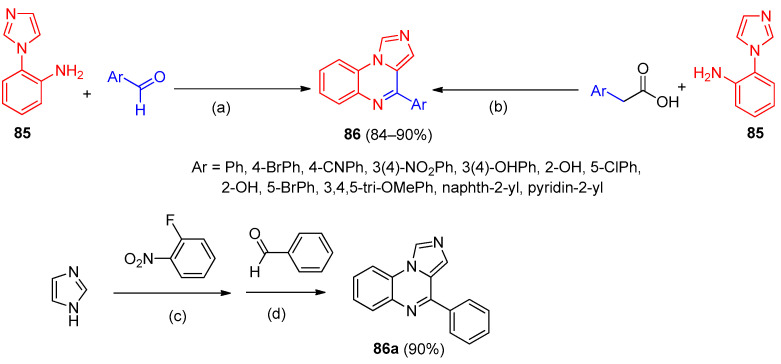
Synthesis of compounds **86**: (**a**) Wang-OSO_3_H, toluene, 110 °C, 5 h; (**b**) I_2_ (20 mol%), DMSO, 100–110 °C, 5–7 h; (**c**) KOH/D-glucose, DMSO/H_2_O; (**d**) acetic acid. Color coding: blue—arylderivative fragment; red—2-(1H-imidazol-1-yl)aniline fragment.

An efficient approach to the synthesis of imidazo[1,5-*a*]quinoxalines **86** bearing various substituents from 2-(1*H*-imidazol-1-yl)aniline **85** and different aldehydes using Wang-OSO_3_H as a reusable catalyst to achieve high yields was reported [58]. The reaction was carried out in toluene under reflux for 5 h, affording the target products in 84–90% yield (Scheme 16). Later, the same team described, using imidazo[1,5-*a*]quinoxaline **86a** as an example, a scalable one-pot multicomponent process [59] employing imidazole, an aryl aldehyde, and 2-fluoronitrobenzene as starting reagents, along with *D*-glucose as a clean, renewable, and eco-friendly reductant in an alkaline aqueous medium (Scheme 16). The process involves three steps: fluorine substitution by the imidazole ring, reduction of the nitro group to an amino group, and cyclization of the resulting 2-(1*H*-imidazol-1-yl)aniline with benzaldehyde.

In a subsequent study by the same team [60], a new methodology for the construction of imidazo[1,5-*a*]quinoxalines **86** was developed. Instead of aldehydes, the use of arylacetic acids with a molecular iodine-DMSO green catalytic system was proposed (Scheme 16). Control experiments allowed Chiranjeevi et al. [60] to confirm the proposed mechanism, in which the process begins with oxidation of the arylacetic acid by the I_2_-DMSO system followed by decarboxylation to generate an aryl aldehyde, which then undergoes substitution, cyclization, and oxidation to afford **86**. The yields of derivatives **86** under these conditions ranged from 88% to 91%. This methodology offers several advantages and enables the production of imidazo[1,5-*a*]quinoxaline libraries useful for medicinal chemistry.

**Scheme 17:**
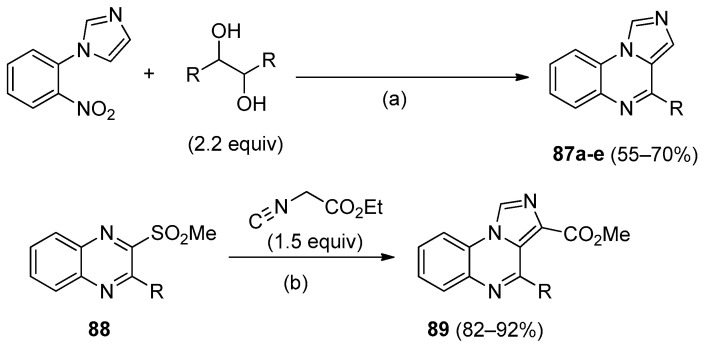
Synthesis of compounds **87**, **89**: (**a**) MoO_2_Cl_2_ (dmf)_2_ (5 mol%), p-TsOH (50 mol%), DMA (0.5 M), MW, 180 °C, 30 min; (**b**) DBU (4.5 equiv.), DMF, room t., 2–3 h. **87**: R = Ph (**a**), 4-MeOC_6_H_4_ (**b**), 4-FC_6_H_4_ (**c**), furan-2-yl (**d**), *n*-Bu (**e**); **88**, **89**: R = Ph, 4-MeC_6_H_4_, 4(3)-MeOC_6_H_4_, 4,3-diMeOC_6_H_3_, 3,5-diMeOC_6_H_3_, 4(3)-ClC_6_H_4_, 4-BrC_6_H_4_, 4-NO_2_C_6_H_4_, 4-PhC_6_H_4_, thiophen-2-yl. No color coding is used, as this scheme illustrates two different processes for the formation of the polycyclic core.

The molybdenum-catalyzed one-pot synthesis of N-polyheterocycles from nitroarenes and glycols was developed and applied not only to the preparation of benzimidazo[1,2-*a*]quinoxalines **65** (see Scheme 13) but also to imidazo[1,5-*a*]quinoxalines **87a–e** (Scheme 17) [40]. The reaction was carried out in the presence of *p*-TsOH (50 mol%) under microwave irradiation at 180 °C, with the glycol serving as a reducing agent. It is worth noting that in this case, a heteroaromatic glycol (for **87d**) and a mixed secondary-tertiary diol (for **87e**) were also tested as reducing agents.

A new route to 4-R-imidazo[1,5-*a*]quinoxaline-3-carboxylates **89** was described [61] (Scheme 17). 2-(Methylsulfonyl)-3-phenylquinoxaline (**88a**), a quinoxaline bearing a good leaving group, and ethyl isocyanoacetate (a reagent for imidazole ring construction) were chosen as starting materials, and the optimal conditions were established. The best conditions for the cycloaddition reaction were found to be DMF as solvent, an excess of DBU as a base, room temperature, and a reaction time of 2–3 h. The target products were obtained in high yields. The method is efficient and convenient, allowing variation in the substituent on the quinoxaline ring for library construction.

**Scheme 18:**
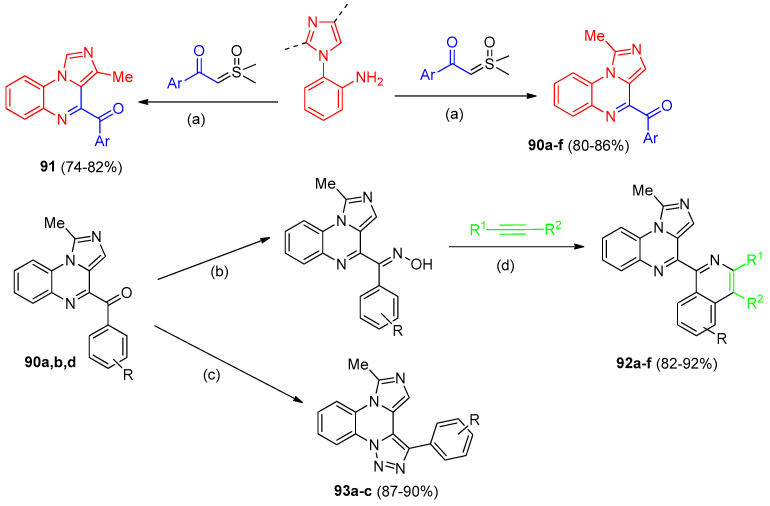
Synthesis of compounds **90**, **91**: (**a**) S_8_ (60 mol%), DMSO, air, 100 °C; (**b**) NH_2_OH, EtOH:H_2_O (2:1), reflux, 3–4 h; (**c**) AcCl, EtOH, then TsNHNH_2_, then EtOH, reflux, 6–24 h; (**d**) [Ru(*p*-cymene)_2_Cl_2_]_2_, KPF_6_, MeOH, 60 °C, 24 h. **90**: **90**. Ar = Ph (**a**), 4-MeC_6_H_4_ (**b**), 4-CF_3_C_6_H_4_ (**c**), 2,4-diClC_6_H_3_ (**d**), thiophen-2-yl (**e**), cyclopropyl (**f**); **91**: Ar = 4-MeC_6_H_4_ (**a**), OCH_2_Ph (**b**), 4-BrC_6_H_4_ (**c**), 3-Cl-diPh (**d**); **92**: R = H, R^1^ = R^2^ = Ph (**a**); R^1^ = R^2^ = Et (**b**); R^1^ = Me, R^2^ = Ph (**c**); R^1^ = Ph, R^2^ = Ph-Ethyn(**d**); R = 4-Me, R^1^ = R^2^ = Ph (**e**); R^1^ = Me, R^2^ = Ph (**f**); **93**: R = H (**a**), 4-Me (**b**), 2,4-diCl (**c**). Color coding: blue—arylcarbonyl fragment; red—2-(1*H*-imidazol-1-yl)aniline fragment; green—alkyne fragment.

A metal-free approach to the synthesis of 4-ArC(O)-imidazo[1,5-*a*]quinoxalines **90** and **91** starting from 2-(4)-(methylimidazolyl)aniline and a β-ketosulfoxonium ylide as a C1 synthon for cyclization was presented [62]. The process proceeds as a [5+1] cascade cyclization in the presence of elemental sulfur as a catalyst, affording the target products in high yields under specific optimized conditions (Scheme 18). Compounds **90** and **91** were characterized by various spectroscopic methods, including single-crystal X-ray diffraction analysis for target product **90f**. Based on literature precedents and their own experimental data, Ghosh et al. [62] proposed a plausible reaction mechanism.

The presence of a carbonyl group in compounds **90** and **91** allowed the authors to carry out further modifications to construct π-extended N-heterocycles. Thus, by reacting compounds **90a,b** with hydroxylamine followed by alkynes, a series of isoquinoline-substituted imidazo[1,5-*a*]quinoxalines **92a–f** were synthesized, representing hybrids in which the heterocycles are linked by a single Csp^2^–Csp^2^ bond (Scheme 18). Treatment of derivatives **90a,b,d** with an acyl chloride in ethanol followed by reaction with tosylhydrazide under reflux afforded [1,2,3]triazoloheteroarenes **93a–c** (Scheme 18) [62].

**Figure 15:**
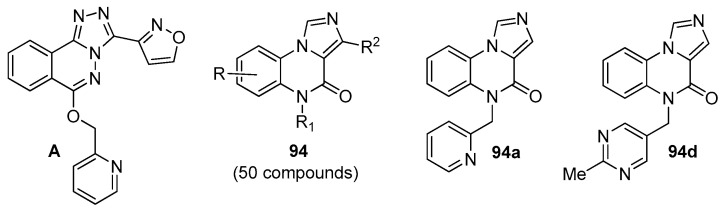
Structure of compounds **A**, **94**, **94a**, **94d**. **94**: R = H, Cl, Br, F, Me, CN, OMe, OCF_3_, CMe_3_; R^1^ = Et, (pyridin-2-yl), (pyridin-2-yl)-methyl, (pyridin-3-yl)-methyl, (pyridin-4-yl)-methyl, (pyrimidin-5-yl)-methyl; R^2^ = H, Me, Et, Cl, CH_2_OH, COOEt, (cyclopropyl)-methyl, (5-Me-isoxazol-3-yl)-methyl.

A new series of imidazo[1,5-*a*]quinoxalines **94** was described as potential modulators for the identification of an α5-GABA_A_ receptor negative allosteric modulator (NAM) [12]. Recently, the GABA_A_ receptor family has attracted attention due to its regulatory role in the central nervous system, leading to the development of several drugs. However, challenges remain, particularly with respect to α5-GABA_A_. Earlier work [63] reported that oxadiazolylimidazoquinoxalinone derivatives are benzodiazepine analogs acting on GABA_A_ receptors. Furthermore, it was found [64] that compound **A** (Figure 15) acted as NAM at the known benzodiazepine (BDZ) site of both α1β3γ2 and α5β3γ2 receptors.

Considering these data, Karolyi et al. [12] presented compounds **94** as a combination of two known structural motifs targeting the α5-GABA_A_ receptor: the imidazo[1,5-*a*]quinoxaline tricyclic core and the (pyridin-2-yl)methyl group. Using various approaches, they synthesized 50 compounds with a wide variation of the R^1^ substituent (Figure 15) to investigate structure–activity relationships. All derivatives were characterized by ^1^H NMR, ^13^C NMR, and MS spectra. A flumazenil displacement assay was performed for all compounds to evaluate their binding affinity toward α5-GABA_A_ and α1-GABA_A_ receptors. The initial results already demonstrated the usefulness of the chosen approach, with compound **94a** showing strong binding affinity for both receptors (Table 10). The table presents results only for those derivatives of series **94** that exhibited significant binding affinity toward α5 and α1. The data indicate that even minor structural changes in compound **94** can strongly influence functional behavior.

Alongside the determination of binding values, the authors [12] monitored compounds **94** using a functional patch-clamp (PC) assay. Most compounds showed weak modulation of GABA response amplitudes. However, five derivatives exhibited unequivocal efficacy as NAMs, including compound **A** with α5 GABA_A_R modulation of (–37 ± 5), and six derivatives acted as positive allosteric modulators (PAMs), among them compound **94d** with α5 GABA_A_R modulation of 36 ± 7. For a deeper assessment of the SAR results, in silico modeling was employed, and induced-fit docking calculations were performed for several compounds, including **A**, **94a**, and **94d**. These suggested two main possible binding poses (pose A and pose B) for compounds **94a** and **94d**, whereas only one favorable pose (due to steric reasons) was found for compound **A**. Thus, the authors [12] demonstrated the promise of searching among imidazo[1,5-*a*]quinoxalines for nanomolar-active compounds toward the α5-GABA_A_ and α1-GABA_A_ receptors, as well as for positive and negative allosteric modulators.

**Figure 16:**
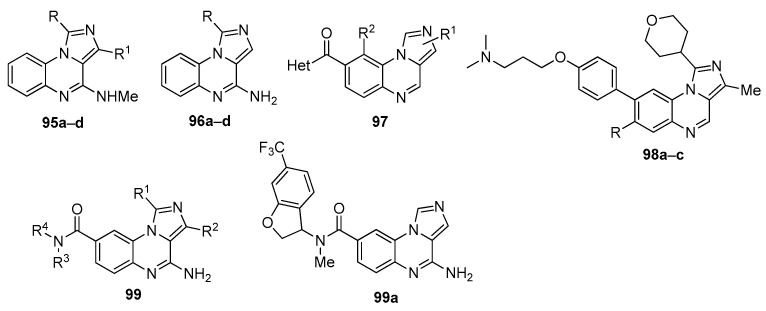
Structure of compounds **95a–d**, **96a–d, 97, 98a–c, 99, 99a. 95:** R = R^1^ = H (**a**); R = H, R^1^ = Br (**b**); R = Br, R^1^ = H (**c**); R = R^1^ = Br (**d**); **96**: R = pentyl (**a**), isopentyl (**b**), hexyl (**c**), Bu (**d**); **98**: R = H (**a**), F (**b**), CF_3_ (**c**).

In continuation of their research on new IKK1 and IKK2 inhibitors within the imidazoquinoxaline series, Patinote et al. [8] synthesized several novel imidazo[1,5-*a*]quinoxalines **95a–d** (Figure 16) using a synthetic pathway analogous to Scheme 1. The IKK1 and IKK2 inhibitory activities of these compounds were tested according to the protocol conditions described [65]. Unfortunately, the new compounds did not show results comparable to or superior to those previously obtained.

Imidazo[1,5-*a*]quinoxalines **96a–d** (Figure 16) [21] were also obtained following a procedure analogous to Scheme 1. These compounds were tested for TLR7 or TLR8 agonistic and antagonistic activities in vitro using HEK-Blue™-hTLR7 and HEK-Blue™-hTLR8 cells. Target products **96** showed very low cytotoxicity at the tested concentrations up to 100 μM as determined by an MTT assay. None of the compounds displayed any TLR7 or TLR8 agonistic activity; however, all of them exhibited selective TLR7 antagonistic activity with IC_50_ values ranging from 17.3 to 45.0 μM, producing 50% inhibition of TLR7 after 24 h of co-incubation with the cells. The most active products were **96b** and **96d** (IC_50_ = 17.7 and 17.3 μM, respectively). It was noted that lengthening the alkyl chain led to a decrease in activity (IC_50_ = 22.0 μM for **96a** and 45.0 μM for **96d**).

Several patents have also reported the synthesis and biological properties of imidazo[1,5-*a*]quinoxaline analogs. For instance, the authors [66] presented a broad series of compounds of general formula **97** exhibiting an inhibitory effect on tumor cells (Figure 16). Compounds **98**, described in patent [67], act as kinase inhibitors and can be used for the treatment of diseases such as cancer. The preparation, pharmaceutical compositions, and use of a large series of imidazo[1,5-*a*]quinoxaline-8-carboxamide compounds **99** as protein arginine methyltransferase 5 (PRMT5) inhibitors were reported [68]. Another patent [69] discloses a crystalline form of one of the compounds **99** (**99a**) (Figure 16). Polycyclic quinoxalines **99** can be used in the treatment of cancer.

Imidazo[1,5-*a*]quinoxalines have drawn less interest than their [1,2-*a*] counterparts, but they do show promising activity as GABAᴀ receptor modulators, TLR7 antagonists, and IKK inhibitors. The best compounds include **94d** (α5-GABAᴀ modulator, *K_i_* = 1.7 nM, PAM activity 36 ± 7%) [12], **96b** and **96d** (TLR7 antagonists, IC_50_ ~17 μM) [9], and **99** (PRMT5 inhibitors with anticancer potential) [69]. SAR work shows that (pyridin-2-yl)methyl and (pyrimidin-5-yl)methyl groups at the *N*-position are key for GABAᴀ binding: even small changes can flip the effect from negative (NAM) to positive (PAM) modulation. For TLR7 antagonism, isopentyl and butyl chains at position 4 work best; longer chains just weaken activity. The biggest drawback of this subclass is that it is still underexplored: fewer than 100 compounds have been reported, and most have not been tested in vivo. Synthetic methods also lag those for the [1,2-*a*] series, with narrower substrate scope and scale-up issues. Going forward, the field would benefit from more diversity-oriented synthesis, proper pharmacokinetic and toxicology studies, and a broader look at GABAᴀ modulators in neurological disease, not just binding affinity.

## 5. Triazoloquinoxalines

The general structures of [1,2,4]triazolo[4,3-*a*]quinoxalines,[1,2,4]triazolo[1,5-*a*]quinoxalines, and [1,2,3]triazolo[1,5-*a*]quinoxalines are shown in Figure 2. Triazoloquinoxalines represent one of the most important classes of azolo-quinoxalines as promising candidates for medicinal chemistry. It is therefore not surprising that the review article [15], devoted to annulated azolo-quinoxalines, contains sections on [1,2,4]triazolo[4,3-*a*]quinoxalines,[1,2,4]triazolo[1,5-*a*]quinoxalines, and [1,2,3]triazolo[1,5-*a*]quinoxalines. It should be noted that later reviews focusing on quinoxaline derivatives [1,14] or benzodiazepines [13] also include brief discussions with references to work on triazoloquinoxalines. In the present article, we discuss publications on the synthesis and properties that were not included in the review [15] or have appeared thereafter.

### 5.1. [1,2,4]-Triazolo[4,3-a]quinoxalines

**Scheme 19:**
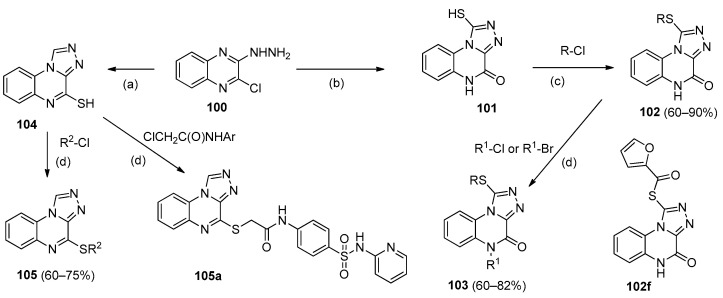
Synthesis of compounds **102**, **103**, **105**: (**a**) trimethyl orthoformate, then thiourea, EtOH, then 10% KOH; (**b**) CS_2_, KOH, EtOH, then HCl; (**c**) DMF/TEA, reflux, 3 h or MW, 4 min or US, 1 h; (**d**) KOH, EtOH. **102:** R = functionalized alkyl, acyl, or heteroaryl substituents introduced via S-alkylation or S-acylation of the mercapto group (e.g., CH_2_COOH, CH_2_COOEt, CH_2_COMe, COCH_2_Cl, CH_2_CONHAr, CO(heteroaryl)); **103:** R, R^1^ = diverse alkyl, iso-alkyl, ester-, or amide-containing substituents introduced via N-functionalization of the triazoloquinoxaline core. For detailed substituent lists and biological activities, see the main text and Table 11. No color coding is used as this scheme illustrates the functionalization of the preformed polycyclic core.

In continuation of research on topoisomerase II (Topo II) inhibitors and DNA intercalators, new [1,2,4]triazolo[4,3-*a*]quinoxaline analogs of **102, 103**, and **105** were synthesized starting from 2-chloro-3-hydrazinoquinoxaline **100** (Scheme 19) [70,71].

The in vitro cytotoxic activity of 1-*RS*-[1,2,4]triazolo[4,3-*a*]quinoxalin-4-ones **102** was evaluated against three tumor cell lines (HCT-116, HepG2, and MCF-7), with IC_50_ values determined and doxorubicin used as a positive control [71]. Compounds **102d–f** and **101** exhibited strong anti-proliferative activities against all three cell lines (Table 11). Cytotoxicity testing of compounds **102d–f** and **101** against a normal human cell line (WI-38) showed that these compounds had low toxicity toward WI-38 (Table 11). The effect of compounds **102d–f** and **101** as Topo II inhibitors was also investigated, and they were found to display activities ranging from 0.45 to 1.06 μM. A DNA intercalation assay was performed for these compounds to determine their DNA-binding affinities, yielding IC_50_ values between 37.06 and 51.23 μM (compared to 31.22 μM for doxorubicin). Further studies were carried out with compound **102f**, which was shown to arrest the cell cycle of HepG2 cells in the G2/M phase. The apoptotic effect of **102f** (32.41%) was five times higher than that of control cells (6.35%). Docking studies of **102f** revealed that this compound exhibited a binding mode similar to that of doxorubicin.

Analogues **103** and **105** were evaluated for their cytotoxic activities against three tumor cell lines (Hep G-2, Hep-2, and Caco-2) [70]. Among them, five compounds showed high activity against all three lines, with IC_50_ values ranging from 0.26 ± 0.1 to 2.91 ± 0.1 μM compared to doxorubicin (0.65 ± 0.1 μM) and were further investigated for their Topo II inhibitory activities and DNA intercalating affinities. The highest activity was exhibited by compound **105a** against Topo II (IC_50_ = 0.97 ± 0.1 μM), which also bound DNA at a concentration of 43.51 ± 2.0 μM. Apoptosis and cell-cycle tests were carried out for this compound, and it was found to induce apoptosis to a significant extent (13.53%) in Hep G-2 cells at a concentration of 0.5 μM.

**Figure 17:**
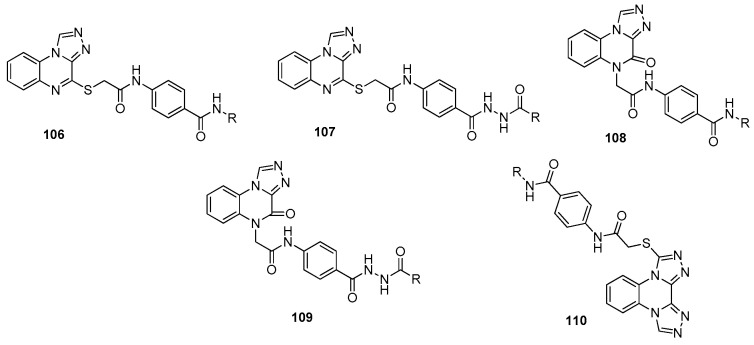
Structure of compounds **106**–**110**. **106**: R = Me, Et, *n*(*iso*)-butyl, *t*-Bu, Ph, 2(3)-MeC_6_H_4_, 2(3)-MeOC_6_H_4_, 2,5-diMeC_6_H_3_, 2,5-diMeOC_6_H_3_, 3(4)-ClC_6_H_4_, 2,5-diClC_6_H_3_, 4-FC_6_H_4_, 2(4)-(OH)C_6_H_4_, 2-(OH)-5-MeC_6_H_3_, 4-NO_2_C_6_H_4_, CH_2_Ph, (CH_2_)_2_Ph, 2-(OH)-4-NO_2_C_6_H_3_, pyridin-2-yl, 4-Cl-pyridin-2-yl, cyclopentyl, cyclohexyl; **107**: R = Ph, 2(3)-ClC_6_H_4_, 4-NO_2_C_6_H_4_; **108**: R = Me, Et, *n*(*iso*)-butyl, Ph, 3-MeC_6_H_4_, 2,5-diMeC_6_H_3_, 2(3)-MeOC_6_H_4_, 2,5-diMeOC_6_H_3_,3(4)-ClC_6_H_4_, 2,5-diClC_6_H_3_, 4-FC_6_H_4_, 2(4)-(OH)C_6_H_4_, 4-NO_2_C_6_H_4_, 2-(OH)-5-MeC_6_H_3_, 2-(OH)-4-NO_2_C_6_H_3_, 4-Cl-pyridine-2-yl, cyclohexyl, CH_2_Ph; **109**: R = Ph, 2(3)-ClC_6_H_4_, 4-NO_2_C_6_H_4_; **110**: R = Et, *n*-butyl, *t*-Bu, cyclopentyl, cyclohexyl, 4-Ac-C_6_H_4_, 3(4)-ClC_6_H_4_, 2,5-diClC_6_H_3_, 4-FC_6_H_4_, 2(4)-(OH)C_6_H_4_, 2-(OH)-4-NO_2_C_6_H_3_.

Alsaif and co-workers described the synthesis and investigation of a new series of [1,2,4]triazolo[4,3-*a*]quinoxaline derivatives as anticancer agents targeting VEGFR-2 kinase [72,73,74,75,76]. Vascular endothelial growth factor receptor-2 (VEGFR-2) plays a crucial role in cancer cell angiogenesis, and the development of new VEGFR-2 inhibitors has attracted considerable attention. *S*-Substituted [1,2,4]triazolo[4,3-*a*]quinoxalines **106, 107** and N-substituted [1,2,4]triazolo[4,3-*a*]quinoxalin-4-ones **108**, **109** (Figure 17) were synthesized starting from 2-chloro-3-hydrazinoquinoxaline **100** (analogously to Scheme 19) [72,73,75,76]. It should be noted that in the series of target products **106** and **108**, the substituent R was widely varied. All synthesized compounds were characterized by IR, ^1^H NMR, and ^13^C NMR spectral data.

Compounds **106**–**109** were investigated for their in vitro anti-proliferative activities against two tumor cell lines (MCF-7 and HepG2) as well as in a VEGFR-2 kinase assay. Most derivatives showed weak activity against MCF-7 and HepG2, with only twelve compounds exhibiting IC_50_ values in the range of 4.3 to 12.2 μM. Among these, compound **106a** demonstrated high activity within the *S*-substituted series, while derivative **108f** was the most potent among the *N*-substituted ones, in both cases compared with sorafenib (Table 12). These same compounds also showed good efficacy as VEGFR-2 inhibitors, again relative to sorafenib. Compound **106a** was further studied for its effects on cell cycle distribution and apoptosis in HepG2 cells, and it was found to disrupt the cell cycle by arresting cells at the G2/M phase. A docking study of **106a** confirmed its binding patterns with the VEGFR-2 active site [72]. Analog **106d**, reported later [76], displayed even higher activity against MCF-7 and HepG2 than **106a**.

Among the described compounds [73], samples **106c** and **108c–e** exhibited significant anticancer efficacy against MCF-7 and HepG2 cells as well as notable inhibitory activity toward VEGFR-2 (Table 12). Compound **108c** was selected for further investigation of its cellular mechanisms and pro-apoptotic effects on HepG2 cells, and it was shown to slow cell population growth by arresting the cell cycle at the G2/M phase (2.14-fold increase). With an extended series of *S*- and *N*-substituted derivatives in hand, the authors examined structure–activity relationships and demonstrated that the [1,2,4]triazolo[4,3-*a*]quinoxalin-4-one scaffold **108** is more favorable for activity than the [1,2,4]triazolo[4,3-*a*]quinoxaline scaffold **106**. This conclusion was partly rationalized by molecular docking results for **108c**, which showed a binding interaction inside the VEGFR-2 active site similar to that of sorafenib.

Compound **108f** proved to be the most potent cytotoxic derivative against MCF-7 and HepG2 among the compounds **108** listed in Table 12, and its VEGFR-2 inhibitory activity was almost comparable to that of sorafenib [75]. Cell cycle analysis and apoptosis assays performed for **108f** revealed that this derivative arrested cell growth in the G2/M phase by 32.55% relative to control cells and was capable of inducing a significant increase in both early and late apoptosis stages (35.10% and 0.67%, respectively).

**Figure 18:**
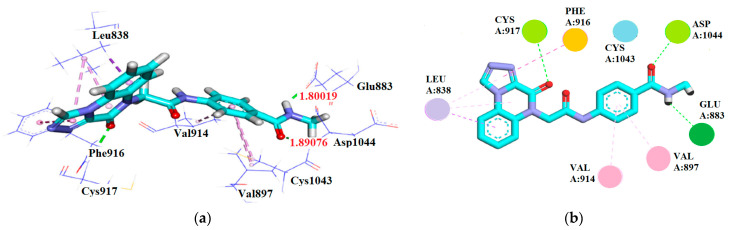
(**a**) 3D and (**b**) 2D diagrams of **108f** in the active site of vascular endothelial growth factor receptor-2. Reproduced with permission of John Wiley & Sons [75].

Docking studies demonstrated that compound **108f** binds to the receptor with high affinity, showing a binding energy of −20.85 kcal/mol. Its amide group forms two hydrogen bonds with the crucial amino acids Glu883 and Asp1044 in the DFG region, mimicking the interaction pattern of sorafenib. Additionally, two hydrophobic contacts with Val914 and Val897 help anchor the phenylacetamide moiety, which occupies the space between the enzyme’s hinge region and the gate area. Within the hinge region, the triazoloquinoxaline moiety engages in four hydrophobic interactions with Leu838 and Phe916, along with one hydrogen bond with Cys917. The terminal methyl group is accommodated within the allosteric site (Figure 18) [75].

New VEGFR-2 inhibitors **110** (Figure 17) based on bis([1,2,4]triazolo)[4,3-*a*:3′,4′-*c*]quinoxaline derivatives were presented as more rigid structures that could potentially enhance binding affinity toward the active site [74]. This series was also evaluated for in vitro anti-proliferative activities against MCF-7 and HepG2 cells as well as in a VEGFR-2 kinase assay. Most products showed low anti-proliferative activity, with only five compounds exhibiting IC_50_ values ranging from 6.4 to 19.5 μM. The highest activity was displayed by compound **110a** (Table 12), which also showed strong VEGFR-2 inhibitory activity. Further studies indicated that derivative **110a** arrested HepG2 cell growth at the G2/M phase and induced apoptosis at a rate of 40.12% compared to control cells (7.07%). Overall, in the authors’ view, compounds **106**, **108**, and **110** possess favorable properties and a satisfactory drug profile.

**Figure 19:**
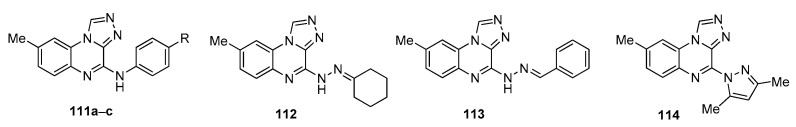
Structure of compounds **111a–c**, **112**–**114**. **111**: R = H (**a**), COOH (**b**), COOEt (**c**).

El-Adl and colleagues, continuing their research, reported new [1,2,4]triazolo[4,3-*a*]quinoxaline analogs **111a–c** and **112**–**114** (Figure 19) as Topo-II inhibitors and DNA intercalators [77,78]. All compounds were synthesized according to reported procedures and characterized by ^1^H NMR, ^13^C NMR, and mass spectral data. The main focus of these studies was the preparation of nanogels as nano-carriers for delivering compounds **111a–c** and **112–114** to cells to increase efficacy and reduce side effects.

A (HEC/PEO) nanogel prepared using hydroxyethyl cellulose and polyethylene oxide was homogeneous and possessed a stable structure [78]. By incorporating each of compounds **111a–c** into the nanogel matrix, the corresponding formulations **111a** (Nanogel), **111b** (Nanogel), and **111c** (Nanogel) were obtained. UV-Vis spectroscopic and dynamic light scattering (DLS) analyses confirmed the presence and integration of each compound within the nanogel structure. Products **111a–c** and their nanogels were evaluated for in vitro cytotoxic activities against A549, MCF-7, HepG2, HCT-116, and VERO cell lines. In all cases, the activity of the nanogels **111a–c** was superior to that of the parent compounds against all tumor cell lines, with compound **111c** and its nanogel showing the highest activity (Table 13). The A549 tumor cells were the most sensitive to derivatives **111a–c**. The nanogels enhanced the cytotoxic actions on A549, HCT116, HepG2, and MCF-7 cancer cells by 31–58% compared to the parent compounds. Furthermore, compounds **111a–c** exhibited low toxicity toward VERO cells. It is also worth noting that nanogels **111a–c** effectively intercalated DNA with IC_50_ values of 31.25, 30.88, and 27.55 μM, respectively, and inhibited Topo-II with IC_50_ values of 1.10, 0.98, and 0.88 μM, which were close to that of doxorubicin (IC_50_ = 0.94 μM).

A nanogel based on sodium carboxymethyl cellulose (Na-CMC) and polyethylene glycol (PEG) was employed [77]. The resulting nanogel formulations **112** (**Nanogel**)–**114** (**Nanogel**) were characterized by UV-Vis and DLS analyses and evaluated for their in vitro cytotoxic activities against A549, MCF-7, HepG2, HCT-116, and VERO cell lines in comparison with the parent compounds **112**–**114**. Again, the nanogels enhanced the cytotoxicity of the derivatives by 28–53%. Compounds **112**–**114** and their nanogels exhibited high selectivity toward cancer cells and low toxicity against VERO cells, with IC_50_ values ranging from 48.29 to 59.70 μM. In this series, compound **114** and its nanogel showed the highest activity against cancer cells (Table 13), although this was lower than that of the best compound from the previous series (**111c**).

**Figure 20:**
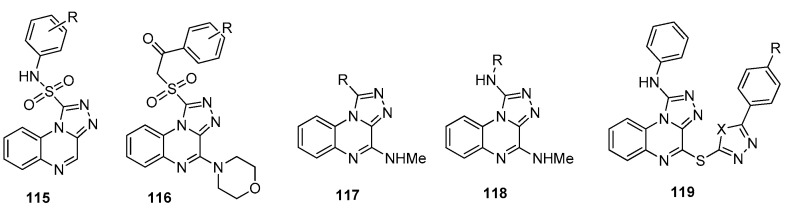
Structure of compounds **115**, **116**, **117**, **118**, **119**. **115**: R = H, Me, OMe, Cl, Br, NO_2_, CN, 2,5-diCl; **116**: R = H, Me, OMe, Br, F, NO_2_, CN, 2,4-diCl, 3,4-diCl, 2,5-diOMe, 3,5-diBr; **117**: R = Bn, (CH_2_)_2_Ph, (CH_2_)_3_Ph, CH=CHPh; **118**: R = Ph, Bn, (CH_2_)_2_Ph; **119**: X = O, S; R = H, Me, OMe, Cl, OH, NH_2_.

Dasari and co-workers synthesized quinoxaline-sulfonyl-1,2,4-triazole hybrids **115** and **116** (Figure 20) and investigated their in vitro anticancer activity [79,80]. The sulfonamide target products **115** were tested against four human cancer lines (MCF-7, HeLa, A549, and IMR32) [79]. Compound **115** (R = 2,5-diCl) exhibited higher activity against all cell lines compared to etoposide (used as the standard), while two other derivatives showed somewhat lower activity.

An extended series of analogs **116** (Figure 20) [80] was tested against four different human cancer cell lines (HepG2, A549, MCF-7, DU-145). The results indicated that the compounds displayed cytotoxic activity ranging from good to moderate, with IC_50_ values between 1.95 and 15.92 μM. High activity was found for five derivatives (Table 14), with compound **116d** being the most potent, showing IC_50_ values comparable to those of etoposide (the standard). SAR analysis led the authors [80] to conclude that the activity within series **116** strongly depends not only on the nature but also on the position of the substituent R. For samples **116a–e**, inhibitory activity against the tyrosine kinase EGFR was also studied, and compounds **116d** and **116c** showed higher activity than etoposide.

[1,2,4]Triazolo[4,3-*a*]quinoxaline analogs **117**–**119** (Figure 20) were obtained [81]. All compounds were investigated for their in vitro effects against human acute myelocytic leukemia HL-60 cells. Compounds **117** and **118** showed relatively weak inhibition even when the concentration was increased to 40 μM; among these, only compound **118** (R = Ph) displayed activity comparable to that of **Imiquimod** (used as a reference). Derivative **119** proved to be more active; at a concentration of 10 μM, most of them exhibited high potency with cell viability below 50%, comparable to that of **118** (R = Ph). The three most active compounds were **119** with X = O and R = Me, OH, and NH_2_, which were additionally evaluated against other cancer cell lines (U937, B16, HepG2). They showed strong antiproliferative effects against HL-60 and U937 cells, comparable to EAPB0203 (reference), and moderate effects against B16 cells; overall, they proved to be more useful compounds than imiquimod and EAPB0203.

**Figure 21:**
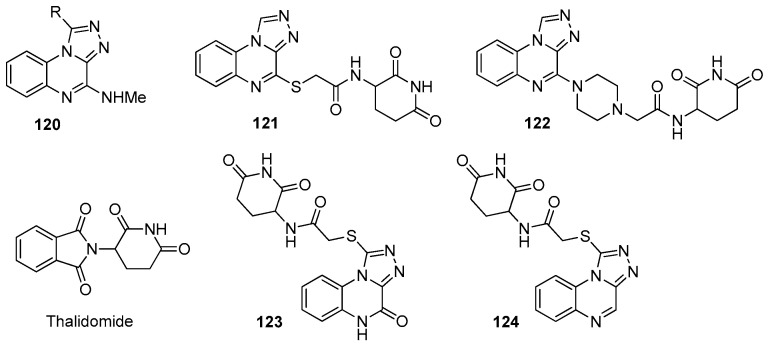
Structure of compounds **120**, **121**–**124**. **120**: R = Alk, Ar.

A series of 1-R-*N*-methyl-[1,2,4]triazolo[4,3-*a*]quinoxalin-4-amines **120** (Figure 21) was synthesized and studied for activity against melanoma cell lines [82]. The compounds were obtained starting from 2-chloro-3-hydrazinoquinoxaline **100**. The reaction of **100** with various aldehydes in DMF at room temperature gave the corresponding hydrazones, and cyclization of the latter under reflux in CHCl_3_ using chloranil as a mild oxidant afforded derivatives **120** in high yields. A375 cell viability was tested at 10 μM for these products, with the best results obtained for compounds **120a** (R = 3,4-diMeOC_6_H_3_), **120b** (R = 3-MeOC_6_H_4_), and **120c** (R = 3,4-diOHC_6_H_3_), showing viability values of 2.9 ± 0.1%, 11.3 ± 6.6%, and 5.5 ± 1.2%, respectively. Inhibitory activities on the A375 melanoma cell line were determined only for these analogs and were found to be in the micromolar range, with EC_50_ values of 3158 nM (**120a**), 3527 nM (**120b**), and 365 nM (**120c**).

It is known that drugs with immunomodulatory properties have shown significant improvements in cancer treatment [83]. On this basis, novel hybrid triazolo-quinoxalines **121–124** (Figure 21) containing a thalidomide fragment (as an immunomodulatory drug) were synthesized [84]. The in vitro cytotoxicity of these compounds against three human cancer cell lines (HepG2, PC3, and MCF-7) was studied in comparison with doxorubicin. The IC_50_ values (μM) of compounds **121** (17.28, 19.12, 14.51) and **123** (9.81, 15.49, 10.09) were found to be comparable to those of doxorubicin (14.61, 16.32, 12.41). These results indicate that the mercaptoacetamide linker is more effective than the piperazinoacetamide one. Products **121** and **123** were also tested for their immunomodulatory activity, and the effect of compound **123** on immune-binding proteins in HepG-2 cells was comparable to that of thalidomide. The 4-oxotriazoloquinoxaline-containing molecule **123** emerged as the most potent candidate, showing better cytotoxic properties than doxorubicin along with immunomodulatory activity comparable to thalidomide. These biological results indicate that the compound can serve as a promising lead molecule for the development of new effective anticancer agents [84].

**Figure 22:**
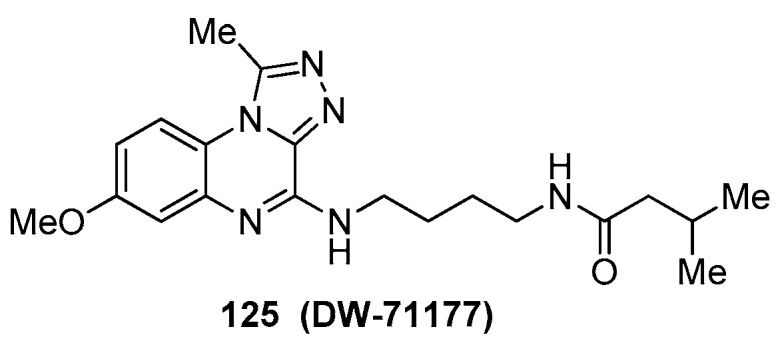
Structure of compound **125**.

In continuation of research on the development of BET (bromodomain and extraterminal domain) inhibitors, Ali et al. [85] described the [1,2,4]triazolo[4,3-*a*]quinoxaline analog **125** (designated DW-71177, Figure 22) as a novel potent and BD1-selective BET inhibitor for the treatment of acute myeloid leukemia. BET proteins play essential roles in cell cycle regulation, and each BET protein contains two tandem bromodomains, namely BD1 and BD2. Product **125** and a series of its analogs with various alkyl chain lengths at position 4 (from ethyl to hexyl) were synthesized according to reported procedures, and their binding affinities for BRD4-BD1 and BRD4-BD2 were measured. The results showed that **125** exhibited significantly higher affinity for BD1 than for BD2. Furthermore, this derivative possessed improved pharmacokinetic properties and was selected for further studies. The growth-inhibitory activity of DW-71177 was tested against various tumor cell lines, and the compound showed strong inhibition of five out of seventeen tested cell lines, with GI_50_ values below 1.0 μM. The in vivo efficacy of DW-71177 was also studied in a human AML xenograft mouse model, revealing a significant reduction in tumor weight and volume at a dose of 120 mg/kg over 21 days. It was discovered that DW-71177 exerts its anticancer effect by suppressing the expression of oncogenes while causing minimal impact on housekeeping genes [85].

**Figure 23:**
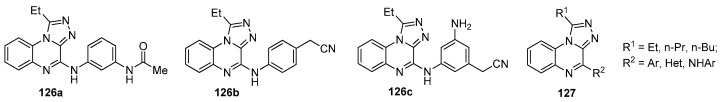
Structure of compounds **126**, **127**.

Pierri and co-workers described the synthesis and comprehensive investigation of functionalized [1,2,4]triazolo[4,3-*a*]quinoxalines targeting BRD9 [86,87,88]. An in silico multidisciplinary approach was reported [86] for the generation of pharmacophore models based on the structure of bromodomain-containing protein 9 (BRD9), facilitating the discovery of new binding agents. Using 23 known ligands co-crystallized with BRD9, three-dimensional pharmacophore models were developed, placed within the reference protein structure, and a pharmacophore model useful for identifying compounds targeting the acetyllysine recognition site was presented. Subsequent pharmacophore-based virtual screening led to the identification of a promising BRD9 inhibitor. Synthesis of analogs in the [1,2,4]triazolo[4,3-*a*]quinoxaline series followed by in vitro studies revealed products **126a–c** (Figure 23) as novel BRD9 inhibitors, which showed high selectivity and exhibited activity with IC_50_ values ranging from 4.20 ± 1.92 μM to 7.48 ± 2.20 μM.

In a subsequent study [87], a thorough structure–activity relationship investigation was conducted on an expanded series of [1,2,4]triazolo[4,3-*a*]quinoxalines **127** (30 new compounds, Figure 23) targeting BRD9 using the previously developed pharmacophore models. Modification at position 1 of the triazoloquinoxaline core involved varying the length of the R^1^ substituent, while at position 4 different spacer types were introduced, including an amine linker, a direct C–C bond, and amide and ether linkers. For all compounds, parameters were evaluated to select the new active derivatives **127a–f** (Table 15) for exploring the chemical space of the bromodomain binding site.

The analysis revealed that the amine spacer is essential for binding to the protein target, while the alkyl substituent at position 1 plays a decisive role in enhancing selectivity toward BRD9. Compounds **127a–f** were able to displace H4Ac (residual binding < 50%) and exhibited IC_50_ values in the low micromolar range. Products **127a** and **127f**, which differ in the R^1^ substituent, were also evaluated against BRD7 and BRPF1; interaction with BRPF1 was observed, suggesting their potential as dual inhibitors. These two derivatives were tested on several leukemia models and showed good efficacy, particularly against the CCRF-CEM cell line, without cytotoxicity toward healthy cells.

In continuation of their research, the authors [88] described a successful process based on in silico, synthetic, and in vitro approaches for the development of BRD9-degrading PROTACs derived from [1,2,4]triazolo[4,3-*a*]quinoxalines using two different E3 ubiquitin ligase ligands. PROTAC (PROteolysis TArgeting Chimera) technology represents a novel and powerful approach that employs small molecules capable of degrading the target protein.

**Scheme 20:**
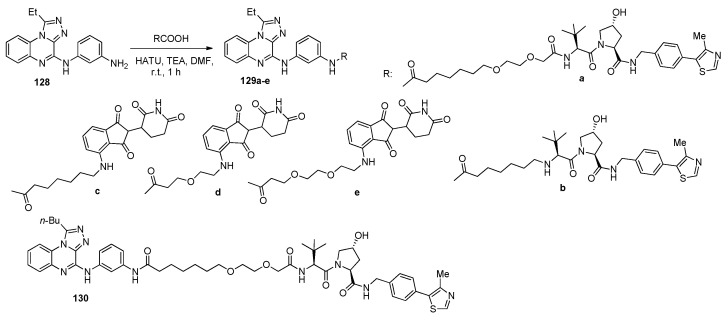
Synthesis of compounds **129a–e**, **130**. No color coding is used, as this scheme illustrates functionalization of the preformed polycyclic core.

Starting from intermediate **128**, compounds **129** containing a VHL E3 ligase ligand (**129a**, **129b**) or a CRBN E3 ligase ligand (**129c–e**) were synthesized (Scheme 20) and characterized by NMR and HRMS. Compound **130**, bearing the same ligand as **129a** but with an *n*-butyl group at position 1 of the triazoloquinoxaline core, was obtained analogously.

All compounds were tested for their degradation activity in a pro-monocytic human myeloid leukemia cell line (U937), which is sensitive to BRD9 downregulation. Cells were treated with compounds **129** and **130** for 48 h at various concentrations (1, 5, 10, 25 μM). The results showed that compounds **129a** and **129d** strongly reduced BRD9 protein levels at concentrations of 10 and 25 μM, whereas no effect was observed for compounds **129b, 129c**, and **129e**. Thus, the length and nature of the linker played a predominant role in the degradation activity of compounds **129**. Compound **130** induced BRD9 degradation at concentrations below 1 μM, exhibiting stronger activity than its analog **129a**.

Consequently, the authors [88] identified two new VHL-based PROTAC compounds (**129a** and **130**) that demonstrated substantial degradation of the target protein and antiproliferative activity in acute myeloid leukemia cells.

**Figure 24:**
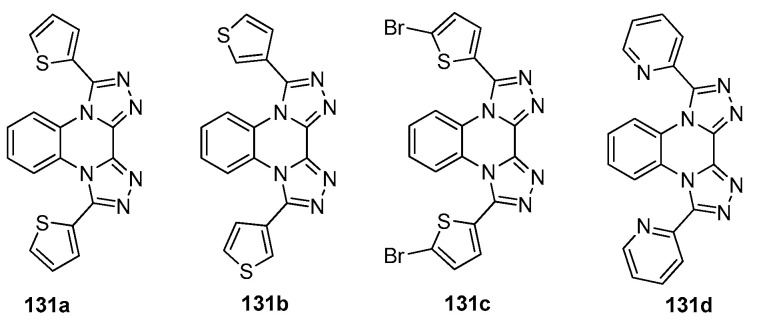
Structure of compounds **131a–d**.

Bis-[1,2,4]triazolo[4,3-*a*]quinoxalines **131a–d** (Figure 24) were reported as potent anticancer agents [89]. These derivatives were synthesized via oxidative cyclization of bis-quinoxalinylhydrazones in the presence of hypervalent iodine(III) in yields of 25–57% and were characterized by ^1^H and ^13^C NMR and mass spectrometry. The cytotoxic activity of products **131a–c** was evaluated against three cancer cell lines (MCF-7, DLD-1, and A549) as well as against human dermal fibroblasts (DF-2). Compounds **131a** and **131b** showed significant toxicity toward MCF-7 and DF-2 cells, with IC_50_ values of 55 and 45 μM for MCF-7 and 93 and 54 μM for DF-2, respectively. However, their toxic effects on DLD-1 and A549 cells were very low. In contrast, compound **131c** demonstrated high activity against A549 cells (IC_50_ = 51 μM), while its IC_50_ value against DF-2 exceeded 1000 μM, indicating its relative safety.

**Scheme 21:**
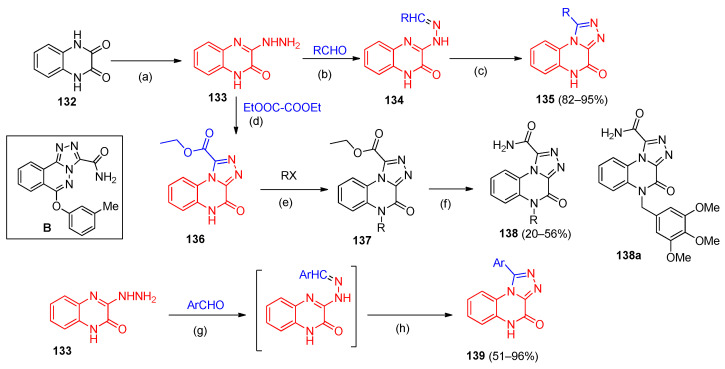
Synthesis of compounds **135**, **138**, **139:** (**a**) 50% NH_2_NH_2_•H_2_O, reflux, 2 h; (**b**) EtOH, reflux, 2 h; (**c**) PhI(OAc)_2_, DCM, room t., 2 h; (**d**) reflux, 3 h; (**e**) K_2_CO_3_, DMF, 60 °C, 3 h; (**f**) NH_4_OH, MeOH, room t., 2 h; (**g**) MeOH, cat. AcOH, 45–90 °C, 1–2 h; (**h**) FeCl_3_•6H_2_O, MeOH, room t., 16 h. **135**: R = Ph, 4-FC_6_H_4_, 4-ClC_6_H_4_, 4-BrC_6_H_4_, 4-MeC_6_H_4_, 4-MeOC_6_H_4_, 2,5-diMeOC_6_H_3_, 3,4-diMeOC_6_H_3_, furan-2-yl, thiophen-2-yl, 2-Br-thiophen-2-yl; **138**: R = *n*-Alkyl, CH_2_Ar. Color coding: blue—one-carbon fragment; red—hydrazinoquinoxaline core.

A convenient and environmentally friendly method for the synthesis of [1,2,4]triazolo[4,3-*a*]quinoxalin-4-ones **135** (Scheme 21) was proposed [90]. The starting quinoxalinedione is readily prepared from *o*-phenylenediamine and oxalic acid in 4 N HCl under reflux for 4 h. Compounds **135** exhibited absorption bands in the range of 305–308 nm, which was quite close to the photoirradiation wavelength of a transilluminator (λ_irr_ = 312 nm) and were not absorbed by DNA. These compounds were therefore used for preliminary DNA photocleavage studies photochemically. It was shown that the DNA-cleaving ability of triazoloquinoxalin-4-ones depends on their structure, concentration, and irradiation time. Mechanistic experiments led to the conclusion that the superoxide anion radical is mainly responsible for the photocleavage. As a result, compound **135** (R = 2-bromothiophen-2-yl) displayed the best cleaving ability and could be a promising candidate for further photobiological applications, such as photodynamic therapy and chemotherapy.

5-Alkyl-4-oxo-4,5-dihydro-[1,2,4]triazolo[4,3-*a*]quinoxaline-1-carboxamides **138** (Scheme 21) were synthesized and investigated [91] as anti-inflammatory agents using RAW264.7 cells. Cytotoxicity studies of compounds **138** by the MTT method at concentrations of 10 or 30 μM showed no obvious cytotoxic effects on RAW264.7 cells, with relative viability of treated cells exceeding 80%. A concentration of 10 μM was therefore selected for subsequent experiments. The anti-inflammatory activity of products **138** was tested based on their ability to inhibit LPS-induced NO production in RAW264.7 macrophages. Compound **138a** exhibited the highest inhibitory activity (38.82%), which was significantly greater than that of lead compound B (Scheme 21). Moreover, derivative **138a** showed more pronounced anti-inflammatory activity than B and the positive control ibuprofen in an in vivo acute inflammation model. Thus, this compound may represent a promising candidate for the treatment of inflammation. Later [92], a one-pot, two-step synthesis of aryl-substituted [1,2,4]triazolo[4,3-*a*]quinoxalin-4-ones **139** starting from hydrazino intermediate **133** (Scheme 21) was described. This approach is useful for large-scale synthesis.

**Scheme 22:**
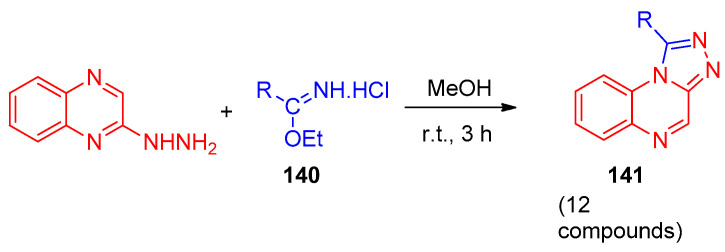
Synthesis of compounds **141**. **141**: R = Me, Bn, CH_2_Ar. Color coding: blue—one-carbon fragment; red—hydrazinoquinoxaline core.

A series of new [1,2,4]triazolo[4,3-*a*]quinoxalines **141** was obtained by cyclocondensation of 2-hydrazinoquinoxaline with iminoester reagents **140** (Scheme 22), and their anti-inflammatory activity was studied [93]. Compounds **141** were characterized by IR, ^1^H NMR, ^13^C NMR, and mass spectral data. Cell viability studies of compounds **141** showed that all of them exhibited IC_50_ values higher than 100 μM. Several compounds were selected for evaluation of anti-inflammatory activity by the nitrite assay. Among them, compound **141** (R = CH_2_(4-ClC_6_H_4_)) showed the highest nitrite-reducing effect (65.12 ± 1.62%), which was close to that of indomethacin (Ind). Molecular docking studies of this product also revealed that it binds to the active site of inducible nitric oxide synthase (iNOS) with high affinity. Overall, compounds **141** hold considerable promise for the discovery of candidates with anti-inflammatory activity.

**Figure 25:**
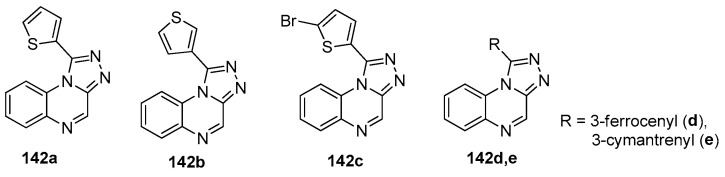
Structure of compounds **142a–e**.

The synthesis of a series of new [1,2,4]triazolo[4,3-*a*]quinoxaline analogs **142** (Figure 25) as potential HSF1 inducers was reported [94]. The compounds were obtained via oxidative cyclization of the corresponding hydrazones using a hypervalent iodine(III) reagent as a mild oxidant. Products **142** were investigated for their ability to activate HSF1 (heat-shock transcription factor 1), which controls the expression of molecular chaperones and therefore represents a therapeutic strategy for the most common degenerative diseases.

Screening of products **142** was conducted using an HSE (heat shock element) system in HeLa cells. It was found that compounds **142a–c** at a concentration of 0.5 μM could increase the degree of HSF1 activation by more than 200%. This is comparable to the activity of the reference compound U133, which elevated HSF1 activity 3.63-fold relative to untreated cells. Furthermore, analogs **142a** and **142c** showed high toxicity toward neuroblastoma SH-SY5Y cells (31.62 ± 0.36% and 31.29 ± 0.48%, respectively), while derivative **142b** exhibited low toxicity (18.99 ± 0.78%). Compounds **142a–c** were also shown to induce Hsp70 expression and reduce the extent of mutant HTT aggregate formation.

**Figure 26:**
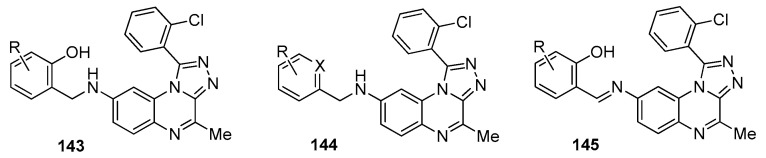
Structure of compounds **143**–**145**. **143**: R = H, OMe, F, Cl, Br; **144**: X = N, CH, R = H, Cl, F; **145**: R = H, 4-F.

Three groups of [1,2,4]triazolo[4,3-*a*]quinoxaline analogs **143**–**145** (Figure 26) were described as new antioxidant ligands targeting inhibition of PDE2 (phosphodiesterase-2), which represents a promising target for Alzheimer’s disease (AD) [95]. Compounds **143**–**145** differ only by the substituent at position 8 of the quinoxaline ring. Their inhibitory activity against PDE2 was investigated in comparison with BAY-60-7550 (reference, IC_50_ = 4.7 nM). Most compounds proved to be potent PDE2 inhibitors with IC_50_ values below 10 nM, and a comparison of their structures revealed that the phenolic hydroxyl group has no significant influence on inhibitory activity. Most analogs **143**–**145** also exhibited excellent antioxidant activity, ranging from 0.2 to 8.6 Trolox equivalents, compared with melatonin (reference, 2.0 Trolox equivalents). Compound **143d** (R = F) displayed excellent inhibitory activity against PDE2 (IC_50_ = 6.1 nM) and antioxidant capacity (8.4 Trolox equivalent) and showed no cytotoxicity toward SH-SY5Y cells. It was therefore selected as the most suitable candidate for further development in vivo studies of AD.

**Figure 27:**
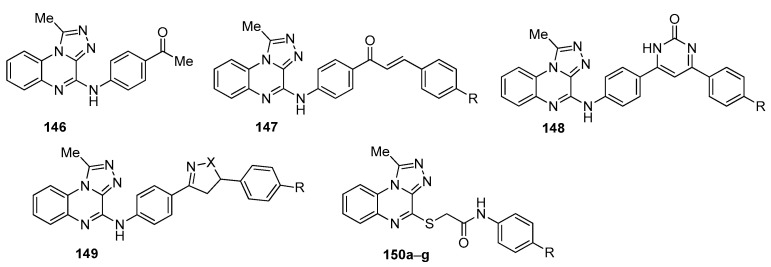
Structure of compounds **146**–**150**. **147**: R = H, Cl, OMe, Me, NO_2_; **148**: R = H, Cl, OMe; **149**: X = NH, O; R = H, Cl, OMe; **150**: H (**a**), Me (**b**), OMe (**c**), Br (**d**), Cl (**e**), F (**f**), NO_2_ (**g**).

New [1,2,4]triazolo[4,3-*a*]quinoxaline analogs **146**–**150** (Figure 27) were reported as potential A_2_B receptor antagonists [96]. The A_2_B receptor is one of the adenosine subtypes, and among its therapeutic properties, antitumor activity is the most significant; evidence suggests that blockade of the human adenosine A_2_B receptor plays an important role in reducing metastasis. The synthesized compounds contain an aryl, styryl, or hetaryl-aryl moiety at position 4 linked to the triazoloquinoxaline core via a hydrophilic NH linker (**146**–**149**) or an extended hydrophilic NHC(O)CH_2_S linker (**150**). The structures of the derivatives were confirmed by various spectroscopic data [96].

For compounds **146**–**150**, cytotoxic activity was evaluated; IC_50_ values were determined against human breast adenocarcinoma cells (MDA-MB-231) using an MTT assay; docking studies and SAR analyses were performed. The results of the cytotoxic activity study showed that only six out of 23 analogs were highly active (**146**, **150a**, **150d–g**), with IC_50_ values ranging from 1.9 to 6.4 μM against the MDA-MB-231 cell line. The observed IC_50_ values of these derivatives were consistent with the obtained docking scores. Overall, the series of compounds **150** proved to be the most potent; among the remaining compounds **146** showed an IC_50_ of 6.4 μM, while all others exhibited values of 15 μM or higher. These observations indicate a significant influence of the nature of the hydrophilic linker and the size of the hydrophobic moiety on compound activity [96].

**Figure 28:**
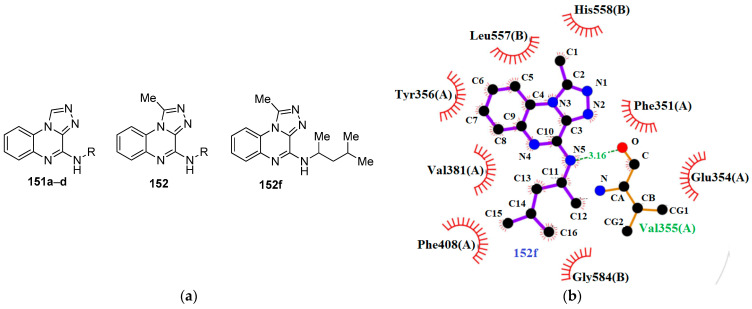
(**a**) Structure of compounds **151a–d**, **152**, **152f**. (**b**) Two-dimensional interaction of compound **152f** with TLR7 protein based on docking studies. Reproduced with permission of Public Library of Science [97]. **151**: R = *iso*-Pr (**a**), *n*-Bu (**b**), 4-Me-pentyl (**c**), NHC_6_H_5_ (**d**); **152**: R = Et (**a**), Pr (**b**), *iso*-Pr (**c**), *sec*-Bu (**d**), *iso*-Bu (**e**), 4-Me-pentyl (**f**), hexyl (**g**), NHC_6_H_5_ (**h**).

New [1,2,4]triazolo[4,3-*a*]quinoxaline amine analogs **151** and **152** (Figure 28a) targeting Toll-like receptor 7 (TLR7) were identified [97]. Toll-like receptors are crucial components of the innate immune system and are important for vaccine development aimed at enhancing immunity against a broad range of pathogens. Among them, TLR7, as an endosomal pattern recognition receptor, plays a key role. The search for new TLR7 ligands was preceded by a series of in silico studies conducted by the authors. Pharmacophore models were developed based on the chemical characteristics of TLR7 using Pharmit (http://pharmit.csb.pitt.edu/) and were subsequently employed for virtual screening of two databases, resulting in the selection of three compounds with the most favorable binding affinity. Among these, [1,2,4]triazolo[4,3-*a*]quinoxaline amine was chosen as the lead structure, and molecular docking studies confirmed the lowest binding energy of these compounds to the active site of TLR7.

Analogs **151** and **152**, bearing predominantly alkyl substituents, were synthesized according to reported procedures in yields up to 80% and were characterized by spectroscopic data. The cytotoxicity of compounds **151** and **152** was evaluated on the J774A.1 mouse macrophage cell line using an MTT assay. Compounds **151a–c** and **152f, 152g** were found to be non-toxic at concentrations below 30 μg/mL; **152a,b,e** were non-toxic below 15 μg/mL; and **152d** showed weak toxicity at 3.75 μg/mL. Six derivatives that were non-toxic and exhibited high binding energy in the docking study were selected for evaluation of cytokine stimulation. Among them, compound **152f**, which induced the highest production of IL-1, TNF-α, and IFN-β cytokines, holds potential as a candidate for immune system stimulation [97].

Harooni and co-workers [97] obtained a two-dimensional binding diagram when investigating the interaction of compound **152f** with Toll-like receptor 7 (TLR7) using molecular docking (Figure 28b). The authors demonstrated that this compound interacts with the same key amino acid residues in the receptor’s active site (including Tyr264(A), Phe351(A), Gln354(A), Val355(A), Tyr356(A), Val381(A), Phe408(A), Lys432(A), Thr532(B), Asp555(B), Leu557(B), Ile585(B), and Thr586(B)) as the known TLR7 agonists resiquimod (R848) and imiquimod. This finding provided an explanation for the low binding energy and high immunostimulatory activity of compound **152f**, which induced the production of IL-1, TNF-α, and IFN-β to an even greater extent than R848.

**Figure 29:**
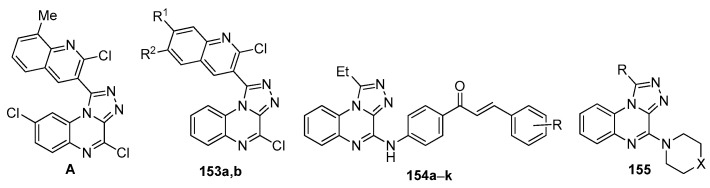
Structure of compounds **153a,b**, **154**. **153**: R^1^, R^2^: H, OMe (**a**), OMe, H (**b**); **154**: R = H (**a**), 2-OMe (**b**), 2-OH (**c**), 4-Me (**d**), 4-OMe (**e**), 4-OH (**f**), 3,4,5-OMe (**g**), 4-NO_2_ (**h**), 4-Cl (**i**), 2,4-Cl (**j**), 2,6-Cl (**k**); **155**: R = H, Me, Ph, 4-FC_6_H_4_, X = CH_2_, O, NMe.

1-Quinolinyl-substituted [1,2,4]triazolo[4,3-*a*]quinoxalines **153** (Figure 29) were described as STING (stimulator of interferon genes) agonists [98]. STING is a signaling adaptor that promotes innate immune signal transduction and participates in the host immune defense against several retroviruses. To identify such structures, the authors performed structure-based virtual screening and selected 64 compounds for in vitro biochemical studies. As a result, structure A was found and identified as a potent STING binder with an IC_50_ of 8.797 μM.

For the subsequently synthesized compounds **153**, biochemical studies were carried out, and it was found that compound **153a** directly binds to the STING CTD (C-terminal domain) and activates the downstream TBK1 (TANK-binding kinase 1)-IRF3 (Interferon regulatory factor 3) signaling pathway. This derivative not only activated human STING in human monocytic THP1 cells but also activated mouse STING in mouse BMDM (bone marrow-derived macrophages) cells, allowing its antiviral and antitumor activity to be tested in mice.

The substituted triazoloquinoxaline chalcone analogs **154** (Figure 28) were synthesized and evaluated for their antimicrobial activity against a panel of pathogenic organisms [99]. The studies were conducted using the filter paper disc diffusion method at a concentration of 128 mg/disc against human pathogenic strains: four fungi (AF, SR, GC, and CA), two Gram-positive bacteria (SP and BS), and two Gram-negative bacteria (PA and EC). The highest activity was exhibited by compound **154e** (bearing a *p*-methoxy group), with minimum inhibitory concentration (MIC) values of 0.49–1.95 mg/mL against the four fungi, 0.98 mg/mL against Gram-positive bacteria (SP, BS), and 0.49 mg/mL against Gram-negative bacteria (EC). Docking studies were performed for this compound, which showed the best binding energy; the results may explain the binding mode of **154e** with target enzymes and its inhibitory activity as an antimicrobial agent.

A series of 1,4-disubstituted [1,2,4]triazolo[4,3-*a*]quinoxalines **155** (Figure 29) was synthesized and investigated against *Schistosoma mansoni* [100]. These derivatives were studied for the first time against the larval stage of this parasite, newly transformed schistosomula (NTS). Derivatives **155** were tested at a concentration of 10 μM, and all showed weak activity (<35% dead) after 72 h at this concentration.

### 5.2. [1,2,3]-Triazolo[1,5-a]quinoxalines

**Scheme 23:**
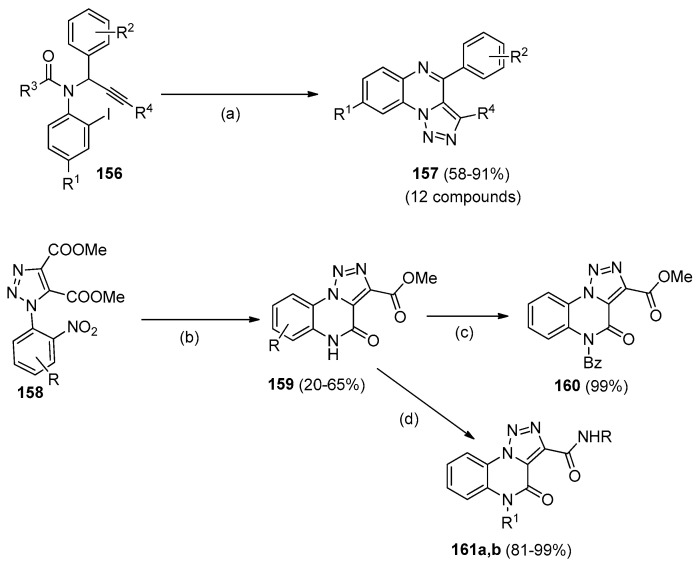
Synthesis of compounds **157**, **159**, **160**, **161a,b**: (**a**) NaN_3_, CuI, *i*-Pr_2_NEt, DMEDA, DMF, 50 °C; (**b**) H_2_, 5%Pd/Al_2_O_3_, room t.; (**c**) BzBr, K_2_CO_3_, 140 °C, 16 h; (**d**) BzNH_2_, room t., 15 min, ultrasound bath. **156**: R^1^ = Me, Cl, F, H; R^2^ = *o, m, p*-Cl, H; R^3^ = Me, Ph; R^4^ = Ar; **159**: R = H, F, OMe, Ph, Ar; **161**: R = Bn, R^1^ = Me (**a**), H (**b**). No color coding is used, as this scheme illustrates two different processes for the formation of the polycyclic core.

In continuation of studies on the use of 2-iodobenzamides as useful reagents for the construction of polycyclic systems, Chen et al. [101] described a novel and efficient synthesis of [1,2,3]triazolo[1,5-*a*]quinoxaline derivatives via CuI-catalyzed reactions of N-propargyl-N-(2-iodoaryl)amides with sodium azide. Using the simple benzamide **156** as a model substrate, optimization of the reaction conditions (solvent, base, ligand, catalyst, temperature, and time) was carried out. The best yield of the target product was achieved when the reaction was performed in DMF with *i*-Pr_2_NEt as the base and DMEDA as the ligand at 50 °C. Under these optimized conditions, a series of [1,2,3]triazolo[1,5-*a*]quinoxalines **157** was prepared (Scheme 23), with yields ranging from moderate to high, and the reaction progress was monitored by TLC. Compounds **157** were characterized by ^1^H NMR, ^13^C NMR, and HRMS-ESI.

The synthesis of [1,2,3]triazolo[1,5-*a*]quinoxalin-4-ones **159** was achieved via cyclization of trisubstituted 1,2,3-triazole **158** (Scheme 23) [102]. Reduction of the nitro group using hydrogen and a catalyst (5% Pd/Al_2_O_3_), followed by spontaneous cyclization, afforded intermediate **159** in moderate yields. Compound **159** (R = H) was subsequently used to prepare N-benzyl analog **160** and amides **161**. It is worth noting that the authors improved several steps of this method, paying particular attention to the use of eco-compatible catalysts and reaction conditions, as well as to the optimization of the procedures.

All compounds **159**, **160**, and **161** were evaluated for antimicrobial properties against different pathogenic bacterial strains to determine whether they could inhibit cell growth, and MIC values were established. None of the compounds exhibited an MIC value within the tested concentrations up to 100 μM. Only three compounds showed a weak ability to affect the growth of the tested microorganisms. Specifically, analogs **160** and **161b** reduced the growth of both *S. aureus ATCC* strains by 15–18% at the highest concentration, and these same derivatives along with **161a** affected the growth of *P. aeruginosa*, with **161a** showing the best activity (23%). Additionally, the activity of analogs **160** and **161a,b** against the growth of *S. epidermidis ATCC 35984* and their ability to form biofilms was tested. Compounds **160** and **161a** were found to reduce biofilm formation by 18% and 23%, respectively.

**Scheme 24:**
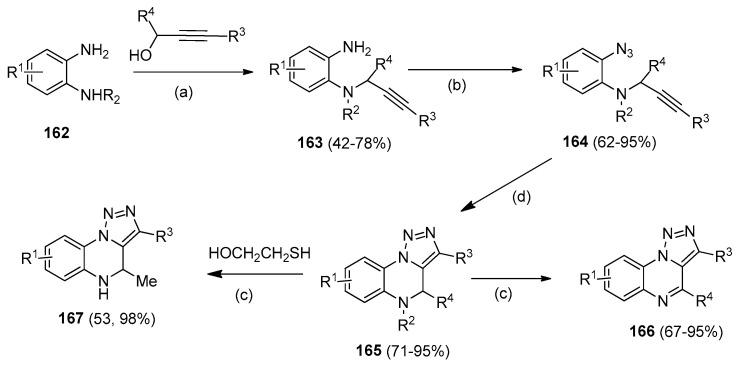
Synthesis of compounds **165**, **166**, **167**: (**a**) TPP, DIAD, THF, room t., 2 h; (**b**) NaNO_2_, HCl, CAN/H_2_O, 0 °C; (**c**) DBU, DMSO, room t.; (**d**) DMSO, 45 °C, 3–5 h. **163**: R^1^ = H, Cl, OH, OMe, diMe, NO_2_, COOMe; R^2^ = 4-MeC_6_H_4_SO_2_ (Ts), 4-NO_2_C_6_H_4_SO_2_ (Ns); R^3^ = H, Me, Et, Ph, 4-CF_3_C_6_H_5_; R^4^ = H, Me; **167**: R^1^ = H, R^3^ = Et, Ph. No color coding is used as one reagent contains all atoms of the polycyclic core.

The synthesis of polysubstituted [1,2,3]triazolo[1,5-*a*]quinoxalines using alkynols as key components was described [103]. The starting materials are sulfonamides **162** (Scheme 24), readily obtained either by direct sulfonylation of commercially available o-phenylenediamines or by sulfonylation and reduction of *o*-nitroanilines. Alkylation of sulfonamides with various prop-2-yn-1-ols in THF in the presence of triphenylphosphine and diisopropyl azodicarboxylate at room temperature afforded compounds **163**. Subsequent one-pot azidation followed by cycloaddition yielded Ts/Ns-dihydrotriazoloquinoxalines **165**. Cleavage of the arylsulfonyl moiety led to products **167**, which can be further modified at the N5 position. Complete aromatization of the triazoloquinoxaline scaffold with elimination of the Ts/Ns group to give products **166** was carried out in DMSO using DBU. All synthesized compounds were characterized by ^1^H NMR, ^13^C NMR, and HRMS-ESI.

The developed method [103] enables the synthesis of a diverse range of [1,2,3]triazolo-annulated heterocycles from inexpensive and readily available starting materials under mild, metal-free conditions in high yields.

Triazoloquinoxalines, particularly the [1,2,4]triazolo[4,3-*a*] regioisomer, represent the most chemically and biologically diverse subclass, with activities spanning anticancer (VEGFR-2, Topo II, BRD9, STING), anti-inflammatory, antimicrobial, and neuroprotective domains. The most potent compounds include **108f** (VEGFR-2 inhibitor, IC_50_ = 3.2 nM; antiproliferative IC_50_ = 4.3–5.8 μM) [75], **DW-71177** (compound **125**, BRD9 inhibitor with in vivo efficacy in AML xenograft models) [85], **135** (DNA photocleavage agent) [90], **141** (anti-inflammatory, 65% nitrite reduction) [93], **143d** (PDE2 inhibitor, IC_50_ = 6.1 nM with antioxidant activity) [95], and **129a/130** (PROTACs for BRD9 degradation) [88]. SAR trends are well-defined: *N*-substituted triazoloquinoxalin-4-ones (**108**) are more potent VEGFR-2 inhibitors than *S*-substituted analogs (**106**); amine linkers and alkyl substituents at position 1 are essential for BRD9 binding; hydrophilic linkers (e.g., NHC(O)CH_2_S) enhance A_2_B receptor antagonism; and phenolic hydroxyl groups contribute to antioxidant and PDE2 inhibitory activities. On the innovation front, this subclass has seen nanogel formulations that improve delivery and efficacy, plus hybrid molecules that fuse triazoloquinoxaline with thalidomide for dual immunomodulatory and anticancer effects. That said, the challenges are still considerable. Most compounds have only been tested in vitro, with little in vivo efficacy or toxicity data. The VEGFR-2 inhibitors are only moderately selective over other kinases. Metabolic stability and oral bioavailability have not been looked at systematically. And PROTACs, though a promising angle, are still at an early stage. Looking ahead, the field needs in vivo validation in relevant animal models, better pharmacokinetic profiles, isoform-selective inhibitors, and more work on PROTACs for targeted protein degradation.

## 6. Critical Assessment of Synthetic Methodologies

The synthetic approaches reviewed here cover a broad spectrum, from classical reactions to modern metal-catalyzed annulations. Nevertheless, their practicality, efficiency, and sustainability differ markedly. Each method has its own pros and cons that must be weighed carefully, particularly when scaling up for medicinal chemistry purposes.

Metal-catalyzed annulations (such as Cu-mediated [3+2] cyclizations, Pd-catalyzed couplings, or Ullmann-type reactions) are generally good for regioselectivity and for building complex, crowded scaffolds. The copper-catalyzed reaction of quinoxalin-2-ones with oxime acetates (Scheme 4) is an excellent example: it gives pyrazolo[1,5-*a*]quinoxalin-4-ones with excellent regiocontrol [11]. On the downside, these methods often require high catalyst loads (5–20 mol%), pricey air-sensitive ligands, and can be picky about functional groups because of the basic or reducing conditions. Scalability is another issue; most examples stop at the gram scale. Removing heavy metal residues down to regulatory levels (e.g., <10 ppm for Pd) drives up both cost and purification effort.

Iodine-mediated or metal-free oxidative cyclizations are cleaner in principle—no toxic metals and generally milder conditions. The I_2_-mediated sp^3^ C–H amination (Scheme 11) [37] and the oxidative [3+2] annulation of quinoxalinones with oxime esters (Scheme 12) [39] are good cases in point: they work for a range of substrates and tolerate various functional groups. That said, these protocols usually need stoichiometric or even excess iodine (1.0–2.0 equiv.), which creates its own waste and cost issues. The iodine-containing byproducts can also make isolation and purification trickier. Regioselectivity is another variable; it often depends on the substrate, and unsymmetrical ones can give mixtures of regioisomers, which limits the appeal for target-directed synthesis.

Multicomponent reactions and cascade processes, like the Ugi/Ullmann sequence (Scheme 9) or the Mo-catalyzed domino reduction-cyclization (Scheme 13), are hard to beat for step- and atom-economy [34,40]. They build complex molecules quickly from simple starting materials, which makes them very attractive for making compound libraries. The catch is that they often have a narrow substrate scope: conditions that work nicely for one set of substrates frequently fall apart when you change the substituents. Also, these one-pot recipes tend to generate several byproducts, which drag down yields (often 40–70%) and complicate purification, especially when the product and impurities behave similarly on a column.

Classical heterocyclizations, for example, condensing hydrazines with aldehydes or carboxylic acid derivatives (Scheme 19 and Scheme 21) [70,71,90], are still workhorses in the field. They are simple to run, reproducible, and easy to scale up. The reagents are cheap; you can make grams of material without trouble, and they tolerate quite a range of functional groups. The trade-off is limited structural diversity: the products usually fall into a narrow range of substitution patterns, and the conditions can be harsh (strong acids, high heat, long reaction times), which is not ideal for sensitive groups.

There is a clear move toward greener methods for making azolo[*a*]quinoxalines. Recent protocols use eco-friendly reductants like *D*-glucose [59], recyclable solid acids such as Wang-OSO_3_H [58], or metal-free oxidative systems with iodine or hypervalent iodine reagents [32,39]. These are certainly kinder to the environment than classical approaches that guzzle strong acids, heavy metals, and nasty organic solvents in stoichiometric amounts. That said, going green often means compromises: metal-free and catalyst-free methods can be less versatile, give lower yields, or be harder to scale up than their conventional counterparts. Developing methods that are both practical and genuinely sustainable is still an open challenge.

So, there is no one-size-fits-all method for the synthesis of azolo[*a*]quinoxalines (Table 16). Which route to pick really depends on what you are trying to achieve. Metal-catalyzed methods are good for getting structurally diverse and novel compounds, though you pay for it in scalability and purification costs. Metal-free oxidative protocols are greener but need careful tweaking of stoichiometry and regioselectivity. MCRs are great for quick diversification, yet their substrate scope is often limited. Classical approaches are still the most practical choice for making known scaffolds on a large scale. Going forward, the real challenge is to close these gaps—to develop protocols that are robust, scalable, and sustainable, combining the broad substrate scope of metal-catalyzed reactions with the simplicity and greenness of metal-free systems.

There is a clear push toward greener, more sustainable ways to make azolo[*a*]quinoxalines. Some recent protocols use eco-friendly reductants like *D*-glucose [59], recyclable solid acids such as Wang-OSO_3_H [58], or metal-free oxidative systems with iodine or hypervalent iodine reagents [32,39]. These are certainly less harmful to the environment than classical methods that consume stoichiometric amounts of strong acids, heavy metals, and unpleasant organic solvents. The flip side is that going green often means trade-offs: metal-free and catalyst-free protocols can be less versatile, give lower yields, or be more difficult to scale up than traditional approaches. Developing methods that are both practical and genuinely sustainable is still an open problem.

## 7. Conclusions

### 7.1. Synthetic and Medicinal Chemistry Advances

The present review has summarized recent advances in the synthesis and biological evaluation of azolo[*a*]quinoxalines, covering pyrazolo[1,5-*a*]quinoxalines, imidazo[1,2-*a*]quinoxalines, imidazo[1,5-*a*]quinoxalines, and triazoloquinoxalines. Most biologically active derivatives share common structural features: the quinoxaline nucleus often remains unsubstituted at position 2 or bears oxo, amino, aryl, alkoxy, N-alkyl, or N-aryl groups. This structural uniformity suggests a privileged scaffold for further optimization.

A wide range of synthetic building blocks has been employed, including quinoxalin-2-ones, quinoxalinedione, 3-arylquinoxalin-2-amines, 2-hydrazinoquinoxaline, 2-chloro-3-hydrazinoquinoxaline, 2-((benz)imidazol-1-yl)anilines, 2-(methylsulfonyl)-3-phenylquinoxaline, *N*-propargyl-N-(2-iodoaryl)amides, 2-amino-N-alkynylsulfonamides, and bis-quinoxalinylhydrazones. These versatile intermediates have enabled diverse synthetic methodologies, such as Cu-catalyzed oxidative [3+2] annulation, [3+2] cycloaddition of tricyclic sydnones with arynes, four-component cyclizations, I_2_-mediated direct sp^3^ C–H amination, I_2_-mediated oxidative [3+2] annulation of quinoxalinones with oxime esters, cycloaddition of 2-(methylsulfonyl)-3-phenylquinoxaline with ethyl isocyanoacetate, and [5+1] cascade cyclizations using elemental sulfur as a catalyst [62]. Additionally, reusable catalysts (for example, Wang-OSO_3_H) and eco-friendly reductants (e.g., *D*-glucose) have been successfully applied, reflecting a growing trend toward sustainable chemistry.

Importantly, the review highlights a steady increase in the use of computational methods. Virtual screening of commercial and in-house chemical libraries, molecular docking, molecular mechanics, and pharmacophore-based virtual screening [99] have been widely adopted to identify new lead compounds, rationalize structure–activity relationships, and guide structural optimization [28,29]. In several cases, a combination of in silico and in vitro approaches [43,88] has led to the successful discovery of selective inhibitors and allosteric modulators. Notably, multidisciplinary in silico strategies have been employed to generate three-dimensional pharmacophore models [88,89], enabling the identification of novel BRD9 binders and TLR7 modulators.

Beyond their well-documented anticancer properties, azolo[*a*]quinoxalines exhibit a remarkable diversity of biological activities. Pyrazolo[1,5-*a*]quinoxalines act as Toll-like receptor antagonists, monoamine oxidase inhibitors, opioid receptor modulators, and PI3Kα inhibitors. Indazolo[2,3-*a*]quinoxalines display antiproliferative effects. Imidazo[1,2-*a*]quinoxalines function as kinase inhibitors, tubulin polymerization inhibitors, α_5_-GABAᴀ receptor modulators, and TLR7 antagonists. Triazoloquinoxalines show anticancer activity (including VEGFR-2 targeting), BRD9 inhibition, anti-inflammatory effects, HSF1 induction, PDE2 inhibition, TLR7 antagonism, STING agonism, and antimicrobial properties.

Across all azolo[*a*]quinoxaline subclasses, several overarching trends emerge. Imidazo[1,2-*a*]quinoxalines are the most extensively studied, with the highest number of compounds and the most advanced in vivo data (**EAPB02303**). Triazolo[4,3-*a*]quinoxalines show the greatest chemical and biological diversity, including the most potent enzyme inhibitors (VEGFR-2, PDE2) and innovative delivery systems (nanogels, PROTACs). Pyrazolo[1,5-*a*]quinoxalines offer a balance of synthetic accessibility and target diversity but lack in vivo validation. Imidazo[1,5-*a*]quinoxalines remain underexplored despite promising GABAᴀ modulatory activity. A common limitation across all subclasses is the reliance on in vitro assays with insufficient pharmacokinetic and toxicological profiling. Future drug discovery efforts should prioritize systematic in vivo efficacy and safety studies, selectivity profiling against related targets, optimization of metabolic stability and oral bioavailability, and development of scalable, sustainable synthetic methodologies.

Other recent highlights include nanogels as delivery vehicles for triazoloquinoxalines and hybrid molecules that fuse the triazoloquinoxaline core with thalidomide fragments for combined immunomodulatory and anticancer effects. Together, these approaches offer fresh ways to boost the therapeutic potential of this heterocyclic family.

To give a quick snapshot of the most promising compounds, Table 17 pulls together the best-in-class examples from each azolo[*a*]quinoxaline subclass. For each entry, the table shows the scaffold type, a representative compound, its biological target, activity value, selectivity, key structural feature, and reference. This comparison highlights both the structural versatility of the scaffold and the most advanced candidates in terms of potency, selectivity, and in vivo data.

As evident from Table 17, imidazo[1,2-*a*]quinoxalines (exemplified by **EAPB02303**) and [1,2,4]triazolo[4,3-*a*]quinoxalines (exemplified by **DW-71177** and **108f**) currently represent the most advanced scaffolds, with in vivo efficacy and/or nanomolar potency against validated therapeutic targets. The lack of clinical candidates, though, is a clear signal that pharmacokinetics, selectivity, and safety still need attention—as mentioned above.

### 7.2. Clinical Translation Status and Future Perspectives

For all the progress made in synthesizing and testing azolo[*a*]quinoxalines, the fact remains that, to the best of our knowledge, none of them have made it to the clinic or even into late-stage trials. A huge amount of research shows potent activity against all sorts of targets in vitro, but that has not yet turned into an approved drug. This disconnects between what works in the lab and what works in the clinic comes down to several familiar hurdles that plague the development of new heterocyclic scaffolds.

One major hurdle is the PK profile. These compounds often have poor solubility, low oral bioavailability, and fast metabolic clearance, which makes it hard to reach effective concentrations in vivo. The physicochemical properties of these polycyclic systems can also cause off-target toxicity and unexpected side effects—problems that often do not show up until advanced safety studies. Metabolic stability is another weak spot; many compounds are easily chewed up by cytochrome P450 oxidation, which undermines the in vivo efficacy seen in cell-based assays. And then there is selectivity: getting a compound to hit one target over closely related isoforms (e.g., other kinases) is tough, and promiscuity shrinks the therapeutic window.

Even so, the future for azolo[*a*]quinoxalines still looks bright. New synthetic methods make it easier than ever to generate diverse libraries quickly, which helps tune lead compounds for better ADME and toxicity profiles. Computational chemistry is also playing a bigger role: structure-based design and machine learning are speeding up the hunt for candidates with better selectivity and pharmacodynamics. Innovative formulations, like the nanogels covered in this review, offer practical ways around solubility and bioavailability problems. And then there are hybrid molecules and PROTACs, which represent a real shift in thinking; they could sidestep some of the toxicity issues that plague conventional inhibitors and unlock the full potential of this scaffold. The road to the clinic is not easy, but the structural variety and broad biological activity of azolo[*a*]quinoxalines guarantee that they will keep inspiring drug discovery for years to come.

In summary, azolo[*a*]quinoxalines represent a flexible and promising platform for medicinal chemistry. The increasing integration of computational design, sustainable synthesis, and innovative drug delivery systems is expected to accelerate the discovery of new clinical candidates targeting a wide range of diseases.

## Figures and Tables

**Table 1:** TLR7-antagonistic activities of pyrazolo[1,5-*a*]quinoxalines **6a–d** in HEK-Blue™-hTLR7 cells [21].

Compound	IC_50_ (μM) ^1^	Compound	IC_50_ (μM)
**6a**	8.2 ± 1.6	**6b**	10.0 ± 1.7
**6c**	30.6 ± 17.5	**6d**	16.3 ± 6.8

^1^ IC_50_ values represent the concentration of compound (μM) producing 50% inhibition of TLR7-mediated NF-κB activation after 24 h of co-incubation with HEK-Blue™-hTLR7 cells in the presence of 1 μg/mL CL264 (TLR7 agonist). Each experiment was repeated at least three times; results are presented as mean ± standard deviation.

**Table 2:** The human MAO inhibition potencies of pyrazolo[1,5-*a*]quinoxalin-4-ones **10–12, 14–16** against recombinant human MAO-A and MAO-B isoforms [25].

Compound	IC_50_ (μM) to MAO-A	IC_50_ (μM) to MAO-B	SI ^1^
**10L** (R^1^ = 4-ClC_6_H_4_, R^2^ = NH_2_)	0.345 ± 0.0057	>100	>290
**11f** (R^1^ = 4-MeC_6_H_4_, R^2^ = CN)	5.10 ± 0.131	0.617 ± 0.044	0.12
**11g** (R^1^ = 4-MeOC_6_H_4_, R^2^ = CN)	>100	0.763 ± 0.035	0.008
**12c** (R^1^ = 4-MeC_6_H_4_, R^2^ = CN)	10.9 ± 8.10	0.674 ± 0.003	0.06
**14a** (R^1^ = Ph)	0.181 ± 0.0079	>100	>552
**14b** (R^1^ = 4-MeOC_6_H_4_)	0.155 ± 0.0051	11.9 ± 0.827	77
**14c** (R^1^ = 4-ClC_6_H_4_)	0.028 ± 0.0039	1.40 ± 0.087	50
**15a** (R^1^ = Ph)	0.146 ± 0.020	64.4 ± 11.5	441
**15b** (R^1^ = 4-MeOC_6_H_4_)	0.472 ± 0.293	7.90 ± 0.470	17
**16a** (R^1^ = Ph)	0.173 ± 0.010	4.16 ± 0.887	24
**16b** (R^1^ = 4-MeOC_6_H_4_)	0.951 ± 0.087	7.37 ± 1.78	7.7
**Curcumin**			
(reference inhibitor)	5.02 ± 0.45	2.56 ± 0.21	0.51

^1^ The selectivity index (SI) was calculated as the ratio of IC_50_ (MAO-B) to IC_50_ (MAO-A).

**Table 3:** Antagonistic effect of compounds **19a–h** against human opioid receptors (KOP, MOP, DOP) in HEK293T cells [11].

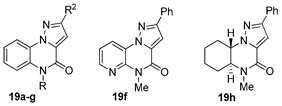								
Compound	R	R^2^	KOP	MOP		DOP		
			EC_50_ (μM)	*E*_max_ (%)	EC_50_ (μM)	*E*_max_ (%)	EC_50_ (μM)	*E*_max_ (%)
**19a**	Me	Ph	>10	NA ^1^	2.08	109	2.06	82
**19b**	Et	Ph	>10	NA	0.35	33	1.14	87
**19c**	*^n^*Pr	Ph	6.77	135	1.66	116	1.47	150
**19d**	*^n^*Bu	Ph	>10	NA ^1^	>10	NA	5.60	97
**19e**	Allyl	Ph	4.63	198	1.82	390	2.49	312
**19f**			>10	NA	8.48	109	7.9	112
**19g**	CH_2_CO_2_Et	4-MeOC_6_H_4_	>10	NA	>10	NA	5.42	28
**19h**			>10	NA	11	130	14	104

^1^ NA—Not Available.

**Table 4:** Class I PI3K inhibitory activity of compounds **28a,b** and **29a–c** against human recombinant PI3K isoforms [29].

Compound	Class I PI3K IC_50_ (μM)			
	PI3Kα	PI3Kδ	PI3Kꞵ	PI3Kɤ
**28a**	0.87	5.74	0.72	1.40
**28b**	0.24	4.10	10	0.83
**29a**	>20	>20	4.89	>20
**29b**	>20	>20	15.04	>20
**29c**	>20	>20	>20	>20

**Table 5:** The IC_50_ values of benz[4,5]imidazo[1,2-*a*]quinoxalines **55f–i** against human cancer cell lines and selectivity over normal breast epithelial cells (MCF12A) [38].

Compound	Cell Lines (IC_50/_μM)					Selectivity Index (SI)
	MDA-MB-231 (Breast, TNBC)	MDA-MB-468 (Breast, TNBC)	MCF7 (Breast)	HEK293 (Normal Kidney)	MCF12A (Normal Breast)	
**55f**	15.88	17.28	7.85	>100	44.28	>2.8 (vs. MCF7)
**55g**	25.57	5.24	18.77	>100	45.32	>8.6 (vs. MDA-MB-468)
**55h**	16.8	3.2	2.59	>100	32.07	>12.4 (vs. MDA-MB-468)
**55i**	14.62	4.78	1.55	~10	1.04	<1 (non-selective)
**Cisplatin**	3.62	1.43	4.23	–	–	–

**Table 6:** Antiproliferative activity (IC_50_, μM) of imidazo[1,2-*a*]quinoxalines **69a–d** and acyclic analogs **70a,b** against human cancer cell lines [42,43].

Compound	Cell Lines (IC_50/_μM)			
	MCF-7	MDA-MB-2321	A549	HCT-116
**69a**	4.34 ± 0.12	4.73 ± 0.06	5.21 ± 0.11	4.96 ± 0.31
**69b**	9.12 ± 0.11	7.74 ± 0.21	8.36 ± 0.15	9.53 ± 0.14
**69c**	13.18 ± 0.52	11.68 ± 0.23	11.26 ± 0.26	16.39 ± 0.25
**69d**	4.33 ± 0.31	6.11 ± 0.23	5.44 ± 0.18	5.87 ± 0.31
**70a**	8.47 ± 0.32	11.18 ± 0.41	7.68 ± 0.28	9.85 ± 0.18
**70b**	5.67 ± 0.13	8.18 ± 0.05	7.17 ± 0.15	6.43 ± 0.07
**colchicine**	5.11 ± 0.33	5.14 ± 0.35	6.55 ± 0.41	5.54 ± 0.33

**Table 7:** Antiproliferative activity (IC_50_, nM) of **EAPB02303** and **EAPB0503** against human melanoma cell lines compared to **vemurafenib** [45].

Cell Lines	IC_50_ (nM)			BRAF Mutation
	EAPB02303	EAPB0503	Vemurafenib	
A375	3	383	139	v600e
ME WO	3	125	283	-
A2058	4	185	425	v600e
IPC 298	40	469	13,000	-

**Table 8:** Antiproliferative activity (IC_50_, nM) of imidazo[1,2-*a*]quinoxalines **74b**, **75h**, **75j**, **76a** and **76b** against human cancer cells [48].

Compound	IC_50_ (nM) ± SEM		
	A549	HCT-116 WT	MDA-MB-231
**74b**	2.7 ± 0.032	5.1 ± 0.029	4.1 ± 0.031
**75h**	4.09 ± 0.024	<1	11.2 ± 0.022
**75j**	8.75 ± 0.028	<1	2.2 ± 0.026
**76a**	6.63 ± 0.031	>25	14.1 ± 0.021
**76b**	12.06 ± 0.021	7.90 ± 0.027	13.6 ± 0.028
**Erlotinib**	4.56 ± 0.019	2.98 ± 0.023	3.33 ± 0.018

**Table 9:** Antiproliferative activity (IC_50_, μM) of imidazo[1,2-*a*]quinoxalines **77a–e** against human cancer cell lines [50].

Compound	R	IC_50_ (μM) ± SD		
		A549 (Lung)	MCF-7 (Breast)	MDA-MB-231 (Breast)
**77a**	3-MePh	3.53 ± 0.03	13.58 ± 0.10	5.77 ± 0.03
**77b**	4-ClPh	4.43 ± 0.08	3.82 ± 0.14	5.94 ± 0.04
**77c**	3-F-6-NO_2_Ph	3.25 ± 0.02	9.99 ± 0.15	11.36 ± 0.04
**77d**	3-thienyl	1.34 ± 0.04	14.30 ± 0.30	>25
**77e**	3-pyridyl	4.27 ± 0.14	23.85 ± 0.24	8.66 ± 0.11
**Erlotinib**		4.68	>10	>10

**Table 10:** Binding affinity (*K_i_*, nM) of imidazo[1,5-*a*]quinoxalines **A**, **94a–i** for α5-GABA_A_ and α1-GABA_A_ receptors determined by flumazenil displacement assay [12] ^1^.

Compound	R	R^1^	R^2^	α5 *K_i_* (nM)	α1 *K_i_* (nM)
**A**				0.5	0.4
**94a**	H	(pyridin-2-yl)-methyl	H	132	52
**94b**	H	(pyridin-3-yl)-methyl	H	28	32
**94c**	H	(pyrimidin-5-yl)-methyl	H	15	30
**94d**	H	(2-Me-pyrimidin-5-yl)methyl	H	1.7	3.6
**94e**	H	(pyridin-3-yl)-methyl	Me	18	8
**94f**	H	(pyridin-2-yl)-methyl	Cl	12	5.4
**94g**	H	(pyridin-3-yl)-methyl	COOEt	0.3	0.4
**94h**	7-Cl	(pyridin-3-yl)-methyl	H	11	15

^1^ Binding affinity was determined by flumazenil displacement assay at 1 μM compound concentration.

**Table 11:** Antiproliferative activity (IC_50_, μM) of [1,2,4]triazolo[4,3-*a*]quinoxalines **101, 102d–f** against human cancer cell lines [71].

Compound	IC_50_ (μM)			
	HCT-116	HepG2	MCF-7	WI-38
**101**	7.70 ± 0.5	5.98 ± 0.3	6.38 ± 0.5	64.28 ± 3.7
**102d** (R = COCH_2_Cl)	9.61 ± 0.8	3.48 ± 0.2	5.16 ± 0.4	57.13 ± 3.5
**102e** (R = CH_2_CONH-(4-MeC_6_H_4_))	12.86 ± 1.2	8.07 ± 0.6	15.85 ± 1.2	38.92 ± 2.8
**102f** (R = CO(furyl-2))	10.23 ± 1.0	2.81 ± 0.2	7.28 ± 0.7	42.74 ± 3.0
**Doxorubicin**	4.50 ± 0.2	4.17 ± 0.2	5.23 ± 0.3	not tested

**Table 12:** Antiproliferative activity (IC_50_, μM) and VEGFR-2 inhibitory activity (nM) of [1,2,4]triazolo[4,3-*a*]quinoxalines **106**, **108**, **110** [72,73,74,75,76].

Compound	R	IC_50_ of Anti-Proliferative Activity (μM)		VEGFR-2 (nM)	Reference
		MCF-7	HepG2		
**106a**	Ph	8.2	5.4	3.4	[74]
**106b**	4-Cl-pyridin-2-yl	12.2	8.3	3.9	[74]
**106c**	2,5-diClC_6_H_3_	11.7 ± 1.1	9.7 ± 0.7	5.1 ± 0.4	[75]
**106d**	*t*-Bu	6.2	4.9	3.9	[77]
**106e**	2-MeOC_6_H_4_	8.9 ± 1.0	6.7 ± 0.9	5.4 ± 0.6	[78]
**108a**	3-MeC_6_H_4_	10.3	6.5	3.9	[74]
**108b**	CH_2_Ph	9.7	7.9	4.7	[74]
**108c**	4-ClC_6_H_4_	7.2 ± 0.6	4.1 ± 0.4	3.4 ± 0.3	[75]
**108d**	2,5-diClC_6_H_3_	10.3 ± 1.1	8.4 ± 0.6	4.8 ± 0.3	[75]
**108e**	2-(OH)-5-MeC_6_H_3_	8.1 ± 0.5	7.8 ± 0.4	3.9 ± 0.3	[75]
**108f**	Me	5.8 ± 0.7	4.3 ± 0.5	3.2 ± 0.4	[78]
**108g**	(CH_2_)_2_Ph	7.9 ± 0.8	5.8 ± 0.4	4.8 ± 0.3	[78]
**110a**	2,5-diClC_6_H_3_	10.3 ± 0.8	6.4 ± 0.5	3.7 ± 0.2	[76]
**Sorafenib**		3.51 ± 0.2	2.17 ± 0.7	3.12 ± 0.3	[78]

**Table 13:** Antiproliferative activity (IC_50_, μM) of [1,2,4]triazolo[4,3-*a*]quinoxalines **111a–c** and their nanogel formulations against human cancer cell lines [78].

Compound	IC_50_ (μM)				
	A549	HCT116	HepG2	MCF-7	VERO
**111a**	5.90 ± 0.5	9.80 ± 1.5	7.95 ± 0.5	7.68 ± 0.5	49.90 ± 2.9
**111a** (Nanogel)	3.78 ± 0.5	6.44 ± 1.5	3.98 ± 0.5	3.77 ± 0.5	45.90 ± 2.9
**111b**	4.53 ± 0.5	8.86 ± 1.1	6.44 ± 0.5	6.13 ± 0.8	46.80 ± 2.9
**111b** (Nanogel)	3.12 ± 0.5	5.84 ± 1.1	3.45 ± 0.5	3.09 ± 0.8	49.50 ± 2.9
**111c**	3.77 ± 0.5	7.47 ± 1.1	5.56 ± 0.5	5.94 ± 0.5	48.30 ± 2.9
**111c** (Nanogel)	2.30 ± 0.5	4.20 ± 1.1	2.35 ± 0.5	2.48 ± 0.5	46.87 ± 2.9
**114**	5.50 ± 0.5	9.17 ± 1.1	7.12 ± 0.5	7.85 ± 0.5	57.44 ± 2.9
**114** (Nanogel)	3.75 ± 0.5	5.85 ± 1.1	3.56 ± 0.5	3.72 ± 0.5	49.68 ± 2.9
**Doxorubicin**	8.54 ± 0.8	8.07 ± 0.8	7.94 ± 0.6	6.75 ± 0.4	ND ^1^

^1^ ND—No Data.

**Table 14:** Antiproliferative activity (IC_50_, μM) of quinoxaline-sulfonyl-1,2,4-triazole hybrids **116a–e** against human cancer cell lines [80].

Compound	IC_50_ (μM)				
	R	HepG2	A 549	MCF-7	DU-145
**116a**	4-NO_2_	6.12 ± 0.42	6.26 ± 1.13	5.15 ± 0.10	5.92 ± 1.17
**116b**	4-OMe	5.52 ± 0.17	6.78 ± 0.09	6.89 ± 0.11	5.44 ± 0.41
**116c**	4-CN	4.46 ± 0.92	5.12 ± 0.49	6.23 ± 0.10	5.10 ± 0.43
**116d**	3,4-diCl	2.20 ± 0.28	3.02 ± 1.31	2.03 ± 0.22	1.95 ± 1.34
**116e**	2,5-diOMe	7.32 ± 0.48	6.51 ± 0.44	ND ^1^	9.61 ± 1.72
**Etoposide**		2.39 ± 1.56	3.08 ± 0.13	2.11 ± 0.24	1.97 ± 0.45

^1^ ND—No Data.

**Table 15:** In silico parameters and detected binding towards BRD9 of compounds **127a–f**, residual binding of acetylated-histone H4 (H4Ac) to BRD9, IC_50_ [87].

Compound	R^1^	R^2^	Docking Score (kcal/mol)	Phase Screen Score	Num Sites Matched	Residual Binding (%)	IC_50_ (μM)
**127a**	Et	NHC_6_H_4_-4-CMe_2_CN	−5.96	1.25	7/7	30.47 ± 1.38	3.93 ± 0.54
**127b**	Et	NH(Py-3)-4-CH_2_CN	−6.02	1.38	7/7	46.89 ± 0.44	8.24 ± 1.44
**127c**	Et	NHC_6_H_4_-4-C(cyclopentyl)CN	−5.29	1.52	7/7	40.24 ± 0.52	4.41 ± 1.16
**127d**	Et	NHC_6_H_4_-3-SO_2_(CH_2_)_2_OH	−6.40	1.42	7/7	45.14 ± 0.13	8.31 ± 0.85
**127e**	Et	NHC_6_H_4_-4-O_2_N=(NH_2_)NH	−6.70	1.52	7/7	41.20 ± 4.83	5.26 ± 1.15
**127f**	*n*-Bu	NHC_6_H_4_-4-CMe_2_CN	−4.97	1.34	7/7	41.28 ± 2.17	6.73 ± 1.55
**Bromosporine**						14.63 ± 1.00	0.42 ± 0.07

**Table 16:** Comparative assessment of synthetic methodologies for azolo[*a*]quinoxalines.

Methodology	Representative Examples	Yield Range	Substrate Scope	Scalability	Sustainability	Regioselectivity	Functional-Group Tolerance	Key Limitations
Cu/Pd-catalyzed annulations	Scheme 4, Scheme 9 and Scheme 23	40–91%	Broad (aryl, heteroaryl, alkyl)	Moderate (mg–g scale)	Low (heavy metals, ligands)	High (often controlled by catalyst/ligand)	Moderate (sensitive to basic conditions, coordinating groups)	Metal contamination, high cost, air sensitivity
I_2_-mediated oxidative cyclizations	Scheme 7, Scheme 11 and Scheme 12	44–93%	Moderate to broad	Moderate	Moderate (stoichiometric I_2_, byproducts)	Substrate-dependent	Good (tolerates halogens, ethers, esters)	Stoichiometric oxidant, purification challenges
Multicomponent reactions (MCRs)	Scheme 9, Scheme 10 and Scheme 16	50–85%	Narrow to moderate	Good (one-pot)	Moderate to high	High (predetermined by design)	Moderate (limited by compatibility of all components)	Substrate scope limitations, byproduct formation
Classical heterocyclizations	Scheme 1, Scheme 2, Scheme 15, Scheme 19 and Scheme 21	60–95%	Moderate	Excellent (multi- gram to kg)	High (simple reagents, recyclable catalysts)	High (predictable)	Good to excellent	Limited structural diversity, harsh conditions often required
Metal-free cascade/domino	Scheme 7, Scheme 13, Scheme 16, Scheme 18 and Scheme 24	58–91%	Moderate to broad	Moderate to good	High (no metals, eco-friendly reductants)	High to moderate	Good	Emerging methodology, scope not fully explored

**Table 17:** Summary of the most promising azolo[*a*]quinoxaline scaffolds, representative compounds, and their biological activities.

Scaffold Type	Representative Compound	Biological Target	Activity Value	Selectivity	Key Structural Feature	Reference
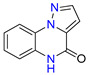 Pyrazolo[1,5-*a*]quinoxalin-4-one	**14c**	MAO-A	IC_50_ = 0.028 μM	High over MAO-B (SI > 50)	N-acetylated; 4-ClC_6_H_4_ substituent	[25]
	**19c**	Opioid receptors (DOR, MOR, KOR)	EC_50_ = 1.47–6.77 μM	Pan-antagonist (DOR > MOR > KOR)	N-*n*-propyl; 3-phenyl substituent	[11]
	**28b**	PI3Kα	IC_50_ = 0.24 μM	4- to 40-fold over other PI3K isoforms	7-Amino; 4-methylpyrimidinyl-methyl	[29]
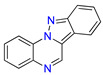 Indazolo[2,3-*a*]quinoxaline	**48b**	Antiproliferative (HCT116)	IC_50_ = 2.1 μM	Not reported	4-ClC_6_H_4_; *tert*-butyl isocyanide	[34]
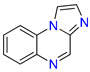 Imidazo[1,2-*a*]quinoxaline	**74b**	EGFR (WT and T790M mutant)	IC_50_ = 211 nM (WT); 3.65 μM (H1975)	Selective over normal cells (HBL-100)	3,4,5-Trimethoxyphenyl	[48]
	**69d**	Tubulin (colchicine site)	IC_50_ = 4.33–6.11 μM	Comparable to colchicine	3,4-Dimethoxyphenyl	[43]
	**71** (**EAPB02303**)	Melanoma (A375 cells; in vivo xenograft)	IC_50_ = 3 nM	Active against BRAF V600E and wild-type	3,4-Dihydroxyphenyl; 8-amino	[45]
	**78a**	Antifungal (*Valsa mali*)	EC_50_ = 5.6 μg/mL	Broad-spectrum fungicidal	4-Hydrazineyl; 7-methyl	[51]
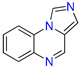 Imidazo[1,5-*a*]quinoxaline	**94d**	α5-GABAᴀ receptor (PAM)	*K_i_* = 1.7 nM (α5); 3.6 nM (α1)	PAM activity (36 ± 7%)	(2-Methylpyrimidin-5-yl)methyl	[12]
	**96b**	TLR7 antagonist	IC_50_ = 17.7 μM	Selective over TLR8	5-Isopentyl	[9]
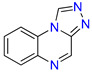 [1,2,4]Triazo-lo[4,3-*a*]quinoxaline	**108f**	VEGFR-2 kinase	IC_50_ = 3.2 nM	Comparable to sorafenib	N-Methyl; 4-oxo	[75]
	**125** (**DW-71177**)	BRD9 (BD1-selective)	IC_50_ = 0.42 μM (BRD9); in vivo efficacy	BD1-selective over BD2	4-Butyl; 5-(pyridin-3-yl)	[85]
	**129a**/**130**	BRD9 degrader (PROTAC)	Degradation at 1–25 μM (BRD9)	Selective degradation	VHL E3 ligand; variable linker	[88]
	**143d**	PDE2	IC_50_ = 6.1 nM	Antioxidant (8.4 Trolox eq.)	8-Fluoro; 4-(3,4-dihydroxyphenyl)	[95]
	**141** (R = CH_2_-4-ClC_6_H_4_)	Anti-inflammatory (iNOS)	65% nitrite reduction	Comparable to indomethacin	4-Chloro-benzyl	[93]
	**152f**	TLR7 agonist	Immunostimulation (cytokine production)	Low cytotoxicity (J774A.1)	4-Methyl-pentyl	[97]
	**154e**	Antimicrobial (broad- spectrum)	MIC = 0.49–1.95 mg/mL	Active against fungi, Gram-(+/−)	4-Methoxy-chalcone	[99]
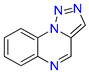 [1,2,3]Triazo-lo[1,5-*a*]quinoxaline	**165**/**166**/**167**	Antimicrobial (biofilm inhibition)	18–23% biofilm reduction	Moderate activity	Ts/Ns-protected; metal-free synthesis	[103]

## Data Availability

No data was created or analyzed during this study. Data sharing is not applicable.

## Abbreviations

The following abbreviations are used in this manuscript:

DABCO	1,4-Diazabicyclo[2.2.2]octane
DBU	1,8-Diazabicyclo[5.4.0]undec-7-ene
DCM	Dichloromethane
DEA	*N,N*-Diethylaniline
DIAD	Diisopropylazadicarboxylate
DMAP	4-Dimethylaminopyridine
DMEDA	*N*,*N*′-Dimethylethylenediamine
DMF	*N,N*-Dimethylformamide
DMSO	Dimethylsulfoxide
DOR	δ-opioid receptor
EGFR	Epidermal Growth Factor Receptor
GABA	Gamma-aminobutyric acid
H4Ac	Acetylated histone H4
HSF1	Heat-shock transcription factor 1
IC_50_	Half-maximal inhibitory concentration
IKK	IκB kinase (inhibitor of nuclear factor kappa-B kinase)
iNOS	Inducible nitric oxide synthase
KOR	κ-opioid receptor
MAO	Monoamine oxidase
MCR	Multicomponent reaction
MOR	μ-opioid receptor
MTT	3-(4,5-Dimethylthiazol-2-yl)-2,5-diphenyltetrazolium bromide
NAM	Negative allosteric modulator
NaHMDS	Sodium bis(trimethylsilyl)amide
NMR	Nuclear magnetic resonance
PAM	Positive allosteric modulator
PDE2	Phosphodiesterase-2
PI3K	Phosphoinositide 3-kinase
PRMT5	Protein arginine methyltransferase 5
PPhMe_2_	Dimethylphenylphosphine
PROTAC	PROteolysis TArgeting Chimera
SAR	Structure–activity relationship
SEM	Standard error of the mean
SI	Selectivity index
STING	Stimulator of interferon genes
Syk	Spleen tyrosine kinase
TBAF	Tetrabutylammonium fluoride
TEA	Triethylamine
TFA	Trifluoroacetic acid
THF	Tetrahydrofuran
TLR	Toll-like receptor
TMEDA	Tetramethylethylenediamine
TopoII	Topoisomerase II
TPP	Triphenylphosphine
VEGFR2	Vascular endothelial growth factor receptor 2

## References

[1] Abdelall E.K.A., Elshemy H.A.H., Ryad N., Abdelaziz A.A. (2025). Exploring the Anticancer Properties of Quinoxaline-Based Molecules: A Systematic Review. Chem. Biodivers..

[2] Mukhortov L.G., Alexandrov N.S., Karimov M.B., Sidyakov S.A., Shakhkeldyan I.V., Atroshchenko Y.M. (2025). A Review of the Synthesis of Quinoxalines and Their Derivatives. Butlerov Commun..

[3] Irfan A., Sabeeh I., Umer M., Naqvi A.Z., Fatima H., Yousaf S., Fatima Z. (2017). A Review on the Therapeutic Potential of Quinoxaline Derivatives. World J. Pharm. Res..

[4] Dawson S., Malkinson J.P., Paumier D., Searcey M. (2007). Bisintercalator natural products with potential therapeutic applications:isolation, structure determination, synthetic and biological studies. Nat. Prod. Rep..

[5] Zolova O.E., Mady A.S.A., Garneau-Tsodikova S. (2010). Recent developments in bisintercalator natural products. Biopolymers.

[6] Fernández J., Marín L., Álvarez-Alonso R., Redondo S., Carvajal J., Villamizar G., Villar C.J., Lombó F. (2014). Biosynthetic Modularity Rules in the Bisintercalator Family of Antitumor Compounds. Mar. Drugs.

[7] Hattori K., Koike K., Okuda K., Hirayama T., Ebihara M., Takenaka M., Nagasawa H. (2016). Solution-phase synthesis and biological evaluation of triostin A and its analogues. Org. Biomol. Chem..

[8] Patinote C., Karroum N.B., Moarbess G., Deleuze-Masquef C., Hadj-Kaddour K., Cuq P., Diab-Assaf M., Kassab I., Bonnet P.-A. (2017). Imidazo[1,2-*a*]pyrazine, Imidazo[1,5-*a*]quinoxaline and Pyrazolo[1,5-*a*]quinoxaline derivatives as IKK1 and IKK2 inhibitors. Eur. J. Med. Chem..

[9] Shukla N.M., Kimbrell M.R., Malladi S.S., David S.A. (2009). Regioisomerism Dependent TLR7 Agonism and Antagonism in an Imidazoquinoline. Bioorg. Med. Chem. Lett..

[10] Tripathi A.C., Upadhyay S., Paliwal S., Saraf S.K. (2018). Privileged scaffolds as MAO inhibitors: Retrospect and prospects. Eur. J. Med. Chem..

[11] Yadav A., Yadav A., Tripathi S., Dewaker V., Kant R., Yadav P.N., Srivastava A.K. (2022). Copper-Catalyzed Oxidative [3+2]-Annulation of Quinoxalin-2(1*H*)-one with Oxime Esters toward Functionalized Pyrazolo[1,5-*a*]quinoxalin-4(5*H*)-ones as Opioid Receptor Modulators. J. Org. Chem..

[12] Károlyi B.I., Potor A., Kapus G.L., Fodor L., Bobok A., Krámos B., Magdó I., Bata I., Szabó G. (2023). Novel imidazo[1,5-*a*]quinoxaline derivatives: SAR, selectivity and modeling challenges en route to the identification of an α5-GABA_A_ receptor NAM. Bioorg. Med. Chem. Lett..

[13] Nafie M.S., Fahmy S.A., Kahwash S.H., Diab M.K., Dawood K.M., Abbas A.A. (2025). Recent advances on anticancer activity of benzodiazine heterocycles through kinase inhibition. RSC Adv..

[14] Montero V., Montana M., Carré M., Vanelle P. (2024). Quinoxaline derivatives: Recent discoveries and development strategies towards anticancer agents. Eur. J. Med. Chem..

[15] Patinote C., Cirnat N., Bonnet P.-A., Deleuze-Masquefa C. (2021). Fused Azolo-Quinoxalines: Candidates for Medicinal Chemistry. A Review of their Biological Applications. Curr. Med. Chem..

[16] Dadiboyena S., Nefzi A. (2011). Synthesis of functionalized tetrasubstituted pyrazolyl heterocycles—A review. Eur. J. Med. Chem..

[17] Janin Y.L. (2012). Preparation and Chemistry of 3/5-Halogenopyrazoles. Chem. Rev..

[18] Li M., Zhao B.-X. (2014). Progress of the synthesis of condensed pyrazole derivatives (from 2010 to mid-2013). Eur. J. Med. Chem..

[19] Perez-Fernandez R., Goya P., Elguero J. (2014). A review of recent progress (2002–2012) on the biological activities of pyrazoles. ARKIVOC.

[20] Faria J.V., Vegi P.F., Miguita A.G.C., dos Santos M.S., Boechat N., Bernardino A.M.R. (2017). Recently reported biological activities of pyrazole compounds. Bioorg. Med. Chem..

[21] Bou Karroum N., Moarbess G., Guichou J.-F., Bonnet P.-A., Patinote C., Bouharoun-Tayoun H., Chamat S., Cuq P., Diab-Assaf M., Kassab I. (2019). Novel and selective TLR7 antagonists among the imidazo[1,2-*a*]pyrazines, imidazo[1,5-*a*]quinoxalines and pyrazolo[1,5-*a*]quinoxalines series. J. Med. Chem..

[22] Zhang S., Hu Z., Tanji H., Jiang S., Das N., Li J., Sakaniwa K., Jin J., Bian Y., Ohto U. (2018). Small-Molecule Inhibition of TLR8 through Stabilization of Its Resting State. Nat. Chem. Biol..

[23] Panova V.A., Korsakov M.K., Shetnev A.A., Filimonov S.I. (2019). Synthesis of substituted 5-hydroxypyrazolo[1,5-*a*]quinoxalin-4-ones. Mendeleev Commun..

[24] Panova V.A., Ivanovskii S.A., Shetnev A.A., Chirkova Z.V., Sudzilovskaya T.N., Filimonov S.I. (2020). Synthesis of substituted pyrazolo[1,5*-a*]quinoxalines using the reductive cyclization. Russ. Chem. Bull..

[25] Panova V.A., Filimonov S.I., Chirkova Z.V., Kabanova M.V., Shetnev A.A., Korsakov M.K., Petzer A., Petzer J.P., Suponitsky K.Y. (2021). Investigation of pyrazolo[1,5-*a*]quinoxalin-4-ones as novel monoamine oxidase inhibitors. Bioorg. Chem..

[26] Chirkova Z.V., Kabanova M.V., Filimonov S.I., Abramov I.G., Petzer A., Engelbrecht I., Petzer J.P., Suponitsky K.Y., Veselovsky A.V. (2018). An investigation of the monoamine oxidase inhibition properties of pyrrolo[3,4-*f*]indole-5,7-dione and indole-5,6-dicarbonitrile derivatives. Drug Dev. Res..

[27] Mostert S., Petzer A., Petzer J.P. (2015). Indanones as high-potency reversible inhibitors of monoamine oxidase. ChemMedChem.

[28] Gu D., Cheng G., Zhang M., Zhou Y.-B., Li J., Sheng R. (2021). Discovery of 2-(5-(quinolin-6-yl)-1,3,4-oxadiazol-2-yl)acetamide derivatives as novel PI3Kα inhibitors via docking-based virtual screening. Bioorg. Med. Chem..

[29] Gu D., Zhang M., Cai L., Zhang M., Zhou Y.-B., Li J., Sheng R. (2023). Discovery of 4-oxo-4,5-dihydropyrazolo[1,5-*a*]quinoxaline-7-carboxamide derivatives as PI3Kα inhibitors via virtual screening and docking-based structure optimization. Bioorg. Med. Chem..

[30] Sheng R., Zhou Y.-B., Gu D., Li J., Zhang M., Zhang M., Yang H., Cai L. Preparation of 4,5-Dihydropyrazolo[1,5-*a*]quinoxalin-4-one Compounds and Its Application. Patent CN116621841 A, 22 August 2023. https://patents.google.com/patent/CN116621841A/en?oq=CN116621841.

[31] Khanal B., Jaithum K., Feliciano M.A.M., Gold B. (2025). Catalytic Ullmann-Type*N*-Arylation to Access Medicinally Important SuFEx-able Pyrazolo[1,5-*a*]Quinoxalinones. Asian J. Org. Chem..

[32] Arockiaraj M., Singh G., Rajeshkumar V. (2025). I_2_/DMSO Mediated [5+1] Oxidative Annulation of Aryl Methyl Ketones with 2-(2*H*-Indazol-2-yl)anilines for the Synthesis of Indazoloquinoxalines. Asian J. Org. Chem..

[33] Louis M., Talbot A., Lamaa D., Sallustrau A., Audisio D., Taran F. (2022). A sydnone-based route to access indazolo[2,3-*a*]quinoxaline derivatives. Synlett.

[34] Li Y., He L., Qin H., Liu Y., Yang B., Xu Z., Yang D. (2024). A Facile Ugi/Ullmann Cascade Reaction to Access Fused Indazolo-Quinoxaline Derivatives with Potent Anticancer Activity. Molecules.

[35] Mamedov V.A., Kalinin A.A. (2014). Advances in the synthesis of imidazo[1,5-*a*]- and imidazo[1,2-*a*]quinoxalines. Russ. Chem. Rev..

[36] Rezaei Z., Mehdi Didehvar M., Mahdavi M., Azizian H., Hamedifar H., Mohammed E.H.M., Ostad S., Amini M. (2019). Anticancer properties of N-alkyl-2, 4-diphenylimidazo[1,2-*a*]quinoxalin-1-amine derivatives; kinase inhibitors. Bioorg. Chem..

[37] Chen W., Du Y., Wang M., Fang Y., Yu W., Chang J. (2020). Synthesis of Benzo[4,5]imidazo[1,2-*a*]quinoxalines by I_2_-Mediated *sp*^3^ C−H Amination. Org. Chem. Front..

[38] Singh R., Kumar R., Pandrala M., Kaur P., Gupta S., Tailor D., Malhotra S.V., Salunke D.B. (2021). Facile synthesis of C6-substituted benz[4,5]imidazo[1,2-*a*]quinoxaline derivatives and their anticancer evaluation. Arch. Pharm..

[39] Kumar P., Kant R., Srivastava A.K. (2025). Iodine mediated oxidative [3+2] annulations of quinoxalinones with oxime esters: An approach towards imidazo[1,2-*a*]quinoxalin-4(5*H*)-one. Synlett.

[40] Gуmez-Gil S., Cases N., Hernández-Ruiz R., Rubio-Presa R., Suárez-Pantiga S., Pedrosa M.R., Sanz R. (2024). Molybdenum-Catalyzed One-Pot Multi-Step Synthesis of *N*-Polyheterocycles from Nitroarenes and Glycols. Eur. J. Org. Chem..

[41] Yang S., Tan X., Liu D., Jiang H., Wu W. (2025). Photoredox-Catalyzed Radical Alkylation/Cyclization of Isocyanides with Organoboron Reagents for Diversified Synthesis of *N*-Heteroaromatics. Adv. Synth. Catal..

[42] Goel K.K., Rajput S.K., Kharb R. (2023). Design, Synthesis and Biological Evaluation of Imidazole-Substituted/Fused Aryl Derivatives Targeting Tubulin Polymerization as Anticancer Agents. SynOpen.

[43] Goel K.K., Hussain A., Altamimi M.A., Rajput S.K., Sharma P.P., Kharb R., Mahdi W.A., Imam S.S., Alshehri S., Alnemer O.A. (2023). Identification of Potential Antitubulin Agents with Anticancer Assets from a Series of Imidazo[1,2-*a*]quinoxaline Derivatives: In Silico and In Vitro Approaches. Molecules.

[44] Patinote C., Cirnat N., Hadj-Kaddour K., Cuq P., Bonnet P.-A., Deleuze-Masquéfa C. (2020). Substantial Cellular Penetration of Fluorescent Imidazoquinoxalines. J. Fluoresc..

[45] Patinote C., Deleuze-Masquéfa C., Kaddour K.H., Vincent L.-A., Larive R., Zghaib Z., Guichou J.-F., Assaf M.D., Cuq P., Bonnet P.-A. (2021). Imidazo[1,2-*a*]quinoxalines for melanoma treatment with original mechanism of action. Eur. J. Med. Chem..

[46] Karroum N.B., Patinote C., Deleuze-Masquéfa C., Moarbess G., Diab-Assaf M., Cuq P., Kassab I., Bonnet P.-A. (2018). Methylation of imidazopyrazine, imidazoquinoxaline, and pyrazoloquinoxaline through Suzuki–Miyaura cross coupling. Chem. Heterocycl. Compd..

[47] Deleuze-Masquefa C., Bonnet P.-A., Cuq P., Patinote C. (2016). New Imidazo[1,2-*a*]quinoxalines and Derivatives Thereof for the Treatment of Cancer.

[48] Kumar M., Joshi G., Arora S., Singh T., Biswas S., Sharma N., Bhat Z.R., Tikoo K., Singh S., Kumar R. (2021). Design and Synthesis of Non-Covalent Imidazo[1,2-*a*]quinoxaline-Based Inhibitors of EGFR and Their Anti-Cancer Assessment. Molecules.

[49] Bhat Z.R., Kumar M., Sharma N., Yadav U.P., Singh T., Joshi G., Pujala B., Raja M., Chatterjee J., Tikoo K. (2021). In Vivo Anticancer Evaluation of 6b, a Non-Covalent Imidazo[1,2-*a*]quinoxaline-Based Epidermal Growth Factor Receptor Inhibitor against Human Xenograft Tumor in Nude Mice. Molecules.

[50] Kumar M., Patil K.T., Maity P., Chatterjee J., Singh T., Joshi G., Singh S., Kumar R. (2024). Design, synthesis, and anticancer assessment of structural analogues of (*E*)-1-((3,4,5-trimethoxybenzylidene)amino)-4-(3,4,5-trimethoxyphenyl)-imidazo[1,2-*a*]quinoxaline-2-carbonitrile (**6b**), an imidazo[1,2-*a*]quinoxalinebased non-covalent EGFR inhibitor. RSC Med. Chem..

[51] Ma T., Zhong X., Yang Y., Liu W., Guo B., Fan J., Tang L., Fan L., Li Y. (2024). Synthesis and evaluation of imidazo[1,2-*a*]quinoxaline derivatives as potential antifungal agents against phytopathogenic fungi. Mol. Divers..

[52] Li Z., Tian Q., Lin Y., Gao H., Bai F. (2024). Preparation of Quinoxaline Derivatives. https://patents.google.com/patent/CN117384164A/en?oq=CN117384164.

[53] Lee K.S., Kwon S.O., Yun Y.J., Cho J.H., Lee N.C., Kim J.E., Sook J. (2019). Heterocyclic Derivatives as H4 Histamine Receptor Inhibitors and Their Preparation. https://patents.google.com/patent/WO2019231270A1/en?oq=WO2019231270.

[54] Quin W., Wang H., Tan L., Chen S. (2021). 1H-Pyrazolederivative and Application Thereof as Dual Target Inhibitor SYK and VEGFR2. https://patents.google.com/patent/WO2021169958A1/en?oq=WO2021169958.

[55] Liu Y., Quin W., Wang H., Tan L., Chen S. (2022). Preparation Method of Spleen Tyrosine Kinase and Vascular Endothelial Growth Factor 2 Dual-Target Inhibitor. https://patents.google.com/patent/CN114591338A/en?oq=CN114591338.

[56] Liu Y., Quin W., Wang H., Tan L., Chen S. (2022). Preparation Method of SYK and VEGFR2 Duai-Target Inhibitor, and Application Thereof Drugs Preventing or Treating SYK and VEGFR2 Mediated Diseases. https://patents.google.com/patent/CN114621232A/en?oq=CN114621232.

[57] Wang H., Tan L., Qian W., Chen S. (2022). Salt Form and Crystal Form of Pyrazole Substituted Imidazo[1,2-*a*]quinoxaline Derivative. https://patents.google.com/patent/WO2022166548A1/en?oq=WO2022166548.

[58] Simhachalam G., Rao L.V., Chiranjeevi Y., Rao D., Ramamohan M., Krishnaji T., Raghunadh A. (2022). A simple and efficient synthesis of imidazoquinoxalines and spiroquinoxalinones via pictect-spengler reaction using Wang resin. Synth. Commun..

[59] Simhachalam G., Sekhar C., Rao L.V., Rao R., Raghunadh A. (2023). Glucose as an eco-friendly reducing agent for a one-pot multicomponent synthesis of quinoxalines. Synth. Commun..

[60] Chiranjeevi Y., Kumar G.R., Rao L.V., Golagani S., Simhachalam G., Satyender A. (2024). Sequential Decarboxylation, Substitution, Cyclization and Oxidation with Iodine-DMSO Catalytic System: A new Methodology for the Construction of Imidazoquinoxaline Motifs. ChemistrySelect.

[61] Sahana M.N., Kavya K., Honnabandar K.V., Swaroop T.R., Mantelingu K. (2025). Base induced cycloaddition of ethyl/methyl isocyanoacetate with 2-(methylsulfonyl)-3-(het)arylquinoxalines: A new pathway to access ethyl/methyl 4-(het)arylimidazo[1,5-*a*]quinoxaline-3-carboxylates. Tetrahedron.

[62] Ghosh S., Das D., Mandal R.D., Das A.R. (2024). Pragmatic Access to Hybrid Quinoxaline Scaffold Mediated by Elemental Sulfur Enabling Actualization to ***π***-Extended and Aza-Annulated Heterocyclic Units. J. Org. Chem..

[63] Hansen H.C., Watjen F. (1989). Preparation of Oxadiazolylimidazoquinoxalinone Derivatives as Central Nervous System Agents.

[64] Carling W.R., Ladduwahetty T., MacLeod A.M., Merchant K.J., Moore K.W., Sternfeld F., Street L.J. (1998). Substituted 1,2,4-Triazolo[3,4-*a*]phthalazine Derivatives as gaba Alpha 5 Ligands. https://patents.google.com/patent/WO1998050385A1/en?oq=WO1998050385.

[65] Moarbess G., Guichou J.F., Paniagua-Gayraud S., Chouchou A., Marcadet O., Leroy F., Ruedas R., Cuq P., Deleuze-Masquefa C., Bonnet P.A. (2016). New IKK inhibitors: Synthesis of new imidazo[1,2-*a*]quinoxaline derivatives using microwave assistance and biological evaluation as IKK inhibitors. Eur. J. Med. Chem..

[66] Chen Z., Mei H., Tian Q., Song H., Ge J. (2025). Preparation of Imidazo[1,5-*a*]quinoxaline Derivatives and Its Application. https://patents.google.com/patent/CN119431378A/en?oq=CN119431378.

[67] Cai S., Tian Y., Wang X. (2021). Preparation of (Aryl)imidazo[1,5-*a*]quinoxalines as Kinase Inhibitors. https://patents.google.com/patent/CN112480120A/en?oq=CN112480120.

[68] Yao B., Wang M., Zhang J. (2025). Imidazo[1,5-*a*]quinoxaline-8-Carboxamide Compounds as PRMTS Inhibitors and Their Preparation, Pharmaceutical Compositions and Use in the Treatment of Cancer. https://patents.google.com/patent/WO2025149020A1/en?oq=WO2025149020.

[69] Zhou Y. (2025). Preparation and Crystal Form of (Dihydrobenzofuranyl)imidazo[1,5-*a*]quinoxalinecarboxamide Derivative and Its Use in PRMT5 Inhibition. https://patents.google.com/patent/CN120535528A/en?oq=CN120535528.

[70] Eissa I.H., Metwaly A.M., Belal A., Mehany A.B.M., Ayyad R.R., El-Adl K., Mahdy H.A., Taghour M.S., El-Gamal K.M.A., El-Sawah M.E. (2019). Discovery and antiproliferative evaluation of new quinoxalines as potential DNA intercalators and topoisomerase II inhibitors. Arch. Pharm. Chem. Life Sci..

[71] Abbass E.M., Khalil A.K., Mohamed M.M., Eissa I.H., El-Naggar A.M. (2020). Design, efficient synthesis, docking studies, and anticancer evaluation of new quinoxalines as potential intercalative Topo II inhibitors and apoptosis inducers. Bioorg. Chem..

[72] Alsaif N.A., Taghour M.S., Alanazi M.M., Obaidullah A.J., Alanazi W.A., Alasmari A., Albassam H., Dahab M.A., Mahdy H.A. (2021). Identification of new [1,2,4]triazolo[4,3-*a*]quinoxalines as potent VEGFR-2 tyrosine kinase inhibitors: Design, synthesis, anticancer evaluation, and in silico studies. Bioorg. Med. Chem..

[73] Alsaif N.A., Dahab M.A., Alanazi M.M., Obaidullah A.J., Al-Mehizia A.A., Alanazi M.M., Aldawas S., Mahdy H.A., Elkady H. (2021). New quinoxaline derivatives as VEGFR-2 inhibitors with anticancer and apoptotic activity: Design, molecular modeling, and synthesis. Bioorg. Chem..

[74] Alsaif N.A., Taghour M.S., Alanazi M.M., Obaidullah A.J., Al-Mehizia A.A., Alanazi M.M., Aldawas S., Elwan A., Elkady H. (2021). Discovery of new VEGFR-2 inhibitors based on bis([1,2,4]triazolo)[4,3-*a*:3′,4′-*c*]quinoxaline derivatives as anticancer agents and apoptosis inducers. J. Enzym. Inhib. Med. Chem..

[75] Alsaif N.A., Mahdy H.A., Alanazi M.M., Obaidullah A.J., Alkahtani H.M., Al-Hossaini A.M., Al-Mehizi A.A., Elwan A., Taghour M.S. (2022). Targeting VEGFR-2 by new quinoxaline derivatives: Design, synthesis, antiproliferative assay, apoptosis induction, and in silico studies. Arch. Pharm..

[76] Alsaif N.A., Elwan A., Alanazi M.M., Obaidullah A.J., Alanazi W.A., Alasmari A.F., Albassam H., Mahdy H.A., Taghour M.S. (2022). Design, synthesis and molecular docking of new [1,2,4] triazolo[4,3-*a*]quinoxaline derivatives as anticancer agents targeting VEGFR-2 kinase. Mol. Divers..

[77] Almadani S.A., Elbokl M.T., Aljohani A.K.B., Alsulaimany M., Almohaywi B., Alharbi A.S., Alharthe A.M., Sedebaba A.A., Alsulimi F.H., El-morsy A. (2025). Nano-carrier for [1,2,4]triazolo[4,3-*a*]quinoxaline derivatives as inhibitors of Topo-II and intercalators for DNA: Design, synthesis, in silico docking, ADMET and anti-proliferative evaluations. J. Mol. Struct..

[78] Aljohani A.K.B., Almadani S.A., Alsulaimany M., Aljuhani N., Samman W.A., Al-Shareef A.H., Alghamdi R., Tayeb S.M., Alharbi H.Y., Aljohani M.S. (2025). Nano-carrier, design, synthesis, in silico ADMET, anti-proliferative assessments and docking of [1,2,4]triazolo[4,3-*a*]quinoxalines as Topo-II inhibitors and DNA intercalators. Naunyn-Schmiedeberg’s Arch. Pharm..

[79] Pandiri M., Nukala S.K., Dasari G., Badithapuram V., Bandari S. (2021). Design and Synthesis of Some New *N*-Phenyl-[1,2,4]triazolo[4,3-*a*]quinoxaline-1-sulfonamide Derivatives and Their Anti-Cancer Activity. Russ. J. Gen. Chem..

[80] Dasari G., Bandari S., Nukala S.K., Thirukovela N.S., Sirassu N., Badithapuram V., Manchal R. (2022). In vitro Anticancer and in silico Studies of Quinoxalinesulfonyl-1,2,4-triazole Hybrids. ChemistrySelect.

[81] Kaneko D., Ninomiya M., Yoshikawa R., Ono Y., Sonawane A.D., Tanaka K., Nishina A., Koketsu M. (2020). Synthesis of [1,2,4]triazolo[4,3-*a*]quinoxaline-1,3,4-oxadiazole derivatives as potent antiproliferative agents *via* a hybrid pharmacophore approach. Bioorg. Chem..

[82] Patinote C., Raevens S., Baumann A., Pellegrin E., Bonnet P.-A., Deleuze-Masquéfa C. (2023). [1,2,4]Triazolo[4,3-*a*]quinoxaline as Novel Scaffold in the Imiqualines Family: Candidates with Cytotoxic Activities on Melanoma Cell Lines. Molecules.

[83] Yuan C., Liu Y., Wang T., Sun M., Chen X. (2020). Nanomaterials as smart immunomodulator delivery system for enhanced cancer therapy. ACS Biomater. Sci. Eng..

[84] Al Ward M.M.S., Abdallah A.E., Zayed M.F., Ayyad R.R., El-Zahabi M.A. (2024). Design, synthesis and biological evaluation of newly triazolo-quinoxaline based potential immunomodulatory anticancer molecules. J. Mol. Struct..

[85] Ali I., Cha H.J., Lim B., Chae C.H., Youm J., Park W.J., Lee S.H., Kim J.H., Jeong D., Lim J.K. (2024). DW71177: A novel [1,2,4]triazolo[4,3-*a*]quinoxaline-based potent and BD1-Selective BET inhibitor for the treatment of acute myeloid leukemia. Eur. J. Med. Chem..

[86] Pierri M., Gazzillo E., Chini M.G., Ferraro M.G., Piccolo M., Maione F., Irace C., Bifulco G., Bruno I., Terracciano S. (2022). Introducing structure-based three-dimensional pharmacophore models for accelerating the discovery of selective BRD9 binders. Bioorg. Chem..

[87] Gazzillo E., Pierri M., Colarusso E., Chini M.G., Ferraro M.G., Piccolo M., Irace C., Bruno I., Bifulco G., Terracciano S. (2023). Exploring the chemical space of functionalized [1,2,4]triazolo[4,3-*a*]quinoxaline-based compounds targeting the bromodomain of BRD9. Bioorg. Chem..

[88] Pierri M., Bove G., Gazzillo E., Colarusso E., Scala F., Pepe G., Terracciano S., Chini M.G., Simonelli N., Bruno I. (2025). Unveiling New Triazoloquinoxaline-Based PROTACs Designed for the Selective Degradation of thencBAF Chromatin Remodeling Subunit BRD9. Chem. Eur. J..

[89] Serebrennikova P.O., Paznikova J.A., Kirnos E.A., Utepova I.A., Kazakova E.D., Lazarev V.F., Kuznetsova L.S., Margulis B.A., Guzhova I.V., Chupakhin O.N. (2023). Synthesis and biological evaluation of 1,2,4-triazoloazines as potent anticancer agents. New J. Chem..

[90] Sumran G., Aggarwal R., Mittal A., Aggarwal A., Gupta A. (2019). Design, synthesis and photoinduced DNA cleavage studies of [1,2,4]-triazolo[4,3-*a*]quinoxalin-4(5*H*)-ones. Bioorg. Chem..

[91] Shen Q.-K., Gong G.-H., Li G., Jin M., Cao L.-H., Quan Z.-S. (2019). Discovery and evaluation of novel synthetic 5-alkyl-4-oxo-4,5-dihydro-[1,2,4]triazolo[4,3-*a*]quinoxaline-1-carboxamide derivatives as anti-inflammatory agents. J. Enzym. Inhib. Med. Chem..

[92] Patil V.S., Yadavalli S.R., Merugu R., Swamy S.J., Devunuri N. (2021). One-pot, two-step synthesis of substituted triazoloquinoxalinone starting from 3-hydrazineylquinoxalin-2(1H)-one. Synth. Commun..

[93] Doğan I.S., Kahveci B., Sari S., Kolci K., Reis R., Sipahi H. (2022). Synthesis, Molecular Modelling and In Vitro Antiinflammatory Activity of Novel 1,2,4-Triazolo[4,3-*a*]quinoxaline Derivatives. ChemistrySelect.

[94] Serebrennikova P.O., Utepova I.A., Chupakhin O.N., Guzhova I.V., Mikhaylova E.R., Antonchick A.P. (2022). Synthesis and biological investigation of 1,2,4-triazolo[4,3-*a*]azines as potential HSF1 inductors. Synthesis.

[95] Jiang M.-Y., Han C., Zhang C., Zhou Q., Zhang B., Le M.-L., Huang M.-X., Wu Y., Luo H.-B. (2021). Discovery of effective phosphodiesterase 2 inhibitors with antioxidant activities for the treatment of Alzheimer’s disease. Bioorg. Med. Chem. Lett..

[96] Ezzat H.G., Bayoumi A.H., Sherbiny F.F., El-Morsy A.M., Ghiaty A., Alswah M., Abulkhair H.S. (2021). Design, synthesis, and molecular docking studies of new [1,2,4]triazolo[4,3-*a*]quinoxaline derivatives as potential A2B receptor antagonists. Mol. Divers..

[97] Harooni N.S., Naimi-Jamal M.R., Gharaghani S., Katebi A., Ajdary S., Tahghighi A. (2025). Identification of new triazoloquinoxaline amine derivatives with potent modulatory effects against Toll-like receptor 7 through pharmacophore-based virtual screening and molecular docking approaches. PLoS ONE.

[98] Hou H., Yang R., Liu X., Wu X., Zhang S., Chen K., Zheng M. (2020). Discovery of triazoloquinoxaline as novel STING Agonists via Structure-based Virtual Screening. Bioorg. Chem..

[99] Omar A.M., Alswah M., Ahmed H.E.A., Bayoumi A.H., El-Gamal K.M., El-Morsy A., Ghiaty A., Afifi T.H., Sherbiny F.F., Mohammed A.S. (2020). Antimicrobial screening and pharmacokinetic profiling of novel phenyl-[1,2,4]triazolo[4,3-*a*]quinoxaline analogues targeting *DHFR* and *E. coli* DNA gyrase B. Bioorg. Chem..

[100] Debbert S.L., Hintz M.J., Bell C.J., Earl R., Forsythe G.E., Häberli C., Keiser J. (2021). Activities of Quinoxaline, Nitroquinoxaline and [1,2,4]Triazolo[4,3-*a*]quinoxaline Analogs of MMV007204 against *Schistosoma mansoni*. Antimicrob. Agents Chem..

[101] Chen W., Tu X., Xu M., Chu Y., Zhu Y. (2021). Copper(I) Iodide Catalyzed Tandem Reactions of *N*-Propargyl-*N*-(2-iodoaryl)amides with Sodium Azide: An Efficient Synthesis of [1,2,3]Triazolo[1,5-*a*]quinoxalines. Synlett.

[102] Pelliccia S., Alfano A.I., Da Assunção B.R.G., Turco L., Lembo F., Summa V., Buommino E., Brindisi M. (2023). Rejuvenating the [1,2,3]-triazolo[1,5-*a*]quinoxalin-4(5*H*)-one scaffold: Synthesis and derivatization in a sustainable guise and preliminary antimicrobial evaluation. Front. Chem..

[103] Masaryk B., Soural M. (2024). Metal-Free Synthesis of Polysubstituted Triazoloquinoxalines Using Alkynols as the Key Building Blocks. ACS Omega.

